# Predicting Ligand Binding Sites on Protein Surfaces by 3-Dimensional Probability Density Distributions of Interacting Atoms

**DOI:** 10.1371/journal.pone.0160315

**Published:** 2016-08-11

**Authors:** Jhih-Wei Jian, Pavadai Elumalai, Thejkiran Pitti, Chih Yuan Wu, Keng-Chang Tsai, Jeng-Yih Chang, Hung-Pin Peng, An-Suei Yang

**Affiliations:** 1 Genomics Research Center, Academia Sinica, Taipei, Taiwan 115; 2 Institute of Biomedical Informatics, National Yang-Ming University, Taipei, Taiwan 11221; 3 Bioinformatics Program, Taiwan International Graduate Program, Institute of Information Science, Academia Sinica, Taipei, Taiwan 115; 4 Institute of Bioinformatics and Structural Biology, National Tsing Hua University, Hsinchu, Taiwan 30013; University of Michigan, UNITED STATES

## Abstract

Predicting ligand binding sites (LBSs) on protein structures, which are obtained either from experimental or computational methods, is a useful first step in functional annotation or structure-based drug design for the protein structures. In this work, the structure-based machine learning algorithm ISMBLab-LIG was developed to predict LBSs on protein surfaces with input attributes derived from the three-dimensional probability density maps of interacting atoms, which were reconstructed on the query protein surfaces and were relatively insensitive to local conformational variations of the tentative ligand binding sites. The prediction accuracy of the ISMBLab-LIG predictors is comparable to that of the best LBS predictors benchmarked on several well-established testing datasets. More importantly, the ISMBLab-LIG algorithm has substantial tolerance to the prediction uncertainties of computationally derived protein structure models. As such, the method is particularly useful for predicting LBSs not only on experimental protein structures without known LBS templates in the database but also on computationally predicted model protein structures with structural uncertainties in the tentative ligand binding sites.

## Introduction

One essential step in predicting the function of an unannotated protein and in identifying key residues involving the protein’s biological role is to predict the ligand binding site (LBS) based on the protein’s sequence and structure. One important application is to use the predicted LBS in connection with the protein’s structure, derived with experimental or computational methods, for structure-based development of pharmacological compounds binding to the tentative LBS. As more than three quarters of proteins with less than 1000 residues in human proteome can be modeled with sufficient accuracy with contemporary structure prediction methods [1], computational LBS prediction approaches exploiting ever-increasing sequence and structural information on human proteome can now aim at structure-based drug development for target proteins with insufficient experimental information on the structures and functions of the proteins.

The essence of protein LBSs resides in the geometry of the binding site and chemical composition lining the ligand-contact surface. The LBS geometries are diverse but not unlimited in variations, represented by around 1000 pocket shapes. However, similar pockets could accommodate very different ligand scaffolds, and similar ligands could bind to pockets with very different geometries [2]. The complexity in the chemical composition of the LBS linings is not less daunting: statistical propensities for amino acids are not strikingly different between the LBS linings and the general protein surfaces exposed to solvent, although amino acid side chains, especially Trp, Tyr, Arg, are slightly statistically abundant in binding to ligands [3]. Together, the conclusions of the analyses on protein LBSs have emerged to suggest that amino acid residue compositions and pocket shapes are necessary, if not sufficient, attributes in identifying LBSs on protein surfaces [3].

Many protein LBS prediction algorithms have been developed, as summarized in the reviews [4–8]. The classical heuristic approach is based on evolutionary methods that exploit the propensity of conserved residues in the binding site [9, 10]. In contrast to the evolutionary methods, the structure-based methods predict LBSs on protein surfaces by analyzing geometrical features like cleft or cavity [11–16]. To overcome the limitations of geometry-based methods, energy-based approaches have been developed to predict the binding sites on proteins using interaction energy calculations [17–20]. Template-based methods utilize interaction information from template proteins to infer the binding information on the query proteins at global and/or local level [21–25]. As different methods have different strengths and weaknesses, combinations of more than one established methods have been developed to improve the prediction accuracy [26–30]. The use of residue conservation information further improves the binding site prediction [11, 31, 32].

The applicability of protein LBS predictions for protein function annotation and computational drug design remains to be further improved. Based on the recent CASP assessment, the most accurate protein LBS prediction methods were largely dependent on finding template protein structures with LBSs defined through high resolution experimental methods [33]. But these template-based approaches have had limitation, as revealed in the analysis that less than 25% of protein pairs with highly similar structures but moderate sequence identity in the twilight zone share common LBSs [34]. Moreover, holo protein structures are relatively scarce in the protein databank (PDB) because of the associated experimental difficulties, leading to the further limitation that only about a quarter of human proteome can be inferred in ligand binding information from protein-ligand complexes in PDB [1]. The fact that large structural variations among distantly related proteins binding to similar or identical ligands frequently occur in nature [35] further complicates the prediction algorithms based on inferences of known template structures. Hence LBS prediction methods that are not dependent on trivial templates of known structures need to be further developed [33].

In this work, a protein structure-based LBS prediction method (ISMBLab-LIG) was developed to predict LBSs with geometry features and physicochemical properties of protein surface atoms without inferred information from template structures. To exploit rapidly expanding volume of computationally modeled protein structures, the LBS prediction method was developed with applicability extending to protein models with imperfect accuracy to an extent. Unlike the LBS predictors summarized above, the ISMBLab-LIG algorithm predicts the likelihood of a protein surface atom to be involved in a LBS with one of the 30 artificial neural network (ANN) models trained separately for each of the 30 protein atom types. Each of the ANN models has input of 54 attributes, derived from 53 three-dimensional probability density maps (PDMs) describing the distributions of 53 interacting atom types around the protein surface atom plus one geometry attribute describing the protein surface atom’s local geometry [36–38]. The outputs of the ANN models are normalized into prediction confidence levels ranging from 0 to 1; the LBSs are predicted by integrating the protein surface atoms into patches based on the normalized prediction confidence levels for the protein surface atoms. The predicted LBS confidence level is linearly correlated with prediction accuracy, which was benchmarked with Matthews correlation coefficient (MCC), as suggested for LBS predictor development in a recent CASP assessment [33]. 10-fold cross validation of the training set containing more than 5000 known protein-ligand complexes yielded overall MCC of 0.50 at the residue-based predictions. The ISMBLab-LIG predictors were further validated with well-established independent test datasets (protein structures with bound or unbound ligands) and CAMEO-LB targets [39]. The independent tests indicate that the ISMBLab-LIG prediction accuracy for protein LBSs is comparable to the most notable benchmarks published. When modeled protein structures were used for LBS predictions, LBS structural variation of less than 5 Å could be routinely tolerated in reproducing reasonably accurate predictions, and in many cases, structural variation of more than 10 Å in the LBSs did not result in substantial shift of the LBS predictions in the model structures. The prediction benchmarks indicate that the ISMBLab-LIG is useful in LBS prediction as first step in structure-based drug design or functional annotation for proteins of unknown function with structures derived from experimental or computational methods.

## Results

### Atom-based LBS predictions with ISMBLab-LIG

**Fig 1:**
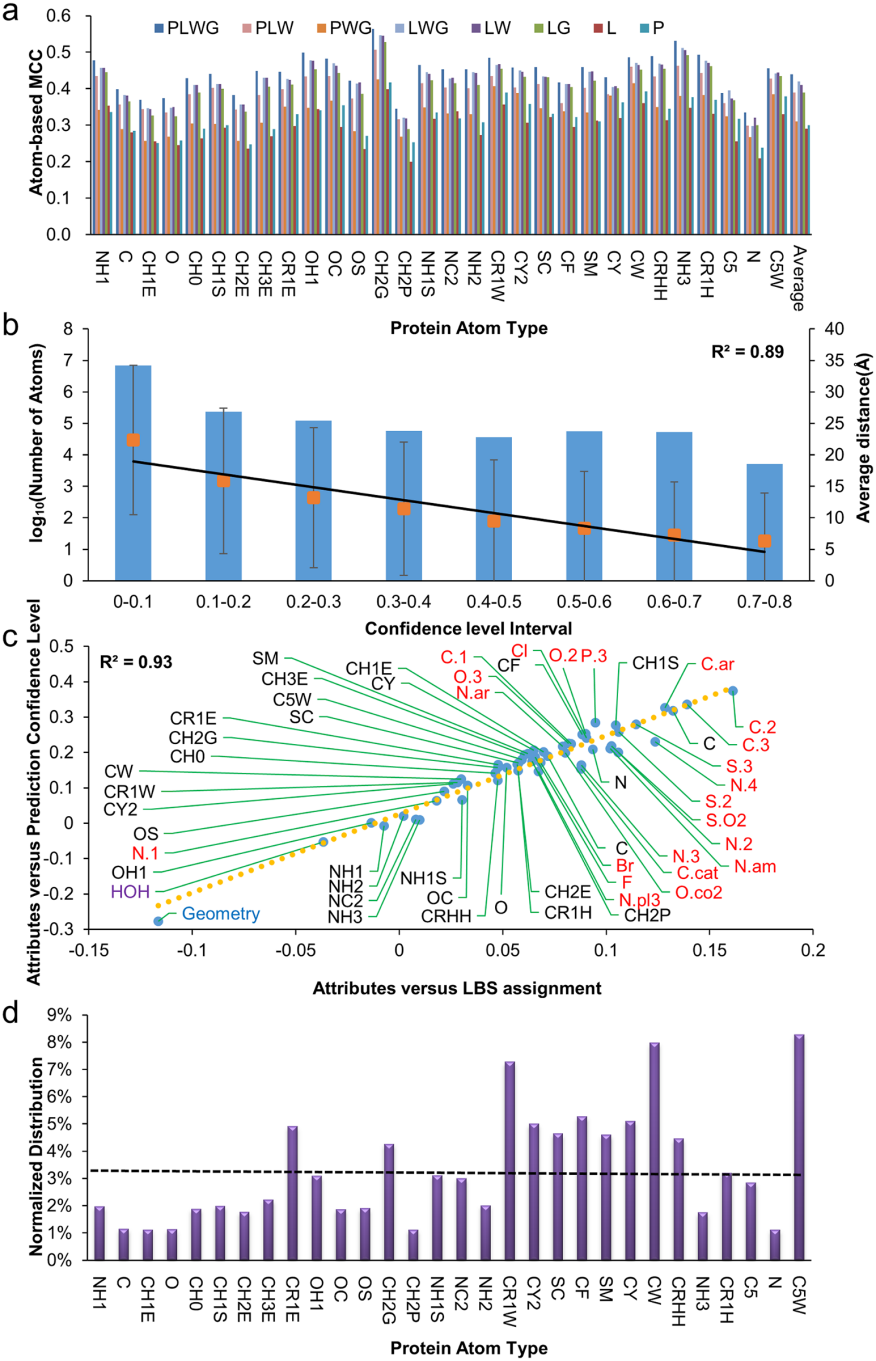
Ligand binding site predictions at atom-based level. **(a)** The prediction accuracies for the 30 ANN_BAGGING models (x-axis) trained with different subsets of the 54 attributes (Table 1) are evaluated with MCCs (y-axis). For each of the ANN_BAGGING models, 8 different combinations of the 54 attributes were used as input sets for machine learning algorithm: PLWG-attributes 1~54; PLW-attributes 1~53; PWG-attributes 1~31 and 54; LWG-attributes 31~54; LW- attributes 31~53; LG-attributes 32~54; L-attributes 32~53; P-attributes 1~30. **(b)** The correlation of the normalized prediction confidence level of protein surface atoms (x-axis) to the average distance (organ squares with standard deviations) from the protein surface atoms to the corresponding ligands (right-hand side y-axis) is shown by the linear fitting of the data points (R^2^ = 0.89). The blue histogram shows the distributions of the protein surface atoms in log10 scale (left-hand side y-axis) for each prediction confidence level range. **(c)** The Pearson’s correlation coefficients (PCCs) between attribute values and prediction confidence levels are shown in the y-axis. The PCCs between attribute values and LBS assignments are shown in the x-axis. The interacting protein atom types are labelled in black; the interacting ligand atom types are labelled in red; the interacting water atom type is labelled in purple; and the geometry attribute is labelled in blue. See text for discussion. **(d)** Distribution of protein atom types (x-axis) in the predicted atom-based ligand binding sites in proteins is shown by the histogram. The percentage *P*_*i*_ (shown in the y-axis) for the atom type *i* is calculated by the equation below: $P_{i}\;=\frac{p_{i}}{\sum_{j=1}^{30}p_{j}}$, where $p_{i}=\frac{n_{i}}{N_{i}}$ *N*_*i*_ is the total number of atom type *i* in the dataset, while *n*_*i*_ is the number of atom type *i* with prediction confidence level greater than 10%. The dashed line in the figure represents the baseline (*P*_*i*_ = 1/30) for random predictions. The data shown in the panels (a) to (d) were calculated with the predictions in the 10-fold cross validation on the S5010 dataset.

ISMBLab-LIG is a structure-based LBS prediction algorithm based on artificial neural network. As described in the Methods and the S1 Text, the ISMBLab-LIG contains 30 ANN_BAGGING neural network models, each of which uses 54 attributes (see Methods and Eqs 2–4 in S1 Text) as input and one node to output prediction activity. The ANN_BAGGING artificial neural network algorithm (S1 Text) was chosen among several machine learning algorithms based on the prediction performances shown in previous works [37, 38]. 10-fold cross validation of the ISMBLab-LIG predictors with the S5010 dataset indicated the overall Matthews correlation coefficient (MCC) of the predictions is 0.44 at atom-based level (Fig 1a). In each of the 10-fold cross validations, 80% of the protein surface atoms in the S5010 dataset were used as the training set; 10% of the remaining protein surface atoms in the S5010 dataset were used as the validation set to determine the prediction activity thresholds for positive predictions, and the remaining 10% of the dataset were used as the testing set. The accuracy of the prediction results was evaluated with MCC (Eq 6 in Methods), where the true positives (TPs) are the atoms predicted to be positive (above an optimized activity threshold) and are within 4.5 Å of any heavy atom on the bound ligand. Fig 1a shows the prediction accuracies for the 30 predictors and the averaged prediction accuracy.

The accuracy of the predictions for each of the 30 predictors depends on the completeness of the input attributes. For all the 30 predictors, predictions using only the attributes from interacting protein atom types (ID number from 1 to 30 in Table 1) or only the attributes from interacting ligand atom types (ID number from 32 to 53 in Table 1) had MCCs about one quarter inferior to the corresponding MCC of the predictions using the full set of 54 attributes (Fig 1a). Moreover, the information encoded in the attributes of water (ID number 31 in Table 1) and geometry (the 54^th^ attribute) contributed substantial prediction accuracy (Fig 1a). The results shown in Fig 1a suggest that all the 54 attributes provide non-redundant information for the LBS predictions.

**Table 1:** The definition of protein and ligand atom types.

IDNo.	AtomType	Radius(Å)	Description
1	NH1	1.65	Backbone NH
2	C	1.76	Backbone C
3	CH1E	1.87	Backbone CA(exc. Gly)
4	O	1.40	Backbone O
5	CH0	1.76	Arg CZ, Asn CG, Asp CG, Gln CD, Glu CD
6	CH1S	1.87	Side chain CH1:Ile CB, Leu CG, Thr CB, Val CB
7	CH2E	1.87	Tetrahedral CH2(except CH2P,CH2G) All CB
8	CH3E	1.87	Tetrahedral CH3
9	CR1E	1.76	Aromatic CH(except CR1W,CRHH,CR1H)
10	OH1	1.40	Alcohol OH(Ser OG, Thr OG1,Tyr OH)
11	OC	1.40	CarboxylO(Asp OD1,OD2,Glu OE1,OE2)
12	OS	1.40	Side chain O: Asn OD1,Gln OE1
13	CH2G	1.87	Gly CA
14	CH2P	1.87	Pro CB,CG,CD
15	NH1S	1.65	Side chain NH: Arg NE, His ND1,NE1,Trp NE1
16	NC2	1.65	Arg NH1,NH2
17	NH2	1.65	Asn ND2,Gln NE2
18	CR1W	1.76	Trp CZ2,CH2
19	CY2	1.76	Tyr CZ
20	SC	1.85	Cys S
21	CF	1.76	Phe CG
22	SM	1.85	Met S
23	CY	1.76	Tyr CG
24	CW	1.76	Trp CD2,CE2
25	CRHH	1.76	His CE1
26	NH3	1.50	Lys NZ
27	CR1H	1.76	His CD2
28	C5	1.76	His CG
29	N	1.65	Pro N
30	C5W	1.76	Trp CG
31	HOH	1.40	Water
32	C.3	1.91	sp3 carbon
33	C.2	1.91	sp2 carbon
34	C.1	1.91	sp carbon
35	C.ar	1.91	aromatic carbon
36	C.cat	1.91	carbocation used only in a guadinium group
37	O.3	1.68	sp3 oxygen
38	O.2	1.66	sp2 oxygen
39	O.co2	1.66	oxygen in carboxylate or phosphate group
40	N.4	1.82	sp3 positively charged nitrogen
41	N.3	1.82	sp3 nitrogen
42	N.2	1.82	sp2 nitrogen
43	N.1	1.82	sp nitrogen
44	N.ar	1.82	aromatic nitrogen
45	N.pl3	1.82	trigonal planar nitrogen
46	N.am	1.82	amide nitrogen
47	P.3	2.10	sp3 phosphorous
48	S.3	2.00	sp3 sulfur
49	S.2	2.00	sp2 sulfur
50	S.O2	2.00	Sulfone sulfur
51	F	1.75	F fluorine
52	Cl	1.95	Cl chlorine
53	Br	2.22	Br bromine

The protein atom types (1–31) have been previously defined.

Prediction activity (output from ANN_BAGGING models) with value ranging from 0 to 1 was normalized to prediction confidence level so that the prediction results from the 30 predictors of ISMBLab-LIG can be compared on a leveled ground, allowing the prediction results to be integrated into ligand binding patches (Methods) [37, 38]. Fig 1b shows the linear correlation (R^2^ = 0.89) of the normalized prediction confidence level (x-axis) and the shortest distance of the query protein surface atom to the corresponding ligand (y-axis, right-hand side) in the S5010 dataset–the query protein surface atoms with higher normalized prediction confidence levels are closer to the corresponding ligand binding sites on average. The result shown in Fig 1b indicates that the normalized prediction confidence level from the 30 ISMBLab-LIG predictors reasonably reflects the LBS prediction accuracy.

Among the 54 attributes, the attribute values from ligand carbon atoms and the attribute value derived from the query protein atom’s local geometry are more correlated to the actual ligand binding, and thus contributed more to their prediction confidence levels. Associated with each protein surface atoms are 54 attribute values, prediction confidence level for the atom to involve in LBS, and the LBS assignment of 1 (positive assignment) or 0 (negative assignment) determined by the threshold of 4.5 Å to any heavy atom on the corresponding ligand. The interacting atom type’s attribute values are correlated with the prediction confidence levels or with the LBS assignments to different extent. Higher correlation between the attribute values and the prediction confidence levels should imply higher correlation to the LBS assignments as well, and the interacting atom types with higher correlations among the attribute values, prediction confidence levels, and LBS assignments are expected to contribute more weights to the prediction accuracies. Fig 1c plots one set of Pearson’s correlation coefficients (PCC) versus the other set: the PCCs shown in the x-axis are the correlation coefficients between the attribute values of the interacting atom types (as indicated next to the data points) and the LBS assignment; the PCCs shown in the y-axis are the correlation coefficients between the attribute values of the interacting atom types (as indicated next to the data points) and the prediction confidence levels. Indeed, the linear correlation (R^2^ = 0.93) in the plot suggests that attribute values correlate better to the assignment of actual ligand binding (higher PCC shown in x-axis) should also correlate better with prediction confidence level (higher PCC shown in y-axis), indicating that attribute values of different interacting atom types contribute differently in predicting actual ligand binding and that the attributes with higher correlations with prediction confidence levels contribute more statistical weights in predicting the actual ligand binding sites. These determinant attributes are mostly derived from the interacting ligand atom types (labelled in red characters in Fig 1c), especially C.2, C.3, C.ar, and to lesser extent, N.4, S.3, and S.2 (Fig 1c and Table 1). The results shown in Fig 1c indicate that ligand atom type attributes provide directly correlated connection between the query atom and the ligand atom types it could interact–higher ligand atom type attributes indicate higher predicted confidence level for the query protein atom to involve in a ligand binding site. The determinants of the ligand atom type attributes are originated from the information encoded in the attributes from the database recording the atomistic pairwise interactions between organic ligands and the protein surface atoms similar to the query atom. The geometry attribute, which is negatively correlated to both prediction confidence level and the LBS assignment, is also expected to contribute substantially to the prediction capabilities. The result shown in Fig 1c suggests that the ISMBLab-LIG predictors identify LBS by mostly recognizing concave pockets on protein surface with higher distribution density of the ligand atoms (in particularly, the ligand carbon atoms C.2, C.3, and C.ar).

The protein-ligand binding sites predicted with the ISMBLab-LIG predictors are distinguishable in atom type distributions comparing with average protein surfaces. Fig 1d shows the distribution of the protein atom types in predicted LBSs from the S5010 dataset. The distribution is normalized by the distribution of protein surface atoms, so that the distribution ratio of 1/30 for all protein atom types indicates the binding site atom type has the distribution ratio indistinguishable from the average protein surfaces (shown by the dashed line in Fig 1d). The result of Fig 1d shows that protein surface atoms with LBS prediction confidence level greater than 10% are mostly composed of aromatic carbons, cysteine/methionine sulfur, glycine Cα’s, and to a lesser extent, arginine and histidine sidechain atoms and hydroxyl groups on polar sidechains. This result is in general agreement with the statistical surveys of preferred protein amino acid types in protein-ligand interaction sites [3].

### Performance benchmarks for the residue-based LBS predictions with ISMBLab-LIG

The prediction performance of the ISMBLab-LIG was further benchmarked at residue-based level with previously published datasets as described in the Methods section, allowing comparisons of prediction performances with other LBS prediction methods. The residue-based LBS prediction output method is described in the Methods section. The residue-based prediction accuracy, precision, sensitivity, specificity, F-score, and MCC are defined in Eqs 1–6 respectively. The averaged benchmarking results for the training set (S5010) and the testing sets are summarized in Table 2. The detailed prediction benchmarks for each of the structures in the testing datasets (S48b, S48ub, S210, S198, and S523) are shown in S1–S5 Tables respectively.

**Table 2:** ISMBLab-LIG residue-based prediction performances benchmarked with published datasets.

Dataset	Accuracy	Precision	Sensitivity	Specificity	MCC^a^	MCC^b^	F-score
**S5010**	0.947	0.513	0.546	0.97	0.501	N/A	0.529
**S48b**	0.954	0.562	0.625	0.972	0.568	0.575	0.592
**S48ub**	0.955	0.521	0.545	0.975	0.509	0.5	0.533
**S210**	0.944	0.568	0.468	0.976	0.487	0.505	0.513
**S198**	0.948	0.323	0.53	0.962	0.389	0.382	0.402
**S523**	0.948	0.449	0.566	0.966	0.477	0.507	0.501

The datasets are described in the Methods section.

^a^: The MCCs were calculated with the definition of the actual LBS residues described in the Methods section.

^b^: The MCCs were calculated with the actual LBS residues, where each of the actual LBS residues contains at least one heavy atom within the distance of the sum of Van der Waals radii plus the tolerance distance (0.5 Å) to any ligand heavy atom [21, 33].

**Fig 2:**
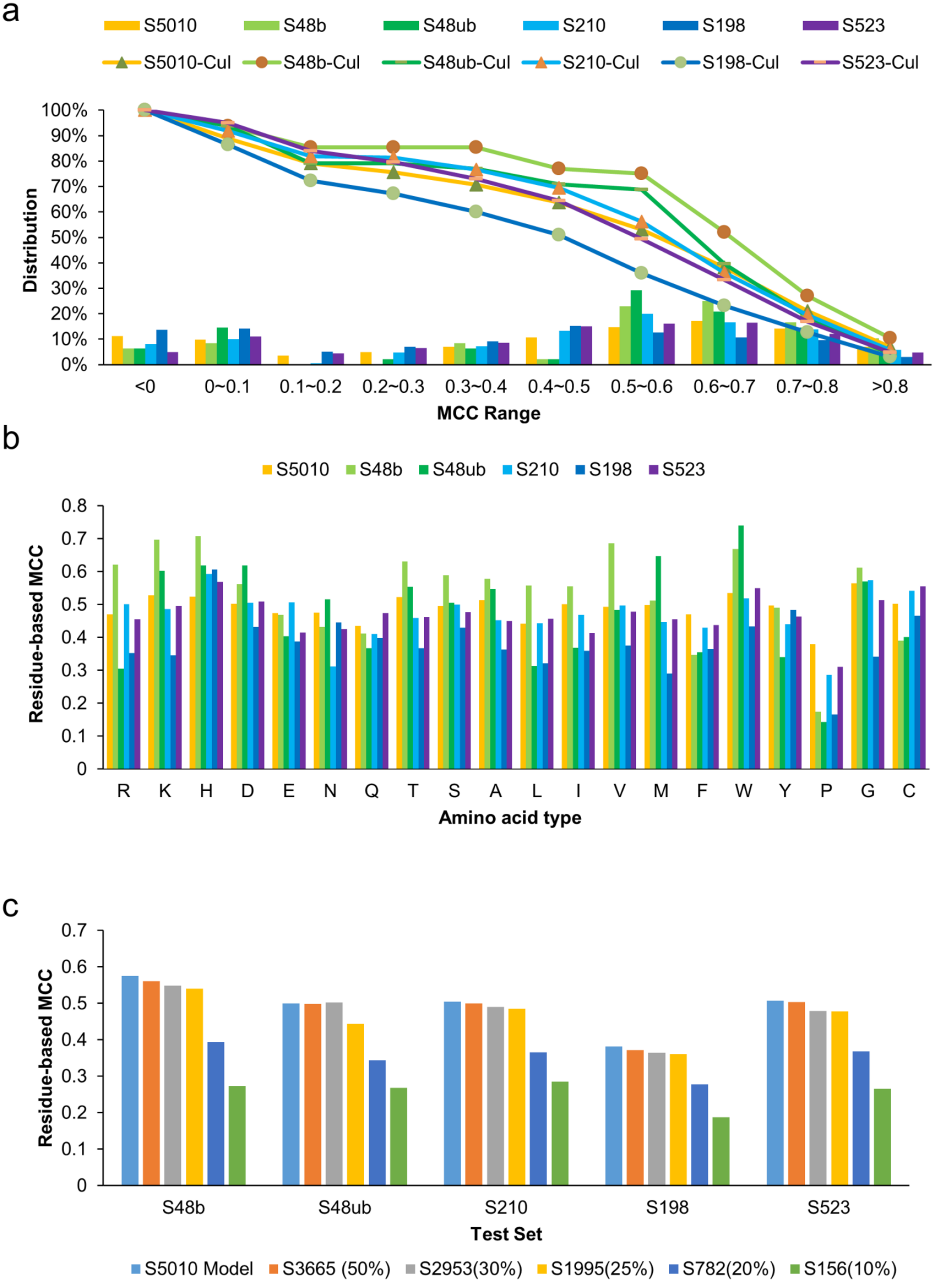
Benchmarking ligand binding site prediction accuracies at residue-based level. **(a)** The distributions of the prediction performances on the training set (S5010) and testing sets (S48b, S48ub, S198, S210, and S523) are shown by the histograms with the MCC ranges shown in the x-axis. The independent tests were carried out with the trained ISMBLab-LIG predictors showing the best prediction performance from the 10-fold cross validation on the S5010 dataset. The curves show the cumulative percentage of the corresponding datasets predicted with the residue-based MCC values greater than the central value of the MCC range shown in the x-axis. Detailed prediction results for each of the testing sets are shown in S1–S5 Tables. **(b)** Averaged residue-based MCCs (y-axis) for each of the 20 amino acid types (x-axis) are shown for the training and testing sets. The MCCs for the training set are averaged over the 10-fold cross validation. **(c)** Prediction accuracies (averaged residue-based MCCs, y-axis) for the 5 testing sets are shown for the ISMBLab-LIG predictors trained with 6 training sets (S5010, S3665, S2953, S1995, S782, S156, see text). The prediction accuracy is defined as MCC^b^ in Table 2.

The prediction performance comparisons indicate that the prediction capability of ISMBLab-LIG has been generalized beyond the training dataset. The predictor performances as measured with residue-based MCC (see Methods) on the 10-fold cross validation training/testing set S5010 are generally reproducible in the 5 testing sets, as shown in Fig 2a, where the MCC distributions have similar trends among the datasets, especially for the datasets with large number of testing cases (S523, S198, and S210). This result indicates that the ISMBLab-LIG predictors, trained with the S5010 data set, are generalizable to protein structures that have not been included in the training set (S5010) and to proteins structures without the bound ligands (S48b vs. S48ub).

Fig 2b indicates that the prediction MCCs have generally similar dependence on the amino acid types among the benchmark datasets. This result further support the generalizability of the ISMBLab-LIG predictors to protein structures unseen in the training dataset. Moreover, the amino acid types with higher prediction MCC (Fig 2b) are consistent with the protein atom types that are more abundant in the LBSs (Fig 1d): these amino acid types, including His, Asp, Trp, Tyr, Gly, and Cys, are also consistent in general with the surveys indicating the abundance of these amino acid types in LBSs [3]. These results suggest that the machine learning of ISMBLab-LIG has better generalizability to the protein atom types with more positive training cases.

To challenge the possibility that the prediction benchmarks (Table 2 and Fig 2a and 2b) could be originated from the evolutionary relationships between the training proteins and the testing proteins, we retrained the ISMBLab-LIG predictors with subsets of the training proteins in S5010. 5 subsets containing proteins increasingly distant from all the testing proteins in the 5 testing sets were derived from the original training set S5010: S3665 (3666 training proteins), S2953 (2953 training proteins), S1995 (1995 training proteins), S782 (782 training proteins), S156 (156 training proteins) contain training proteins that are not related to any of the testing proteins in all the 5 testing sets by more than 50%, 30%, 25%, 20% and 10% sequence ID respectively. The performance of the ISMBLab-LIG predictors benchmarked with the 5 testing sets deteriorated substantially only when the predictors were trained with the S782 and S156 training set respectively (Fig 2c), perhaps due to insufficient training cases to generalize the predictor models. The results indicate that the ISMBLab-LIG predictors are generalizable to the testing proteins that share no more than 25% sequence ID with the training proteins; the generalizability could extend to even lower sequence ID threshold if enough training proteins had been available. The result indicates that the ISMBLab-LIG predictors catch the general protein-ligand interaction features, rather than relying on closely related template LBSs in the structural database. As such, it is expected that the ISMBLab-LIG has more tolerance of input structural uncertainty than template-based LBS prediction methods.

**Fig 3:**
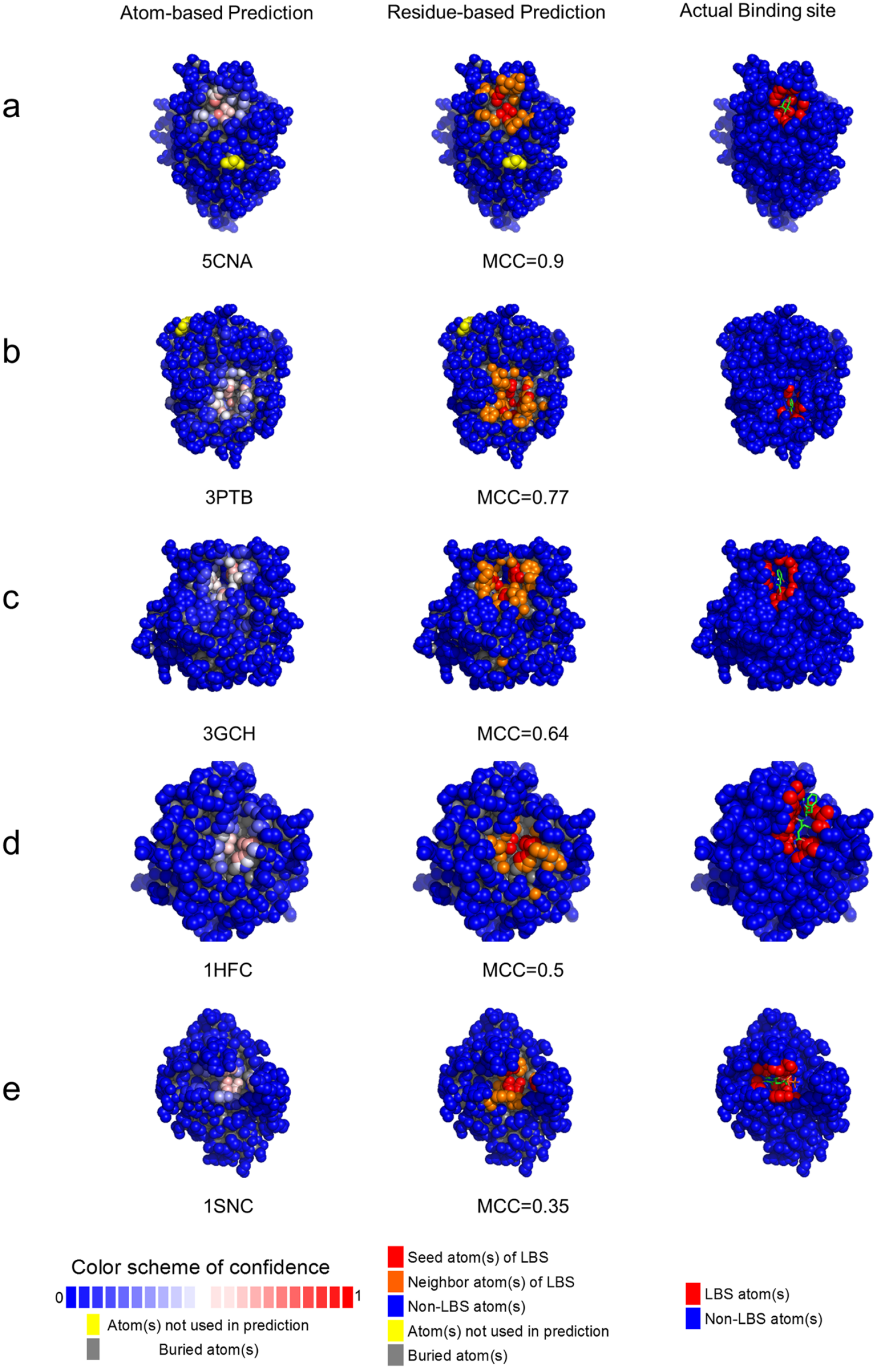
Examples of ligand binding site predictions in S48b testing set. Panels (a) to (e) show the prediction examples of five proteins from S48b testing set with MCC of 0.9, 0.77, 0.64, 0.5 and 0.35, respectively. The left-hand side structures are color-coded by atom-based prediction confidence level. The color bar at the bottom of this column shows the color scheme for normalized prediction confidence level. The seed atoms are colored in red of various level of depth. The protein structures of the middle column show the residue-based ligand binding site predictions. The residues colored in red or orange represent the positive residues of the predicted patches in the ligand binding sites. The red atoms were predicted with prediction confidence level greater than 0.5; other atoms in the positive residues of predicted patches with prediction confidence level less than 0.5 are colored in orange. The right-hand side structures show the surface atoms in close contact with the ligands. The atom colored in red are within 4.5 Å distance to any heavy atom of corresponding ligand. The PDB code name and the MCC for each of the examples are also shown. The complete prediction benchmarks for all testing sets are available for interactive examination from the ISMBLab web server: http://ismblab.genomics.sinica.edu.tw/>benchmark>Protein-Ligand.

About 70% of the LBS predictions from the ISMBLab-LIG predictors are highly relevant in identifying actual LBSs. Fig 3 compares the ISMBLab-LIG predicted atom-based patches and residue-based patches with actual ligand-binding residues for randomly selected cases with various prediction accuracy indicated by MCC. As shown in the figure, LBS predictions with MCC above 0.3 remain substantially relevant to the actually ligand binding patches. About 70% of the cases in the datasets shown in Fig 2a have LBS prediction MCC above 0.3. This result suggests that the ISMBLab-LIG predictions are highly relevant to the actual ligand binding sites on proteins.

Still, there are about 20% of the test cases for which the LBS predictions cannot generate useful results (MCC<0.1, Fig 2a). Many of these LBSs are deeply buried and are not accessible as surface patches. These binding sites cannot be predicted by ISMBLab-LIG, which requires that LBSs are composed of protein surface atoms. Other failed cases involve LBSs that are not the typical concave pockets, where the machine leaning models of ISMBLab-LIG fail to predict as LBSs with high confidence. Similarly, combinations of amino acid types that are rarely observed in the majority of ligand binding sites are frequently predicted as false negatives.

The prediction accuracy of ISMBLab-LIG is comparable to that of the best LBS predictors. Detailed prediction results for all the cases in the testing datasets (S48b, S48ub, S210, S198, S523) can be found in S1–S5 Tables respectively. The success rate has been defined as the fraction of the prediction cases with the predicted geometry center of the top one predicted LBS patch within 4Å of the corresponding ligand [11, 20, 27]. The comparisons of success rates for ISMBLab-LIG with other methods are summarized in S6–S8 Tables respectively. S6 Table compares the success rate for top 1 predicted site by ISMBLab-LIG with other methods for S48b and S48ub sets. ISMBLab-LIG’s performance (85%) was second to that of LISE (92%) in S48b and equal to VICE and MPK2, and was equally top with VICE (83%) in S48ub [11, 20, 27]. In the S210 testing set (S7 Table), the success rate of ISMBLab-LIG is 84%, which ranks as the first among 9 predictors [11, 20, 27]. For S198 dataset shown in S8 Table, ISMBLab-LIG’s performance (55%) was second to that of MPK2 (61%) and equal to MPK1 [28].

In the S523 dataset, the averaged MCC for the top one ISMBLab-LIG predictions is 0.51 (Table 2), in comparison with the MCC of 0.48 from the COFACTOR predictions using a LBS template database of 30% or less sequence ID to the test cases [21]. Both MCCs were calculated with the actual LBS residues, where each of the actual LBS residues contains at least one heavy atom within the distance of the sum of van der Waals radii plus the tolerance distance (0.5 Å) to any ligand heavy atom [21, 33].

### Comparison of ISMBLab-LIG with COACH, RaptorX and COFACTOR on LBS predictions for targets from CAMEO-LB

To compare the ISMBLab-LIG predictors with the prediction methods assessable in CAMEO-LB webserver [39], we predicted LBSs on CAMEO-LB targets and compared the prediction results with those from the best performers in CAMEO-LB predictions (COACH [40] and RaptorX (http://raptorx.uchicago.edu/documentation/#goto2)) and COFACTOR [25]. The CAMEO-LB webserver released 222 target sequences between 2016/3/26 and 2016/4/25; only 22 target sequences were labelled as binding with at least one organic ligand and were non-redundant with 90% sequence ID threshold. I-TASSER standalone package was used to build models for these 22 target sequences with default parameters [41].

Only the top 1 predicted results from each of the 4 prediction algorithms are compared in S1 Fig. COFACTOR in-house version (Mar/2016) was used to predict the top 1 LBSs on the 22 target models. The top 1 LBS predicted by COFACTOR is the LBS residues on the query model determined by the template cluster with the highest confidence score (C-score) binding to an organic ligand; the C-score is determined by the combinations of local and global structural similarity and sequence identity between the query and the templates [25]. The 22 target models were also submitted to COACH webserver (http://zhanglab.ccmb.med.umich.edu/COACH/) and the sequences of the 22 targets were submitted to RaptorX webserver (http://raptorx.uchicago.edu/BindingSite/), which does not accept protein structures as input. The top 1 LBS predicted by COACH is the LBS residues from the template cluster with the highest confidence score binding to organic ligand; the COACH confidence score is derived from optimal combination of five prediction outcomes (S-SITE, TM-SITE, FINDSITE, COFACTOR, and ConCavity) with a linear-SVM algorithm for combination weight determination [40]. The top 1 LBS predicted by RaptorX is the LBS residues of the highest ranked pocket binding to organic ligand; the rank is determined by pocket multiplicity representing the frequency with which the selected pocket was found in a set of ligand-binding protein structures (http://raptorx.uchicago.edu/documentation/#goto2). The top 1 LBS prediction results with the 4 prediction algorithms are compared with the actual LBS residues in S10 Table.

The prediction results are compared in S1 Fig. According to the CAMEO-LB webserver, three scoring systems were used to benchmark prediction accuracy: BDT [42] (Eqs 7 and 8 in Methods), MCC, and AUC. The ISMBLab-LIG LBS predictions are not compatible with AUC calculation and the AUC scores are not available for COFACTOR (in house version 2016/3) predictions. Hence, the AUC score is not used in this comparison. Three cases were not predicted with MCC > 0 by all four predictors (panels a, f, and l in S1 Fig). For the remaining 19 cases, COACH performed the best according to the BDT score: with 8 best prediction cases, each of which has the highest BDT score among the four predictors (S1 Fig). ISMBLab-LIG is the second with 5 best prediction cases among the four predictors, followed by RaptorX and COFACTOR with 4 and 3 best predictions (S1 Fig). When MCC used as the benchmark, COACH remained the best performer with 8 best predictions among the four predictors, followed by RaptorX, COFACTOR, and ISMBLab-LIG with 6, 5, and 1 best predictions among the four predictors respectively (S1 Fig). In summary, COACH predictions based on the consensus of 5 predictors consistently out-performed the other three predictors. ISMBLab-LIG performed better than COFACTOR and RaptorX with BDT score as the benchmark, but performed the worst among the three predictors with MCC as the benchmark.

### LBS predictions with tolerance of structural uncertainties in comparatively modeled structures

**Fig 4:**
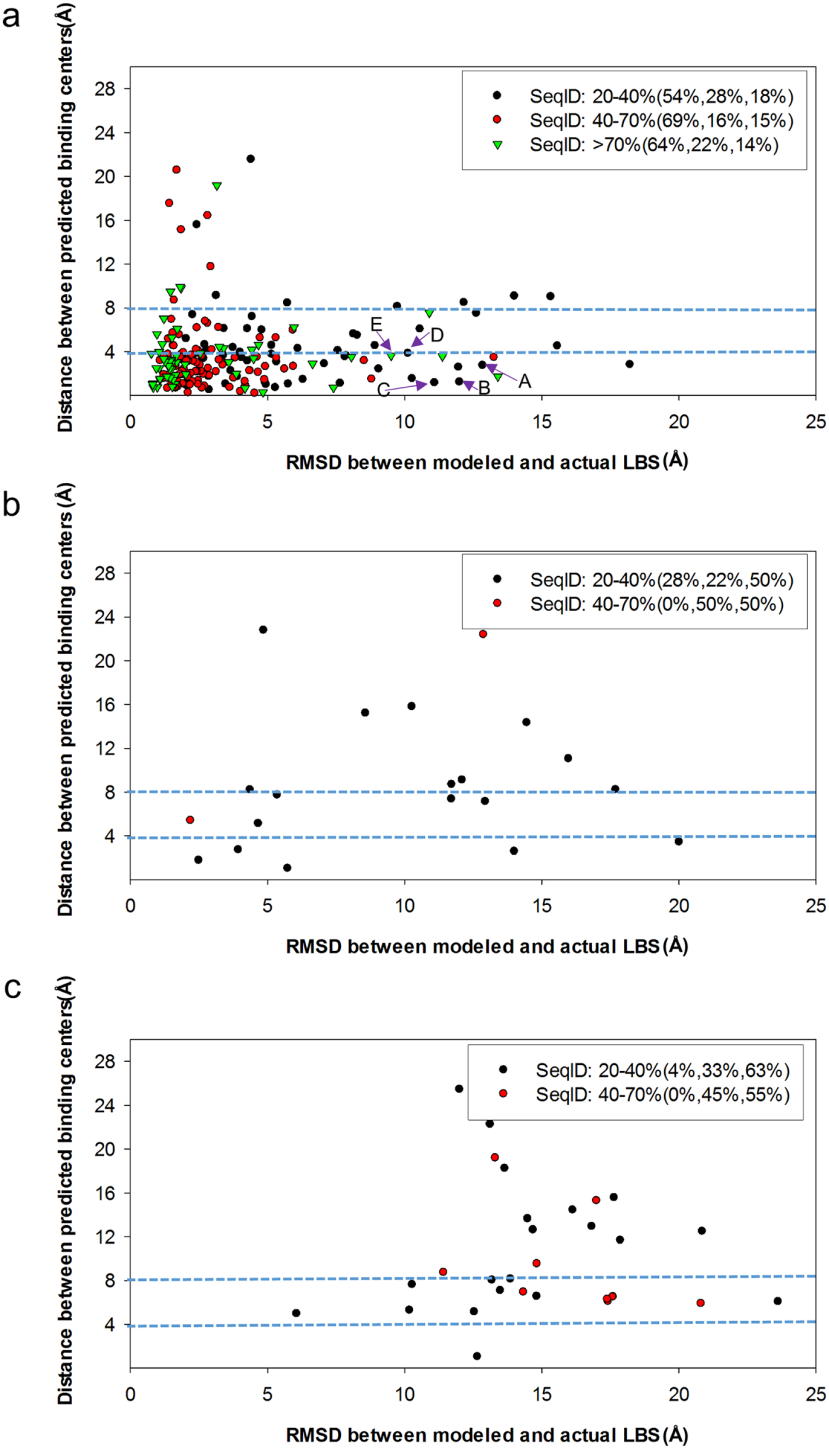
Comparisons of LBS predictions on computationally predicted model structures with those on actual protein structures. **(a)** Data show pairs of actual and modeled structures with template sequence alignment coverage >90%. The y-axis shows the consistency of the predicted LBSs on modeled structures comparing with those on actual structures. The x-axis shows the consistency of the LBS geometry between modeled structures and actual structures. Three percentages shown in the legend after the seqID are the percentages for those data points with y-axis values <4 Å, between 4 Å and 8 Å, and >8 Å respectively. **(b)** Data show pairs of actual and modeled structures with template sequence alignment coverage between 90% and 50%. Other descriptions are the same as in panel (a). **(c)** Data show pairs of actual and modeled structures with template sequence alignment coverage between 50% and 7%. Other descriptions are the same as in panel (a).

The purpose of the tests in this section is to assess the effects of inaccurate protein models on LBS predictions with ISMBLab-LIG. Comparative modeling predicts protein structures with various accuracy level. 305 comparatively modeled structures were predicted with MODELLER (see Methods) for the query sequences from the S48b dataset. Selected templates, and thus associated sequence identities (seqIDs) and alignment coverages (ACs), with diverse evolutionary relevance to the query sequences were used to predict modeled structures, so as to encompass a range of prediction qualities of the modeled structures as shown in detail in S9 Table. The predicted structures are divided into three groups with decreasing structural prediction accuracy: modeled structures with template sequence alignment coverage >90% (Fig 4a); modeled structures with template sequence alignment coverage between 90% and 50% (Fig 4b); model structures with template sequence alignment coverage between 50% and 7% (Fig 4c). The ensemble of the predicted structures with diverse prediction qualities were used to assess the tolerance of structural variations in LBS predictions as shown in Fig 4.

The y-axes of Fig 4a–4c show the consistency of the predicted LBSs on modeled structures comparing with those on actual structures. The consistency is represented by the distance from the geometry center of the predicted top one ligand binding patch (see Methods) on the actual structure to the geometry center of the predicted top one ligand binding patch on the comparatively modeled structure. The pair of the actual structure and the modeled structure were superimposed to optimize the root mean square deviation (RMSD) of the corresponding backbone Cα atoms before calculating the distance between the predicted binding centers.

The x-axes of Fig 4a–4c show the consistency of the LBS geometry between modeled structures and actual structures. The consistency is represented by the RMSD between the ligand-neighboring atoms in the actual structure and the corresponding atoms in the modeled structure. The ligand-neighboring atoms are the atoms within 10 Å to the ligand-binding atoms, which are within 4.5 Å to any of the heavy atom in the corresponding ligand.

Modeled structures with moderate prediction accuracy can be used to predict LBSs with reasonable relevance to the LBSs on the actual structures. Fig 4a shows that when sequence ID between the query sequence and the template > 70% and alignment coverage > 90% (green data points in Fig 4a), the predicted LBSs on the modeled structures are quite consistent with those on the actual structures: 64% of the cases have the predicted binding site centers on modeled and actual structures within 4Å; 86% of the cases have predicted binding site centers within 8Å. As the sequence ID decreases, the structural variation increases, but the predicted binding site centers mostly remain within 8Å (red and black data points in Fig 4a). However, as the quality of the modeled structure deteriorates, the predicted LBSs from the modeled structures are increasingly inconsistent with the predicted LBSs from the actual structures (Fig 4b and 4c).

**Fig 5:**
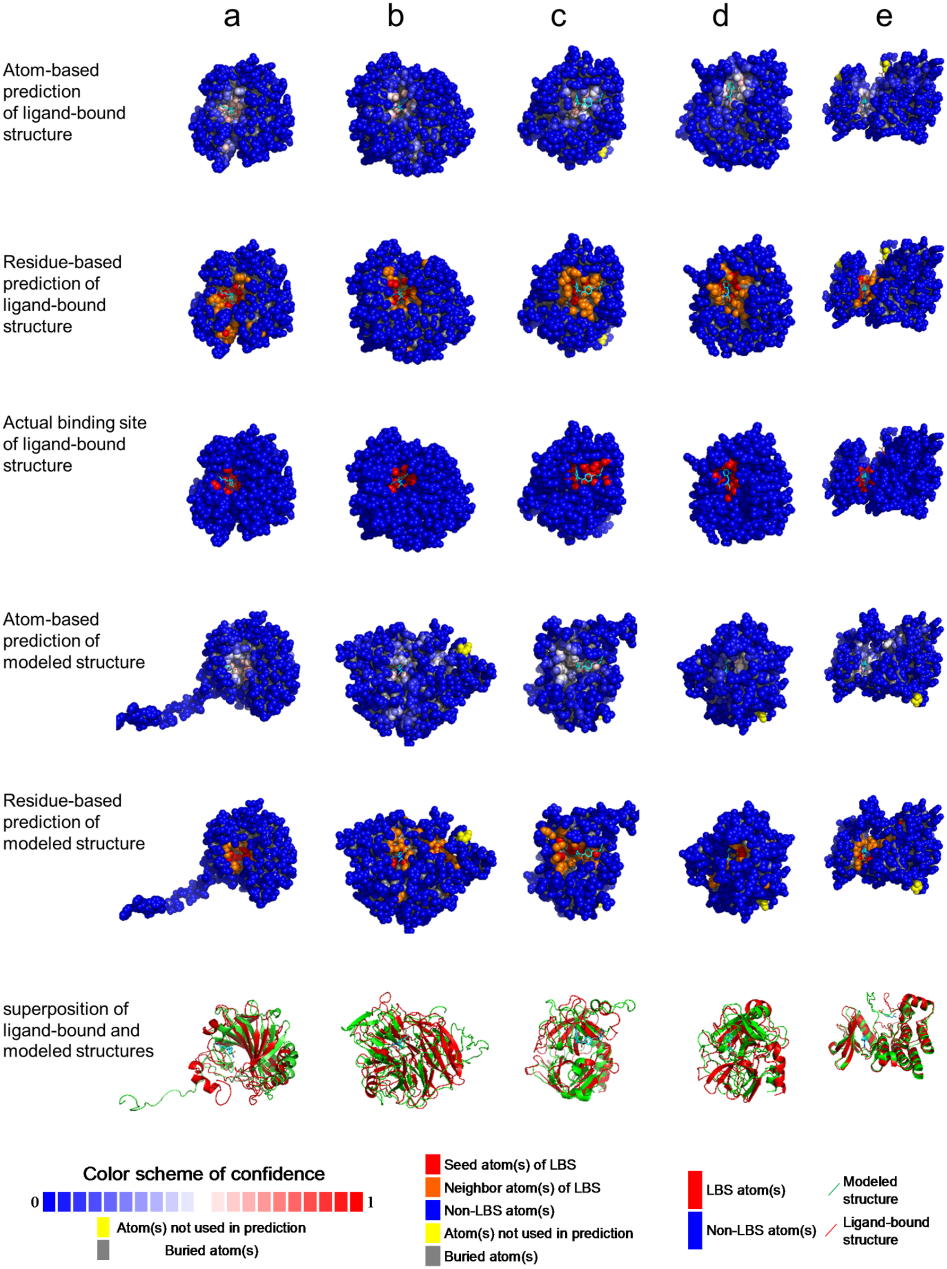
Examples of structural variation tolerance in ligand binding site predictions. The LBS predictions for the pairs of modeled and actual structures for which the data points are marked A~E in Fig 4a are shown here in a~e respectively. The first and 4^th^ row show the atom-based predictions for the actual structures and the corresponding modeled structures respectively. The second and 5^th^ row show the residue-based ligand binding site predictions for the actual structures and the corresponding modeled structures respectively. The third row shows the actual binding sites for ligand-bound structures. The last row shows the ribbon diagrams of the superimposed pairs of the modeled (green) and actual (red) structures. The details of PDB codes for ligand-bound structures and model templates, sequence identities, alignment coverages, RMSDs of ligand-neighboring atoms, distances between predicted binding centers for these five pairs of structures are shown in S6 Table.

Detailed LBS predictions on the actual and modeled structures are compared for a few of the cases with large variations in the ligand binding sites (data points labeled by A~E in Fig 4a) are shown in Fig 5a to 5e respectively. These results indicate that the ISMBLab-LIG predictors are applicable in LBS predictions for predicted protein structures with moderate prediction accuracy.

### Comparison of ISMBLab-LIG and COACH LBS predictions on protein model structures

**Fig 6:**
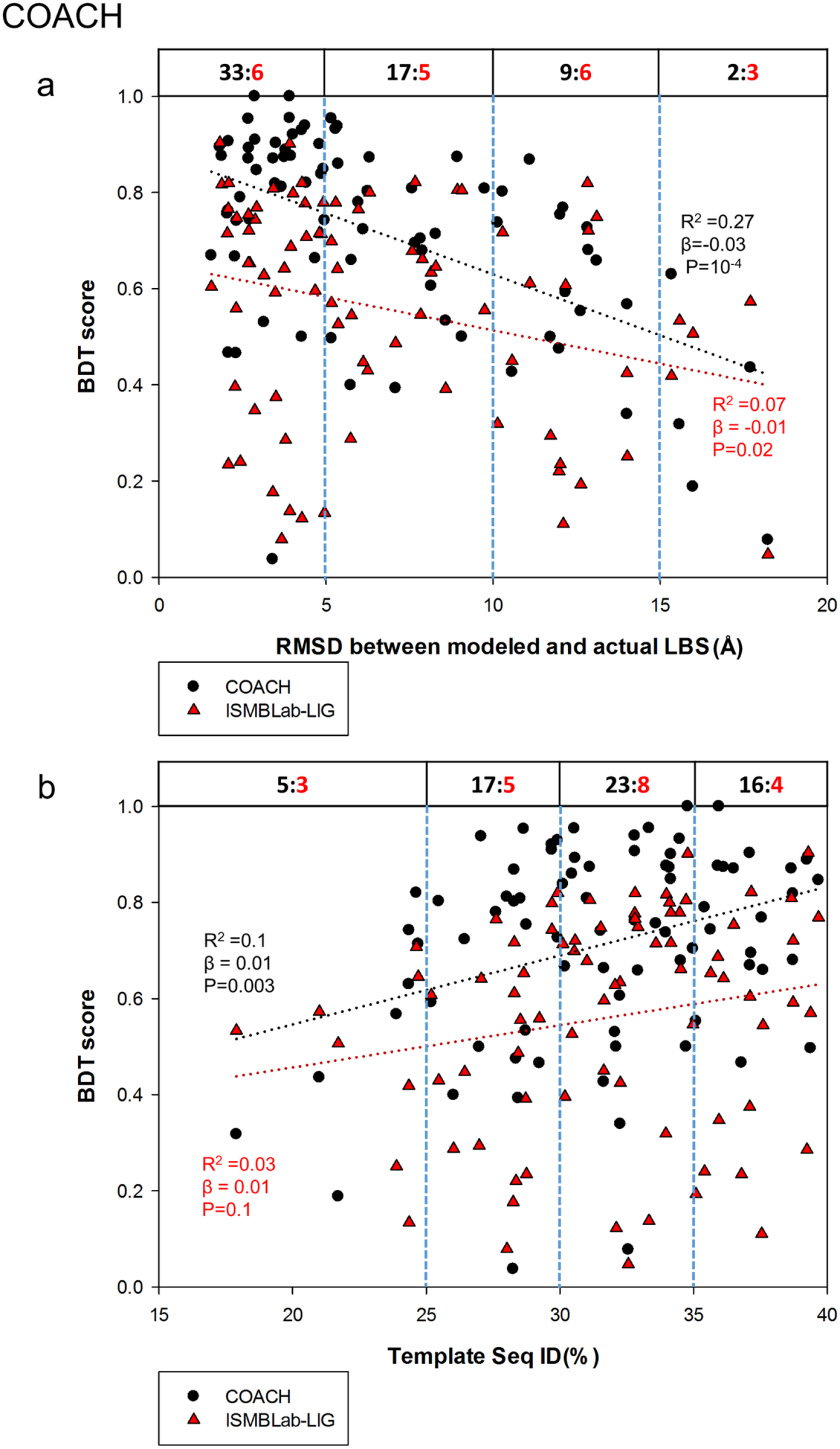
Comparison of ISMBLab-LIG and COACH predictions. (a) Prediction results are compared for 81 model structures built by MODELLER with template sequence alignment coverage > 80% and sequence ID < 40%. The query sequences are from the S48b dataset (see Methods). For each model structure, a pair of BDT scores were calculated based on the LBS predictions with ISMBLab-LIG (red triangles) and COACH (black dot). These BDT scores are plotted against RMSD between modeled and actual LBS (x-axis), for which the definition is the same as the x-axis of Fig 4. The red dotted line and the black dotted line are the linear regression lines for the ISMBLab-LIG data points and COACH data points respectively; the corresponding R^2^, slope (β) and P-value from F-test are calculated by SigmaPlot 12.0 and colored in red for ISMBLab-LIG predictions and black for COACH predictions. For each pair of predictions on the same model structure, a winner was assigned to either ISMBLab-LIG or COACH based on the BDT score. For each subgroup of the data points divided by the vertical blue dashed lines, the black number versus the red number above the data points in the figure panel indicates the number of winners of COACH prediction (in black) versus the number of winners of ISMBLab-LIG prediction (in red). (b) The description is the same as in (a), except that the data points are plotted against the sequence ID % between the query and the template used in MODELLER comparative modeling.

Comparison of the ISMBLab-LIG (a structure-based LBS predictor) with COACH (a consensus LBS predictor combining 5 different algorithms) in prediction accuracy of LBSs on model structures would provide insights into the strength and weakness of different algorithms in terms of tolerance of structural uncertainty of the query target protein without related template of ligand-protein complex in the structural database. Figs 4 and 5 assess the ISMBLab-LIG with the test cases modeled with distantly homologous templates to mimic the situation of the protein models with moderate prediction accuracy and without relevant template of ligand-protein complex in PDB. However, similar experiments are not applicable to assess COACH, which uses sequence information of the query structure, and thus in theory, would nevertheless identify the test cases themselves and sequence-wise related templates in PDB. In fact, all the test query sequences need to have actual ligand-protein complex structures in PDB so as to compare the predicted results with the actual LBSs. Hence, it is not unexpected that COACH would outperform ISMBLab-LIG on the test cases similar to those in Fig 4. Indeed, 81 model structures predicted with MODELLER in default parameters with the sequence ID < 40% and the alignment coverage > 80% between the query (from the S48b dataset) and the template were submitted to the COACH webserver, and the LBS predictions as assessed by the BDT score plotted versus RMSD between the modeled and actual LBSs (Fig 6a) and versus the sequence ID between the query and the template (Fig 6b) are superior to the ISMBLab-LIG predictions, which are also plotted in Fig 6. The performances are compared in Fig 6 in two sets of benchmarks: (1) linear-regression benchmark: the linear regressions of the data points (black dotted line for COACH and red dotted line for ISMBLab-LIG in Fig 6a and 6b) indicate that the performance gap of the two methods narrows only in poorly predicted model structures; (2) pair-winner benchmark: for each subgroup of the data points divided by the vertical blue dashed lines, the black number (number of COACH winners) versus the red number (number of ISMBLab-LIG winner) above the data points in the figure panel (see legend of Fig 6) shows that COACH predictions are more accurate than ISMBLab-LIG predictions in all data point groups.

**Fig 7:**
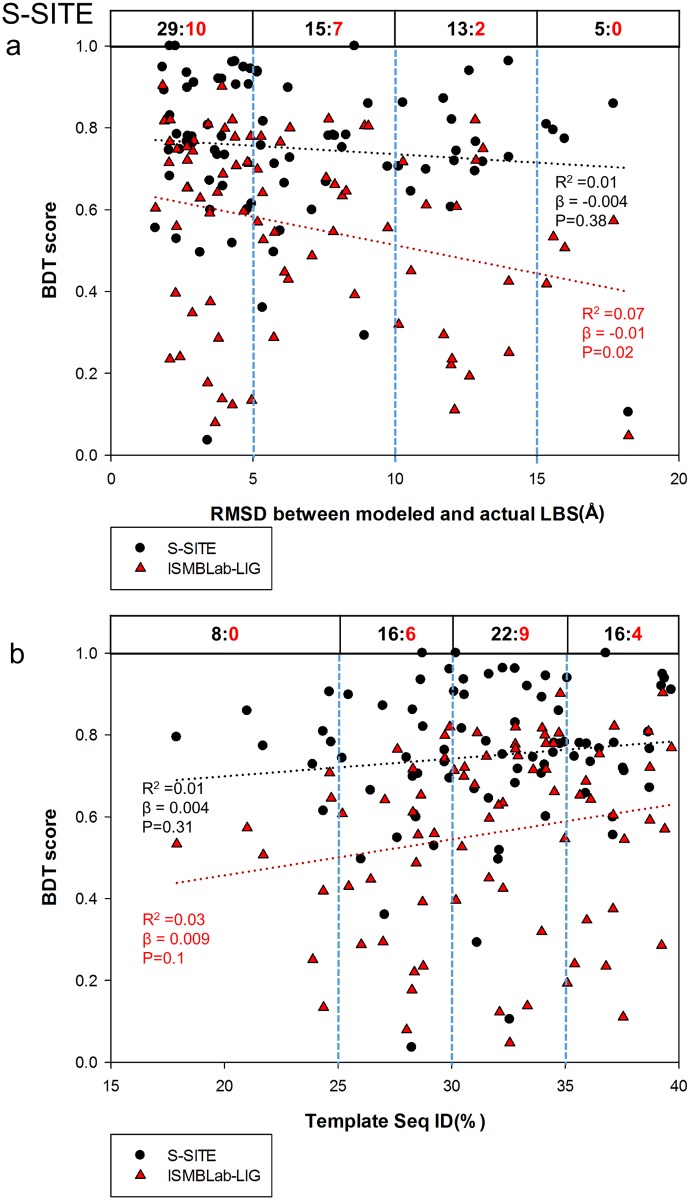
Comparison of ISMBLab-LIG and S-SITE predictions. See description in Fig 6.

**Fig 8:**
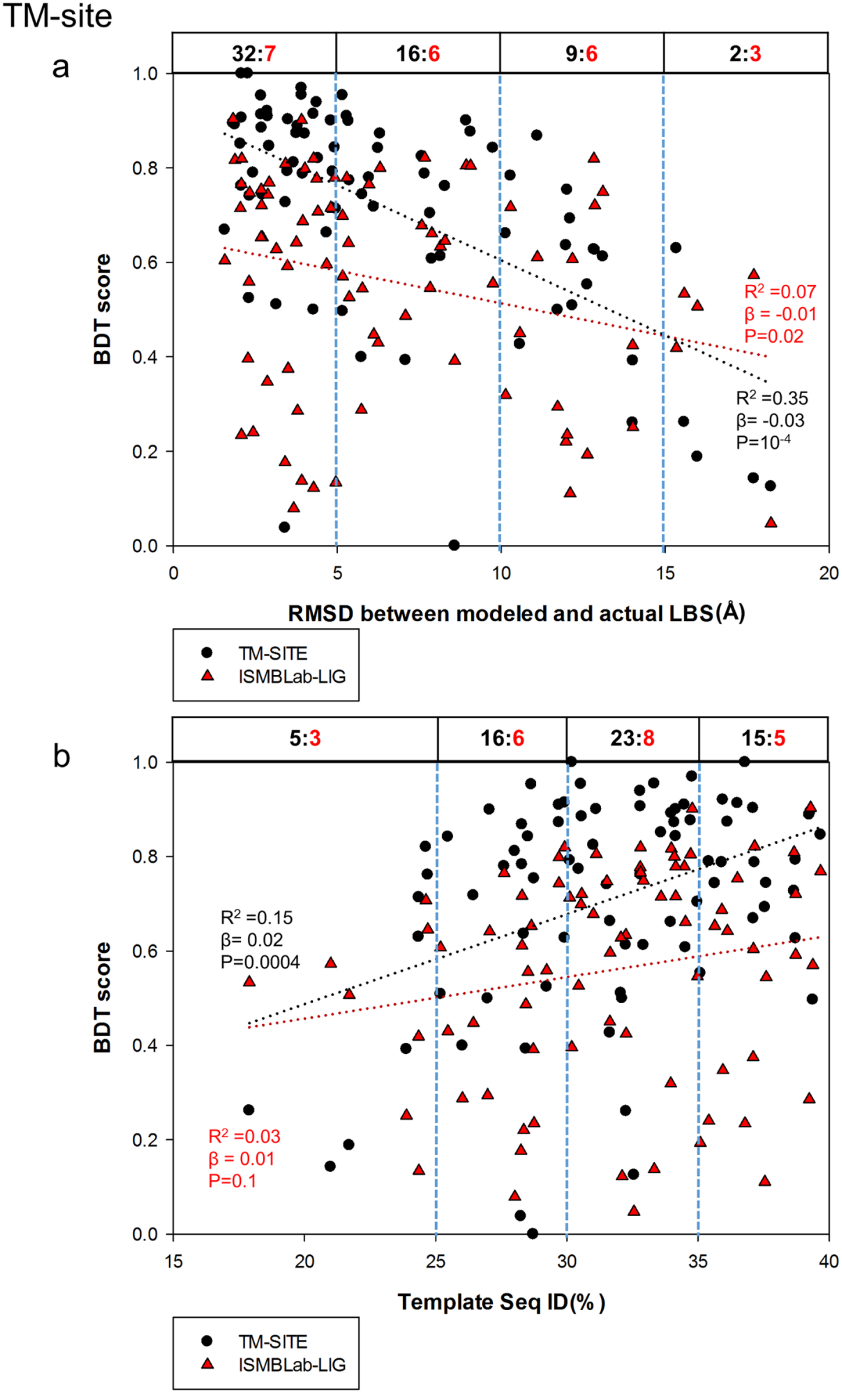
Comparison of ISMBLab-LIG and TM-SITE predictions. See description in Fig 6.

**Fig 9:**
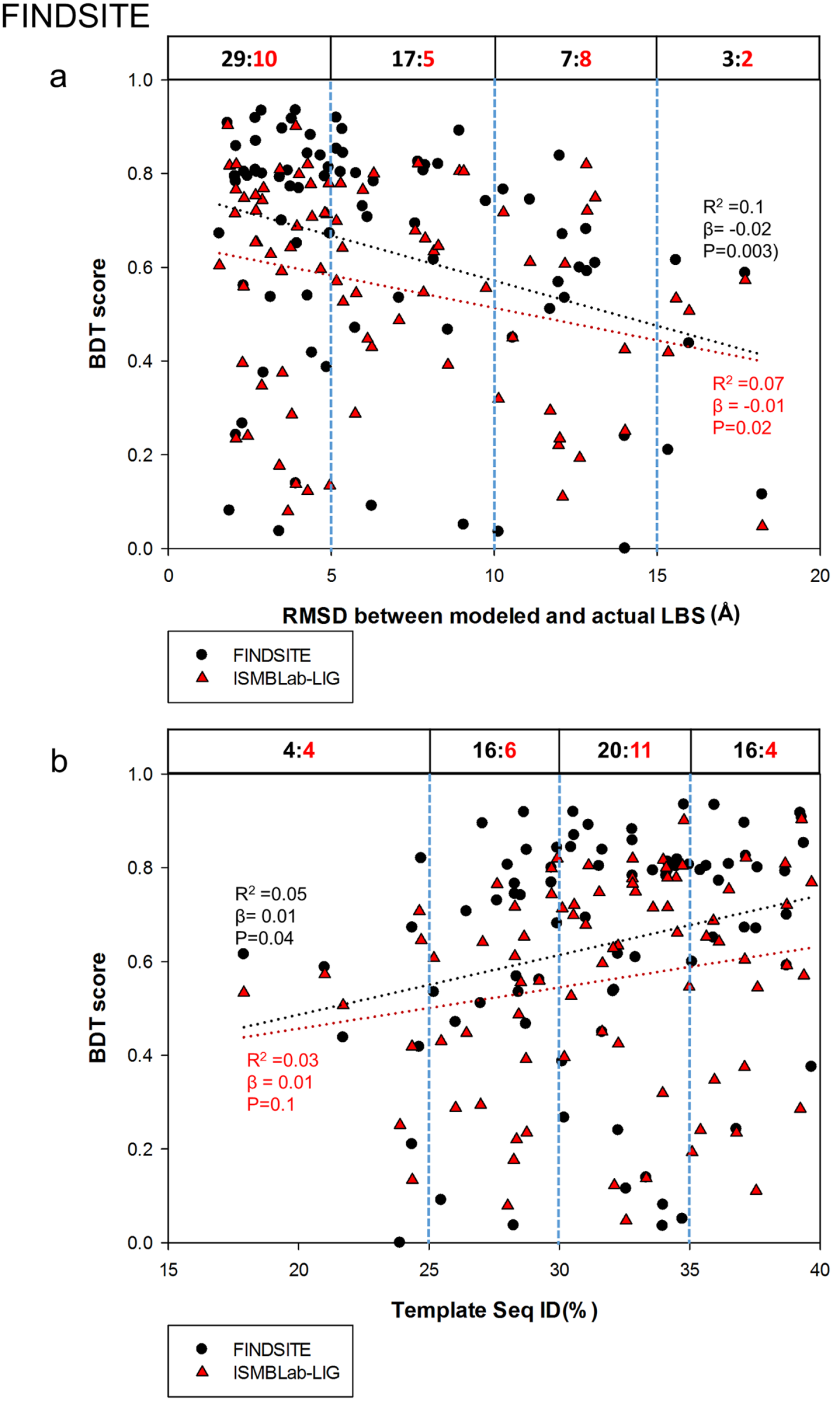
Comparison of ISMBLab-LIG and FINDSITE predictions. See description in Fig 6.

**Fig 10:**
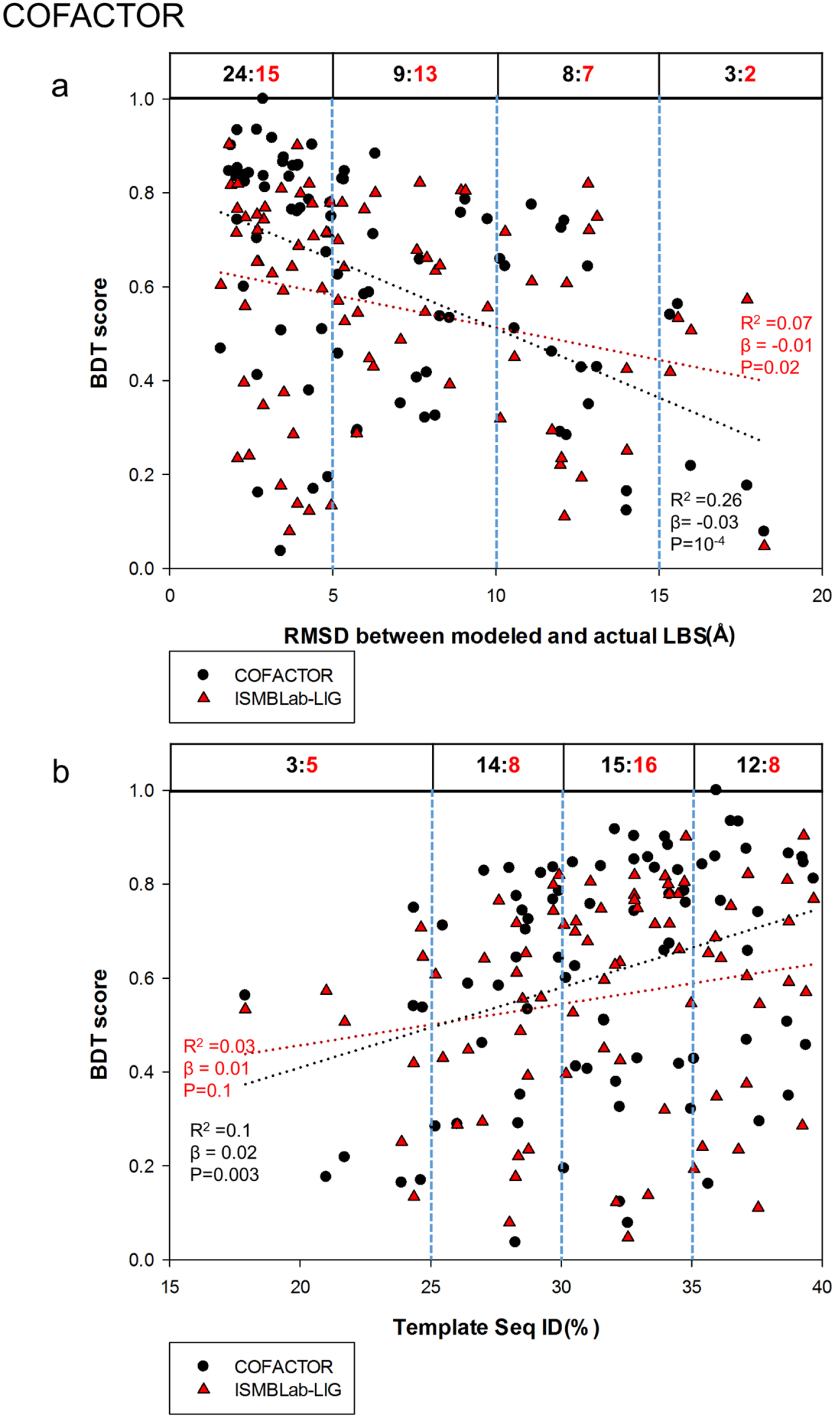
Comparison of ISMBLab-LIG and COFACTOR predictions. See description in Fig 6.

**Fig 11:**
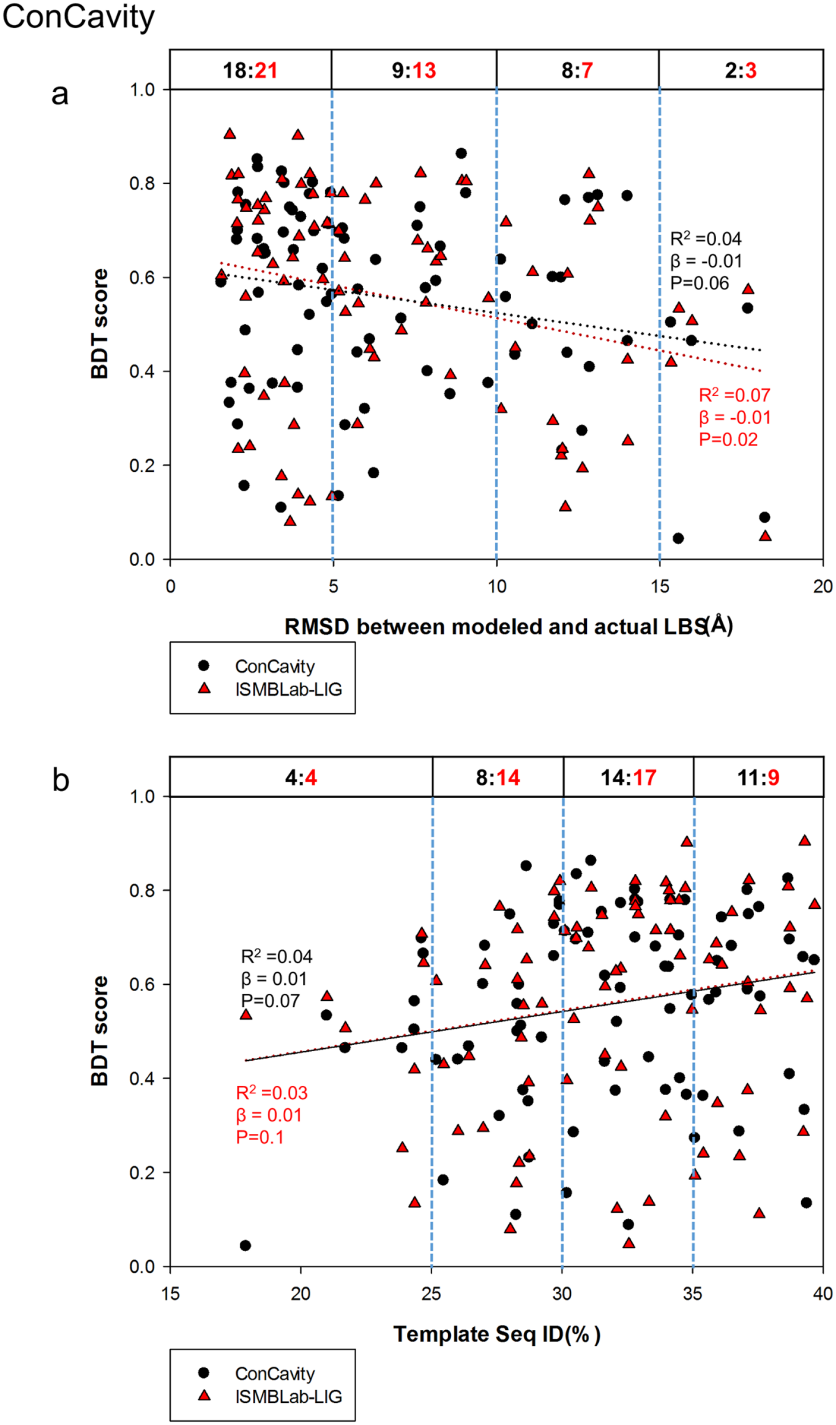
Comparison of ISMBLab-LIG and ConCavity predictions. See description in Fig 6.

The advantage of COACH was gained through sequence information of the test query cases, for which closely related templates (and the queries themselves) can, in theory, be found in PDB. COACH makes use of five prediction outcomes (S-SITE, TM-SITE, FINDSITE, COFACTOR, and ConCavity) with a linear-SVM algorithm for combination weight determination [40]. Hence it is instructive to compare the ISMBLab-LIG predictions with the prediction results from each of the 5 predictors provided also by the COACH webserver (Figs 7–11): S-SITE uses binding-specific sequence-profiles alignment to detect the possible template and predict the LBSs; its scoring method for template match is structure-independent and thus the performance would not be influenced by the quality of modeled structures [40]. Consequently, the S-SITE prediction accuracy is only slightly dependent on the fidelity of the model structures (Fig 7). S-SITE LBS predictions are far superior to those of ISMBLab-LIG as judged by both the linear-regression lines and the pair-winner benchmarks (Fig 7). TM-SITE identifies suitable templates with local and global structure-based alignments; the composite scoring function for evaluating the match between query and template structure includes the sequence conservation information by Jesnson-Shannon divergence score (JSD) from multiple sequence alignments of query sequence with other homologous sequences [10]. FINDSITE first uses the threading method for detecting structural template with ligand and then aligns these templates with query structure for LBS prediction [24]. Both algorithms use sequence and structural information to search for related template ligand-protein complexes in PDB, and hence expectedly, are also superior in prediction performance comparing with ISMBLab-LIG (Figs 8 and 9, respectively). COFACTOR uses global structural search for template candidates, followed by local LBS identification through local query motif superimpositions onto the known functional site residues of the template proteins. The sequence conservation information from JSD score is used to identify the evolutionary conserved residues for generating the local query motifs but not directly used for scoring of template candidates [25]. Thus, the quality of modeled structures has more impact on the prediction accuracy for COFACTOR (Fig 10). The ISMBLab-LIG algorithm is superior to COFACTOR in LBS predictions for the predicted protein structure models with less fidelity (Fig 10), a situation where COFACTOR cannot find relevant templates in PDB. ConCavity identifies the surface cavity with evolutionary sequence conservation information from JSD score; the cavity or pocket search is mainly determined by the input structure conformation [43]. The ConCavity LBS predictions are mostly inferior to those of ISMBLab-LIG by the two sets of benchmarks (Fig 11), likely because the LBS geometry is part of the attributes used in the ISMBLab-LIG machine learning algorithm. In summary, the advantage of the consensus prediction implemented in COACH over ISMBLab-LIG is likely due to the usage of the sequence information of the test cases, for which the actual protein-ligand complex structures are stored in PDB and are accessible to COACH webserver through sequence search; ISMBLab-LIG could be more applicable for LBS predictions with model structures, particularly when homologous template ligand-protein complex for the query protein sequence cannot be found in the protein structural database with either sequence-based or structure-based search methods.

**Fig 12:**
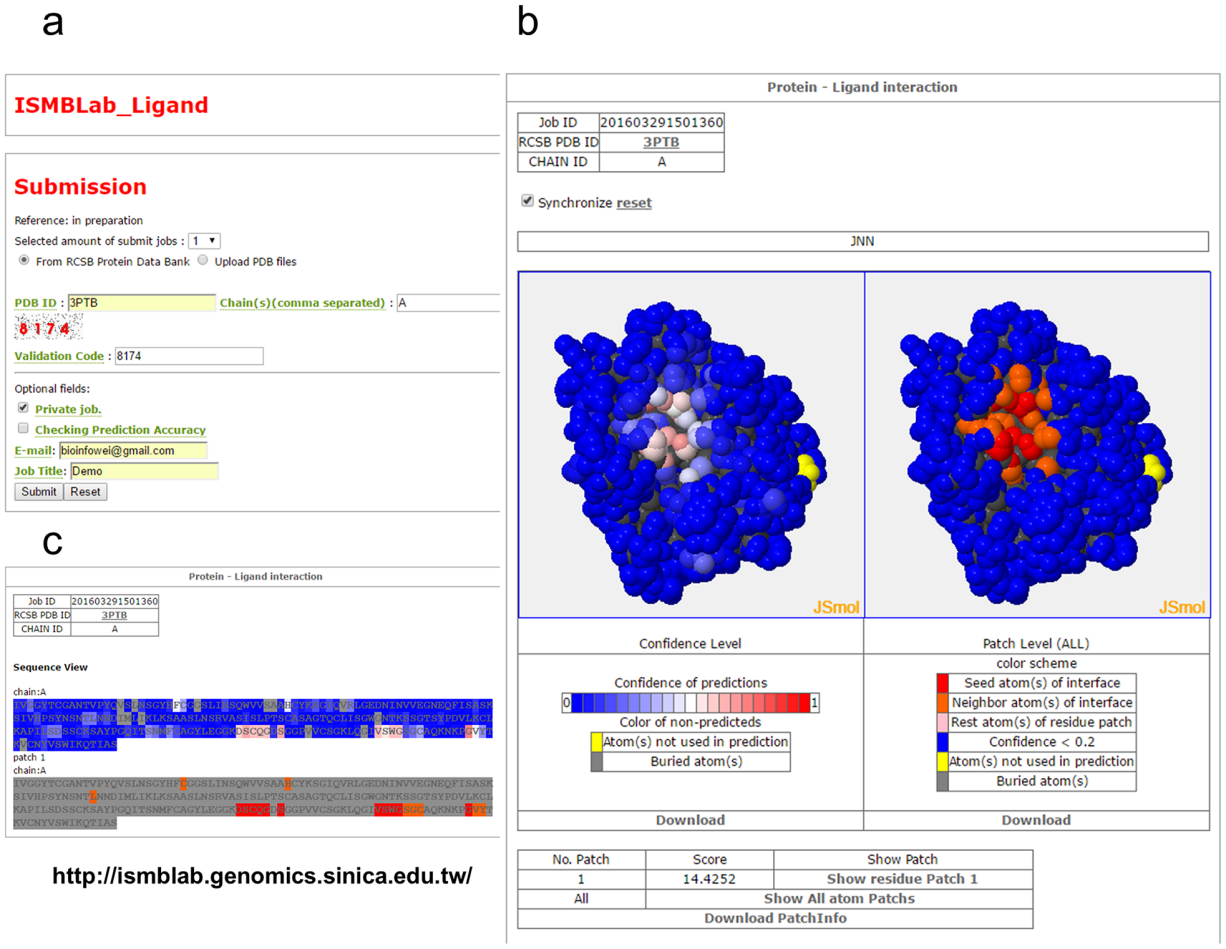
Input and output of the ISMBLab-LIG web server. **(a)** User can upload structures in PDB format or assign PDB ID with chain identifier for prediction jobs. **(b)** Two types of prediction results, atom-based confidence level (left panel) and residue-based predicting patches (right panel), are displayed separately with Jmol for interactive 3D structural view. The same color scheme described in Fig 3 is also applied here for confidence level and predicted patches respectively. Both confidence level and patch prediction results can be downloaded as PDB format file where B-factor field contains these information. **(c)** The prediction results are also shown in sequence-based view. The color scheme is the same as in panel (b).

ISMBLab-LIG can be accessed through the ISMBLab-LIG web server (Fig 12).

## Discussion

Predicting ligand binding sites on protein structures, which are obtained either from experimental or computational methods, is a useful first step in functional annotation or structure-based drug design for the protein structures. The prediction capabilities of LBS prediction methods based on evolutionary information or ligand binding pocket geometry have been successful to an extent, and the template-based methods have better performance when known template structures are available for inferring tentative ligand binding sites on the query protein. However, the template-based prediction methods could have difficulties to predict tentative LBSs on experimental structures or computationally predicted models when relevant template structures are not available for the query structures. In this work, the structure-based machine learning algorithm ISMBLab-LIG was developed to predict LBSs on protein surfaces with input attributes derived from the three-dimensional probability density maps of interacting atoms on the query protein surfaces. Similar to template-based prediction algorithms, the patterns of LBSs from known structures are learned to infer tentative LBSs on query protein structures. But unlike template structure-matching in the template-based methods, the machine learning predictors, which use interacting atom distributions reconstructed on the query protein surfaces as inputs, are relatively insensitive to local conformational variations. Essentially, the machine learning predictors identify LBSs by mostly recognizing concave pockets on protein surfaces with higher distribution density of the ligand atoms.

The prediction accuracies of the ISMBLab-LIG predictors are comparable to those of the best LBS predictors on several well-established testing datasets. With BDT score as the benchmark, the ISMBLab-LIG predictions were only inferior to the consensus prediction algorithm COACH on 19 CAMEO-LB protein targets released recently. Although the COACH predictions outperformed those of ISMBLab-LIG on the test cases with the model structures predicted with moderate accuracy, the advantage of the consensus prediction implemented in COACH over ISMBLab-LIG is likely due to the usage of the test cases’ sequence information, which might not be informative in situations where homologous template ligand-protein complex for the query protein sequence cannot be found in the protein structural database with either sequence-based or structure-based search methods. It has been estimated that about three quarters of human proteome cannot be inferred in ligand binding information from protein-ligand complexes in PDB [1]. In such situation, ISMBLab-LIG could have unique usefulness for LBS predictions with model structures of structure prediction uncertainties. To summarize, the method is particularly useful for predicting LBSs not only on experimental protein structures without known LBS templates in the database, but also on computationally predicted model protein structures with structural uncertainties in the tentative ligand binding sites and without sequence-wise homologous template ligand-protein complex structures in PDB.

## Methods

ISMBLab-LIG predicts LBSs on a protein structure by identifying ligand-binding atoms on the protein surface according to the predicted ligand-binding confidence level for each of the protein surface atoms. The general principles of the prediction method have been published previously [37, 38].

### Database for non-covalent atomistic interactions

Details of the methodology is documented in S1 Text. In brief, atomistic contact interactions in proteins of known structures were organized into a database containing non-covalent atomistic interaction information for atom pairs involving in protein-protein, protein-ligand and protein-water interactions: The atomistic contact interactions among protein atoms were derived from 9468 non-redundant protein structures [44]. Protein-water interaction database was constructed from 915 non-redundant high resolution protein structures [45].

Unique to this work is the construction of the database for organic ligand atoms interacting with proteins. The interacting ligand atom distribution database was derived from 17023 known protein-ligand complex structures (S17023 dataset) released before Aug/2014. These complex structures were obtained from PDB web site: http://ligand-expo.rcsb.org/dictionaries/cc-to-pdb.tdd. 30% sequence identity threshold was used to remove structure redundancy among structures binding to the same ligand. In addition, structures that satisfy at least one of the following criteria were further removed from the dataset: non X-ray method; resolution higher than 3 Å; ATOM record (PDB format) containing DNA or RNA molecule; only C alpha atoms; covalent link between ligand and protein; the number of ligand atoms smaller than 10 and the number of interacting atoms smaller than 15. The formal name and description of atom type for ligand molecules in Table 1 were adopted from Sybyl atom type model [46]. OpenBabel package [47] was used to define the ligand atom types, as shown in Table 1 with ID number from 32 to 53. The constructions of the database for the distributions of interacting ligand atom types and interacting protein atom types are described in details in S1 Text [37, 38].

### Constructing probability density maps (PDMs) of non-covalent interacting atoms on protein surfaces

Details of the methodology is documented in S1 Text. In brief, a probability density map (PDM) for an interacting atom type describes the three-dimensional distribution of likelihood for the type of non-covalent interacting atom to appear around protein surface atoms. For each atom in the query protein structure, the atom type, amino acid type of the parent residue and the conformational type of the parent amino acid were combined to retrieve interacting atoms in the database described in previous section. The distributions of the interacting atoms were normalized and mapped to the protein surface to construct the PDMs [36].

### Input of the ISMBLab-LIG predictors

One artificial neural network (ANN) machine-learning model (ANN_BAGGING) (S1 Text) [37, 38] was trained for each of the 30 protein atom types. The input for each of the 30 ANN models contains 54 attributes: 53 attributes encode the ligand-binding properties for each of the protein surface atoms; one additional attribute describes the geometry around each protein surface atom—the fraction of space not occupied by the van der Waals volume of the protein in the 10 Å sphere centered at the protein atom [37, 38]. Each of the 53 ligand-binding property attributes was extracted from a corresponding three-dimensional probability density map (PDM) that describes the spatial distribution of one of the 53 interacting atom types around the protein surface atom; the interacting atom types are listed with the ID number from 1 to 53 in Table 1. 31 PDMs for each protein surface atom were constructed using a database containing the distributions of interacting protein atom types (ID number from 1 to 30 shown in Table 1) and water oxygen (ID number of 31 shown in Table 1) around protein atoms from known protein structures [37, 38]; 22 PDMs were constructed using the database containing the distributions of interacting ligand atom types (ID number from 32 to 53 shown in Table 1) around protein atoms (see above). The derivation of the attribute values from PDMs has been published and the details are documented in S1 Text.

### Output of the ISMBLab-LIG LBS predictions at residue-based level

The outputs of the ANN models are normalized ligand-binding prediction confidence levels ranging from 0 to 1, allowing the prediction results of the protein surface atoms from the 30 ANN models to be used on a leveled ground to synthesize the final ligand binding patches based on a threshold of the prediction confidence level (S1 Text) [37, 38]: Seed patches were defined as those surface atoms showing the prediction confidence level greater than 50%. All neighboring surface atoms within 5 Å radii of these seeds and with prediction confidence level greater than 10% were included as predicted ligand binding site. If any two seeds showing the pairwise distance smaller than10 Å, the subset ligand patches were merged as one ligand binding patch. The predicted atom-based patch containing the highest score from the summation of prediction confidence level by all atoms included was denoted as the top one predicted site. To facilitate comparison of this work with previous methods predicting ligand binding sites at the residue-based level, a heuristic procedure was used to transform the atom-based binding site predictions into binding site predictions at the residue-based level: only the residues with more than 30% of the surface atoms (SASA>0) included in the atom-based binding patch were considered as positive residues of the residue-based patch. The threshold parameters for the atom-based and residue-based prediction heuristics were optimized to maximize prediction accuracies (MCCs) with the validation sets in the 10-fold cross validation (S1 Text).

### Definition of actual LBSs at residue-based level

The actual ligand binding sites at the residue-based level for proteins with known ligands were defined by the residues, each of which has more than 30% of the surface atoms (SASA > 0 in the absence of ligand) on the residue within 4.5 Å to any atom of the ligand. These definitions enabled the comparison of prediction results with actual binding sites at the residue-based level. The percentage parameter was optimized for residue-based prediction accuracy with the validation sets in the 10-fold cross validation.

### Training and testing datasets

S5010 dataset was used for 10-fold cross validations in training and testing of the ISMBLab-LIG predictors. The S5010 dataset contains a subset of 5010 protein-ligand complex structures from the S17023 dataset with pairwise sequence identity less than 90%. In addition, all the protein structures in S5010 contain only one polypeptide chain.

The following test sets were used to benchmark the ISMBLab-LIG predictors. None of the test set structures were included in the training set S5010.

1. The S48-bound (S48b) and S48-unbound (S48ub) test sets were originally collected in the work on LIGSITEcsc [11]; the dataset contains the cases from the works on Q-SiteFinder [48](35 cases) and PASS [49] (19 cases). The dataset contained 48 proteins bound with ligand (S48b) and the same protein structures determined in the absence of the corresponding ligands (S48ub).
2. The S210 test set was collected in the work on LIGSITEcsc [11] and was derived from 485 cases in PLD v 1.3 database (Protein Ligand database) [50] after removing redundant structures.
3. The S198 test set was original created in the work on MPK2 [28] from the DrugPort website [51] and contains 198 protein-drug complexes. The PDB lists of the testing sets were downloaded from metaPocket 2.0 web site [28]:http://projects.biotec.tu-dresden.de/metapocket/benchmark.php
4. The S523 test set was originally collected in the work on COFACTOR [21] which contains 382 natural ligand-protein complexes and 200 drug-protein complexes. The original PDB list was downloaded by COFACTOR benchmark site: http://zhanglab.ccmb.med.umich.edu/COFACTOR/benchmark/COFACTOR_testing_results.txt. 41 PDBs were removed from the original list due to non-availability of ligand name in the current PDB web site. 18 cases with the redundant PDB ID and ligand name were also removed from the original list.

### Benchmarking prediction performances

The machining learning performance of the ANN_BAGGING models was benchmarked by accuracy (Acc), precision (Pre), sensitivity (Sen), specificity (Spe), F-score (Fsc) and Matthews correlation coefficient (MCC).

$$\text{Acc }=\frac{TP+TN}{TP+TN+FP+FN}\times 100$$ (1)

$$\text{Pre}=\frac{TP}{TP+FP}\times 100$$ (2)

$$\text{Sen }=\frac{TP}{TP+FN}\times 100$$ (3)

$$\text{Spe }=\frac{TN}{TN+FP}\times 100$$ (4)

$$\text{Fsc}=\frac{\text{2}\times \text{Pre}\times \text{Sen}}{\text{Pre}\;+\;\text{Sen}}$$ (5)

$$\text{MCC }=\frac{TP\times TN-FP\times FN}{\sqrt{\left(TP+FP)(TP+FN)(TN+FP)(TN+FN\right)}}$$ (6)

where TP is the number of true positives; TN the number of true negatives; FP the number of false positives; and FN the number of false negatives (S1 Text).

The BDT (Binding-site Distance Test) score [42] is defined in Eq 7:

$$\text{BDT}=\frac{\sum_{i=1}^{N_{p}}\text{max}{\left(S_{ij}\right)}_{}}{\text{max}\left(N_{p},N_{o}\right)}$$ (7)

and,

$${\text{S}}_{\text{ij}}=\frac{1}{1+{\left(\frac{d_{ij}}{d_{o}}\right)}^{2}}$$ (8)

where d_*ij*_ is the Euclidean distance between C-alpha atoms of a predicted residue *i* and a LBS residue *j*; d_*0*_ is a distance threshold. In this work, we followed the CAMEO-LB setting of d_*0*_ = 5 Å. N_*p*_ is the number of predicted residues and N_*o*_ is the number of LBS residues. A java package downloaded from the web site: http://www.reading.ac.uk/bioinf/downloads is used for the BDT calculation.

### Evaluation of the LBS prediction performance with comparatively modeled structures

The sequences of S48b data set were used for identifying all possible similar proteins within specific sequence identity threshold by NCBI PSI-blast search using the default parameter [52]. For each query sequence, all identified homologous proteins were classified into 15 groups with descending sequence identities between these proteins and the query sequence. For each of the groups, the protein showing the highest sequence identity with the query sequence was selected as template for modeling the query sequence. Each modeled structure was created based on a single selected template by MODELLER software using the default parameter [53]. The templates and associated data are shown in S6 Table.

### Web Site

The prediction request can be submitted to this webserver: http://ismblab.genomics.sinica.edu.tw/. (Fig 12). All the benchmark results can be accessed in interactive graphic presentations from the same web address above.

## Supporting Information

S1 Fig Comparison of LBS predictions with actual LBSs for targets from CAMEO-LB by ISMBLab-LIG with COACH, RaptorX and COFACTOR. Panels (a) to (v) compare the prediction results of 22 proteins from CAMEO-LB with actual LBSs. In each panel, the first structure from the left shows the actual LBS residues colored in green with ligand in red. Each of the actual LBS residue contains at least one heavy atom within the distance of the sum of Van der Waals radii plus the tolerance distance (0.5 Å) to any ligand heavy atom. The PDB ID and chain ID for the target protein are shown under the target protein structure. The second to the fifth structures from left show the top 1 predicted LBS residues (cyan) by ISMBLab-LIG, COFACTOR, COACH and RaptorX respectively. The BDT score and MCC by the corresponding predictor are shown under each structure. The highest BDT score or MCC among the four predictors are highlighted in red. The PDB ID and chain ID of PDBhit, which is the representative protein-ligand complex structure identified in COACH result page, are also shown under the COACH prediction result. The number in the bracket is the sequence ID% between the target protein and the representative protein.
(PDF)

S1 Table ANN_BAGGING prediction accuracy benchmarks on the independent test set S48b. The PDB ID, chain ID, and ligand name (columns 1~3) are downloaded from PDB; the prediction performances shown in columns 4~13 are defined in Eqs 5–10 of S1 Text; column 14 shows the number of LBS predicted for the corresponding protein structure (see Methods in main text); column 15 shows the number of the top one predicted LBS (see Methods in main text) for which the geometry center is within 4Å to the corresponding ligand.
(DOCX)

S2 Table ANN_BAGGING prediction accuracy benchmarks on the independent test set S48ub. The PDB ID, chain ID, and ligand name (columns 1~3) are downloaded from PDB; the prediction performances shown in columns 4~13 are defined in Eqs 5–10 of S1 Text; column 14 shows the number of LBS predicted for the corresponding protein structure (see Methods in main text); column 15 shows the number of the top one predicted LBS (see Methods in main text) for which the geometry center is within 4Å to the corresponding ligand.
(DOCX)

S3 Table ANN_BAGGING prediction accuracy benchmarks on the independent test set S210. The PDB ID, chain ID, and ligand name (columns 1~3) are downloaded from PDB; the prediction performances shown in columns 4~13 are defined in Eqs 5–10 of S1 Text; column 14 shows the number of LBS predicted for the corresponding protein structure (see Methods in main text); column 15 shows the number of the top one predicted LBS (see Methods in main text) for which the geometry center is within 4Å to the corresponding ligand.
(DOCX)

S4 Table ANN_BAGGING prediction accuracy benchmarks on the independent test set S198. The PDB ID, chain ID, and ligand name (columns 1~3) are downloaded from PDB; the prediction performances shown in columns 4~13 are defined in Eqs 5–10 of S1 Text; column 14 shows the number of LBS predicted for the corresponding protein structure (see Methods in main text); column 15 shows the number of the top one predicted LBS (see Methods in main text) for which the geometry center is within 4Å to the corresponding ligand.
(DOCX)

S5 Table ANN_BAGGING prediction accuracy benchmarks on the independent test set S523. The PDB ID, chain ID, and ligand name (columns 1~3) are downloaded from PDB; the prediction performances shown in columns 4~13 are defined in Eqs 5–10 of S1 Text; column 14 shows the number of actual LBSs for the corresponding complex structure; column 15 shows the number of LBS predicted for the corresponding protein structure (see Methods in main text);.
(DOCX)

S6 Table Comparison of the top 1 predicted success rates of ISMBLab-LIG with those of various ligand binding site predictors on the S48 bound/unbound dataset. (DOCX)

S7 Table Comparisons of the top 1 prediction success rates of ISMBLab-LIG with those of various ligand binding site prediction methods on the S210 dataset. (DOCX)

S8 Table Comparisons of the top 1 prediction success rates of ISMBLab-LIG with those of various ligand binding site prediction methods on the S198 dataset. (DOCX)

S9 Table Details of model template and distances of predicted binding centers between ligand-bound structures and modeled structures. Columns 1 and 2 show the PDB code name and chain name respectively for the query protein sequence to be comparatively modeled with MODELLER. Column 3 shows the PDB code name of the template structure used in the comparative modeling. Columns 4 and 5 show the sequence ID (%) and alignment coverage fraction between the query sequence and the template structure. Column 6 shows the root mean square deviation (RMSD) between the ligand-neighboring atoms in the actual structure (shown in column 1) and the corresponding atoms in the modeled structure. The ligand-neighboring atoms are the atoms within 10 Å to the ligand-binding atoms, which are within 4.5 Å to any of the heavy atom in the corresponding ligand. Column 7 shows the distance from the geometry center of the predicted top one ligand binding patch (see Methods in the man text) on the actual structure (column 1) to the geometry center of the predicted top one ligand binding patch on the comparatively modeled structure based on the template structure (column3). The actual structure and the modeled structure were superimposed to optimize the RMSD of corresponding backbone Cα before calculating the distance between the predicted binding center. Column 8 indicates the examples (A)~(E) shown in Fig 5 of the main text.
(DOCX)

S10 Table Prediction results of the CAMEO-LB cases by RaptorX, COACH, COFACTOR and ISMBLab-LIG. Columns 1 and 2 from the left show the PDB code name and chain name respectively for the query protein sequence comparatively modeled with I-TASSAR package. Column 3 shows the residue numbers of actual LBS residues, each of which contains at least one heavy atom within the distance of the sum of Van der Waals radii plus the tolerance distance (0.5 Å) to any ligand heavy atom. Columns 4, 5, 6 and 7 show the residue numbers of predicted LBS residues by ISMBLab-LIG, COFACTOR, COACH and RaptorX respectively.
(DOCX)

S1 Text Supplemental Methods. (DOCX)
