# Supplementary material for: Predicting Ligand Binding Sites on Protein Surfaces by 3-Dimensional Probability Density Distributions of Interacting Atoms
Source: PLoS One. 2016 Aug 11;11(8):e0160315. doi: 10.1371/journal.pone.0160315 (PMC4981321; doi:10.1371/journal.pone.0160315)
Supplement: S1 Fig — Panels (a) to (v) compare the prediction results of 22 proteins from CAMEO-LB with actual LBSs. In each panel, the first structure from the left shows the actual LBS residues colored in green with ligand in red. Each of the actual LBS residue contains at least one heavy atom within the distance of the sum of Van der Waals radii plus the tolerance distance (0.5 Å) to any ligand heavy atom. The PDB ID and chain ID for the target protein are shown under the target protein structure. The second to the fifth structures from left show the top 1 predicted LBS residues (cyan) by ISMBLab-LIG, COFACTOR, COACH and RaptorX respectively. The BDT score and MCC by the corresponding predictor are shown under each structure. The highest BDT score or MCC among the four predictors are highlighted in red. The PDB ID and chain ID of PDBhit, which is the representative protein-ligand complex structure identified in COACH result page, are also shown under the COACH prediction result. The number in the bracket is the sequence ID% between the target protein and the representative protein. (PDF) [file pone.0160315.s001.pdf]

S1 Fig

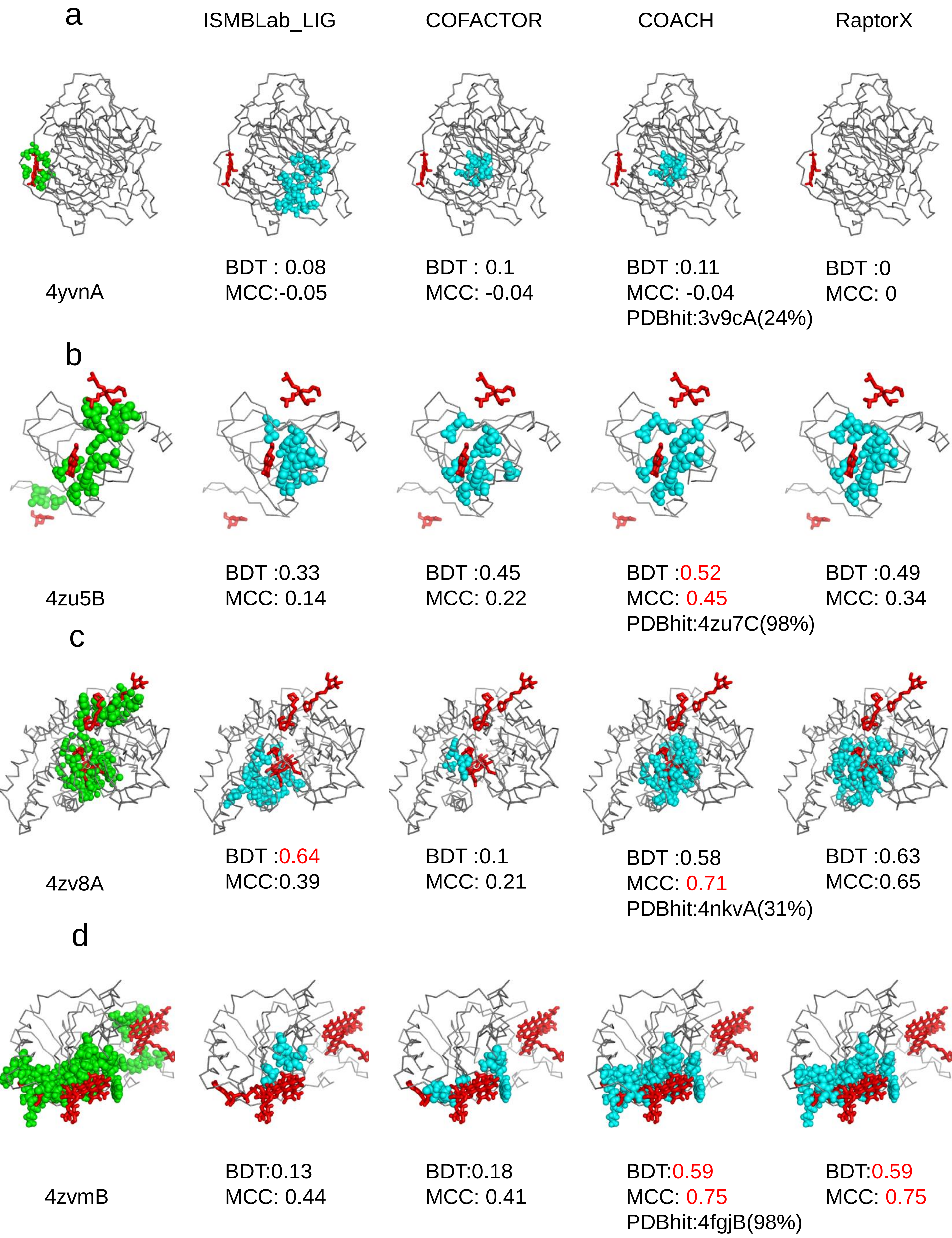
a
ISMBLab_LIG
COFACTOR
COACH
RaptorX
4yvnA
BDT : 0.08
MCC:-0.05
BDT : 0.1
MCC: -0.04
BDT :0.11
MCC: -0.04
PDBhit:3v9cA(24%)
BDT :0
MCC: 0
b
4zu5B
BDT :0.33
MCC: 0.14
BDT :0.45
MCC: 0.22
BDT :0.52
MCC: 0.45
PDBhit:4zu7C(98%)
BDT :0.49
MCC: 0.34
c
4zv8A
BDT :0.64
MCC:0.39
BDT :0.1
MCC: 0.21
BDT :0.58
MCC: 0.71
PDBhit:4nkvA(31%)
BDT :0.63
MCC:0.65
d
4zvmB
BDT:0.13
MCC: 0.44
BDT:0.18
MCC: 0.41
BDT:0.59
MCC: 0.75
PDBhit:4fgjB(98%)
BDT:0.59
MCC: 0.75

S1 Fig

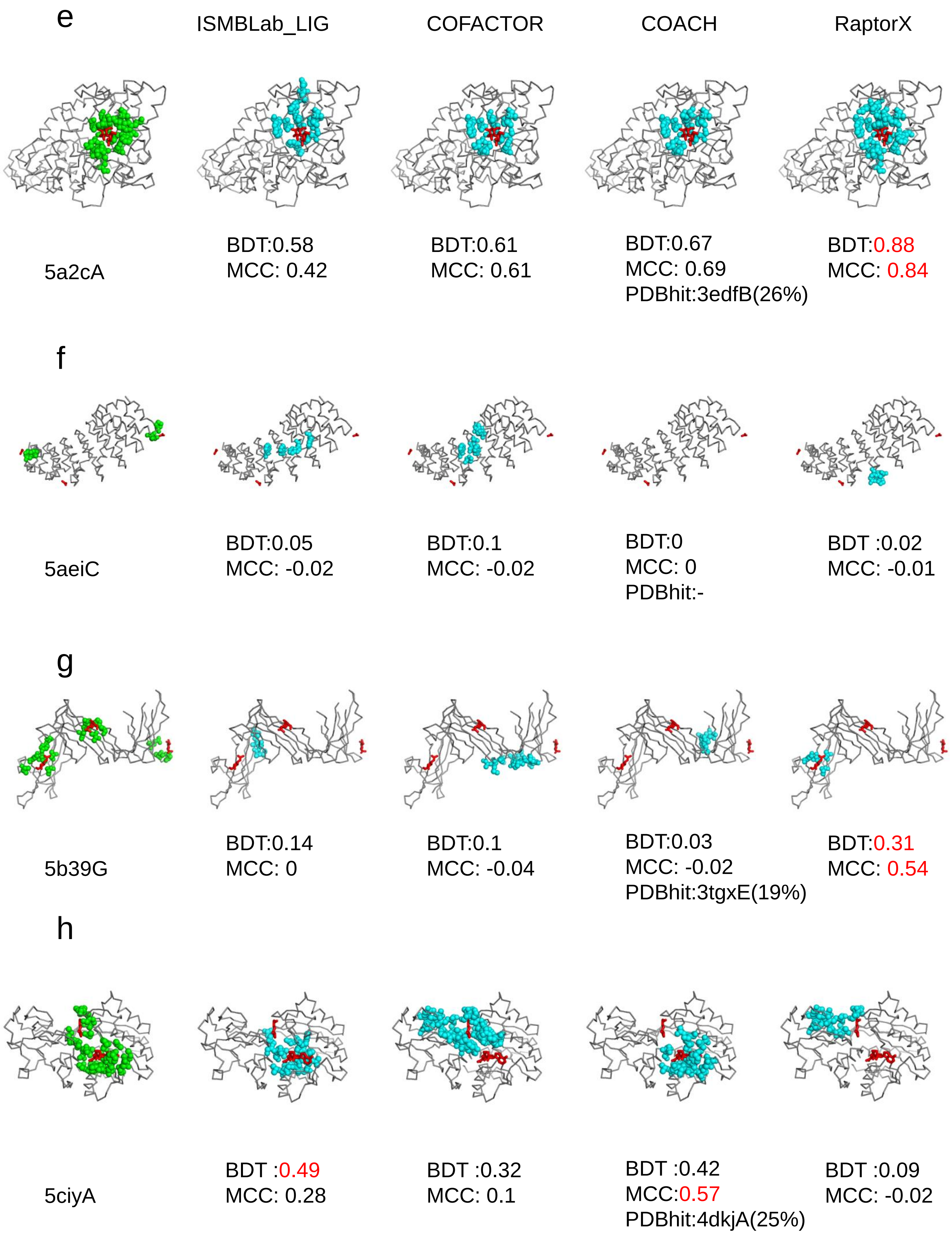
e
ISMBLab_LIG
COFACTOR
COACH
RaptorX
5a2cA
BDT:0.58
MCC: 0.42
BDT:0.61
MCC: 0.61
BDT:0.67
MCC: 0.69
PDBhit:3edfB(26%)
BDT:0.88
MCC: 0.84
f
5aeiC
BDT:0.05
MCC: -0.02
BDT:0.1
MCC: -0.02
BDT:0
MCC: 0
PDBhit:-
BDT :0.02
MCC: -0.01
g
5b39G
BDT:0.14
MCC: 0
BDT:0.1
MCC: -0.04
BDT:0.03
MCC: -0.02
PDBhit:3tgxE(19%)
BDT:0.31
MCC: 0.54
h
5ciyA
BDT :0.49
MCC: 0.28
BDT :0.32
MCC: 0.1
BDT :0.42
MCC:0.57
PDBhit:4dkjA(25%)
BDT :0.09
MCC: -0.02

S1 Fig

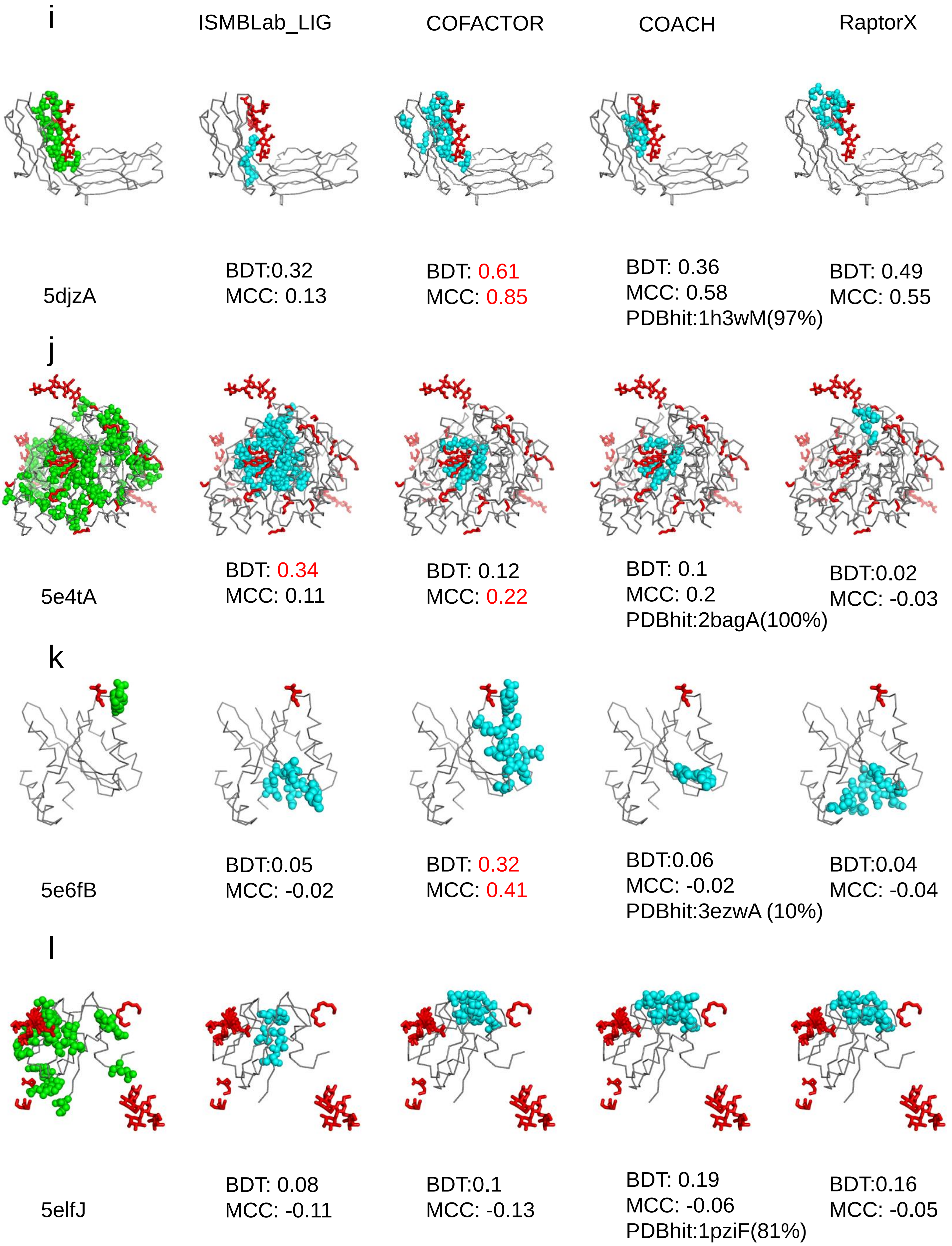
i
ISMBLab_LIG
COFACTOR
COACH
RaptorX
5djzA
BDT:0.32
MCC: 0.13
BDT: 0.61
MCC: 0.85
BDT: 0.36
MCC: 0.58
PDBhit:1h3wM(97%)
BDT: 0.49
MCC: 0.55
j
5e4tA
BDT: 0.34
MCC: 0.11
BDT: 0.12
MCC: 0.22
BDT: 0.1
MCC: 0.2
PDBhit:2bagA(100%)
BDT:0.02
MCC: -0.03
k
5e6fB
BDT:0.05
MCC: -0.02
BDT: 0.32
MCC: 0.41
BDT:0.06
MCC: -0.02
PDBhit:3ezwA (10%)
BDT:0.04
MCC: -0.04
l
5elfJ
BDT: 0.08
MCC: -0.11
BDT:0.1
MCC: -0.13
BDT: 0.19
MCC: -0.06
PDBhit:1pziF(81%)
BDT:0.16
MCC: -0.05

S1 Fig

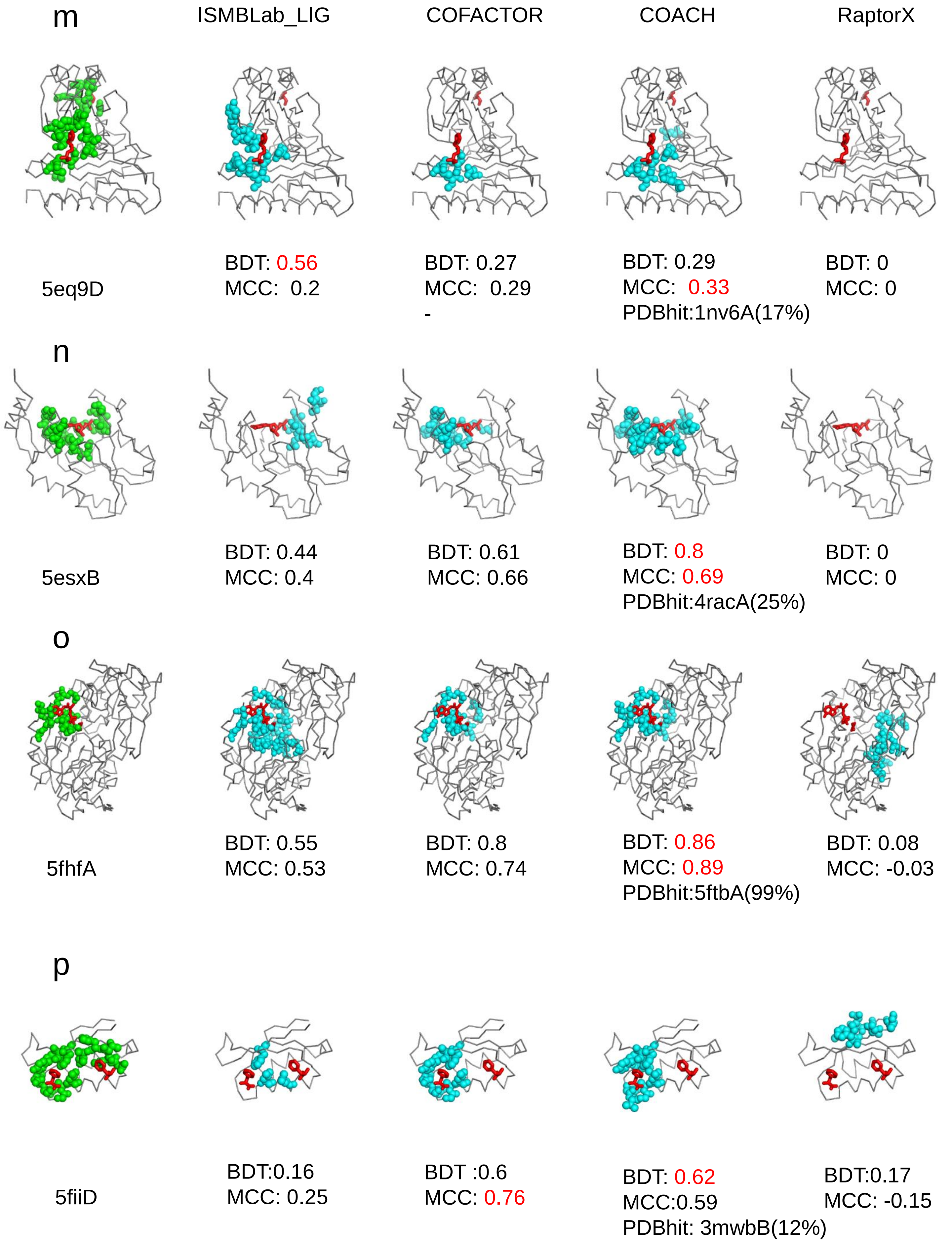
m
ISMBLab_LIG
COFACTOR
COACH
RaptorX
5eq9D
BDT: 0.56
MCC: 0.2
BDT: 0.27
MCC: 0.29
-
BDT: 0.29
MCC: 0.33
PDBhit:1nv6A(17%)
BDT: 0
MCC: 0
n
5esxB
BDT: 0.44
MCC: 0.4
BDT: 0.61
MCC: 0.66
BDT: 0.8
MCC: 0.69
PDBhit:4racA(25%)
BDT: 0
MCC: 0
o
5fhfA
BDT: 0.55
MCC: 0.53
BDT: 0.8
MCC: 0.74
BDT: 0.86
MCC: 0.89
PDBhit:5ftbA(99%)
BDT: 0.08
MCC: -0.03
p
5fiiD
BDT:0.16
MCC: 0.25
BDT :0.6
MCC: 0.76
BDT: 0.62
MCC:0.59
PDBhit: 3mwbB(12%)
BDT:0.17
MCC: -0.15

S1 Fig

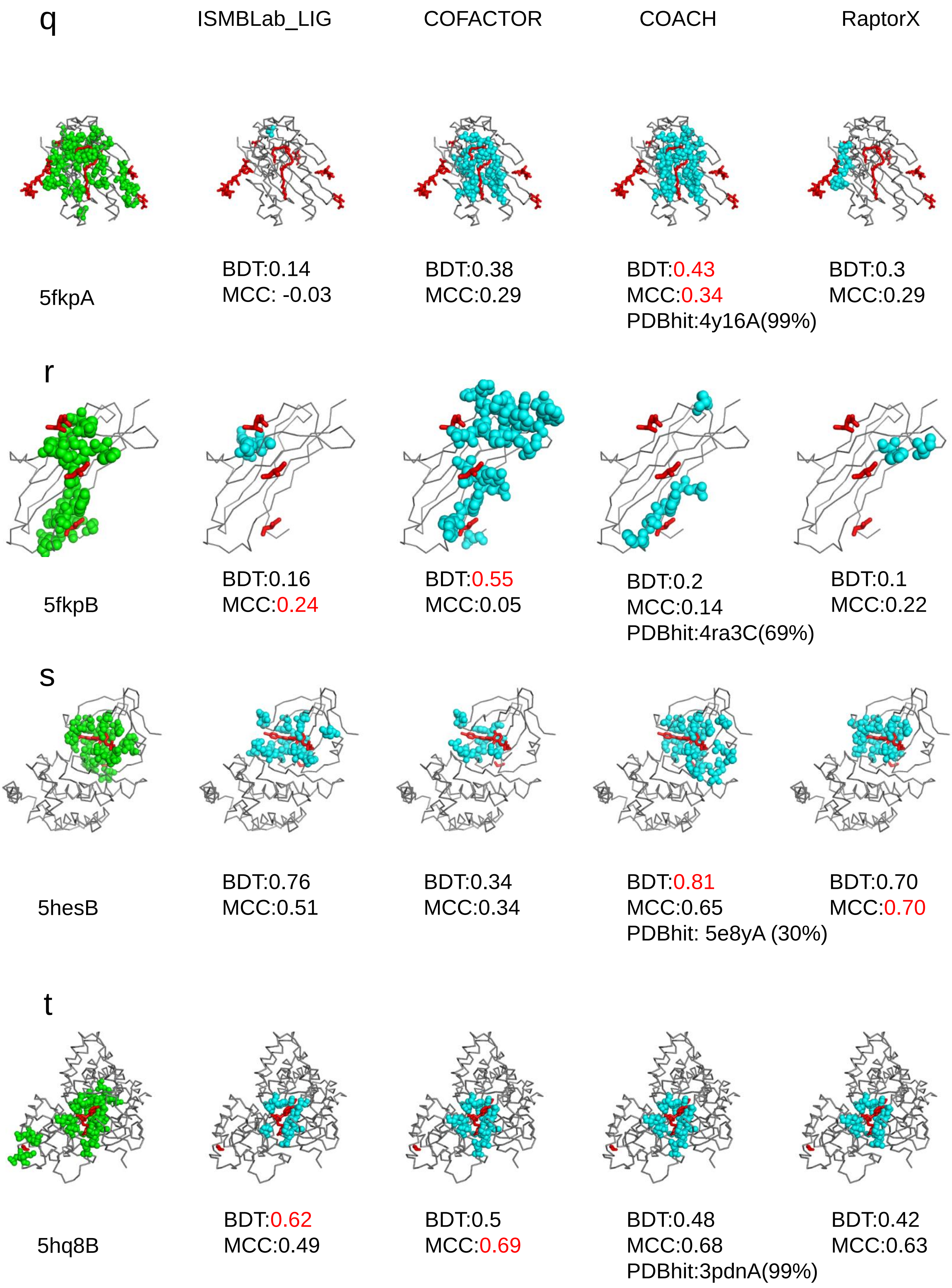
q
ISMBLab_LIG
COFACTOR
COACH
RaptorX
5fkpA
BDT:0.14
MCC: -0.03
BDT:0.38
MCC:0.29
BDT:0.43
MCC:0.34
PDBhit:4y16A(99%)
BDT:0.3
MCC:0.29
r
5fkpB
BDT:0.16
MCC:0.24
BDT:0.55
MCC:0.05
BDT:0.2
MCC:0.14
PDBhit:4ra3C(69%)
BDT:0.1
MCC:0.22
s
5hesB
BDT:0.76
MCC:0.51
BDT:0.34
MCC:0.34
BDT:0.81
MCC:0.65
PDBhit: 5e8yA (30%)
BDT:0.70
MCC:0.70
t
5hq8B
BDT:0.62
MCC:0.49
BDT:0.5
MCC:0.69
BDT:0.48
MCC:0.68
PDBhit:3pdnA(99%)
BDT:0.42
MCC:0.63

S1 Fig

u

ISMBLab_LIG COFACTOR COACH RaptorX

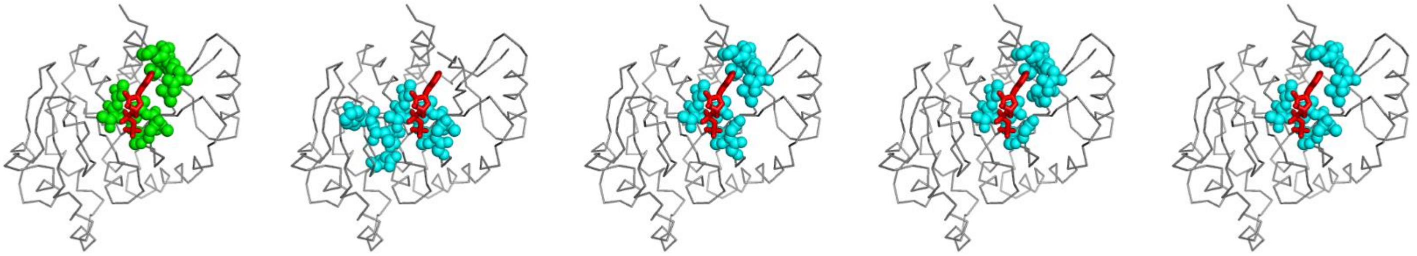

5ihpB

ISMBLab_LIG: MCC:0.5 BDT:0.5

COFACTOR: BDT:0.88 MCC:0.74

COACH: BDT:0.97 MCC:0.91 PDBhit:1ionA(31%)

RaptorX: BDT:0.91 MCC:0.95

v

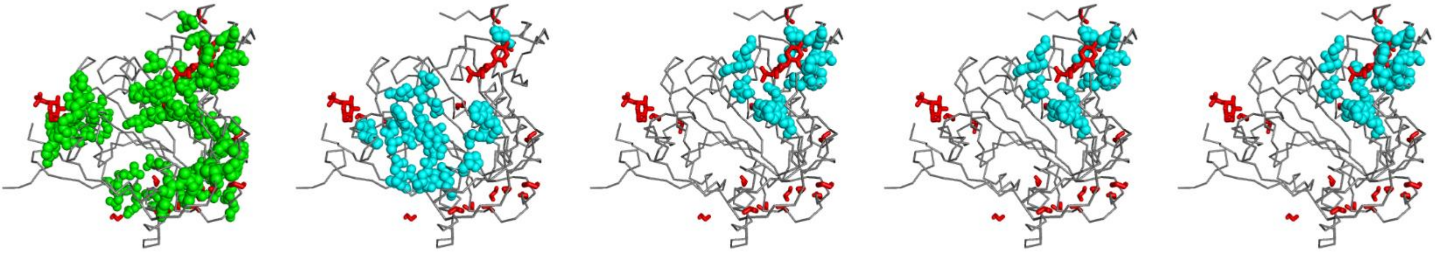

5in3B

ISMBLab_LIG: BDT:0.1 MCC: -0.02

COFACTOR: BDT:0.14 MCC:0.2

COACH: BDT:0.18 MCC:0.29 PDBhit:1guqC(56%)

RaptorX: BDT :0.25 MCC:0.39
