# Supplementary material for: Predicting Ligand Binding Sites on Protein Surfaces by 3-Dimensional Probability Density Distributions of Interacting Atoms
Source: PLoS One. 2016 Aug 11;11(8):e0160315. doi: 10.1371/journal.pone.0160315 (PMC4981321; doi:10.1371/journal.pone.0160315)
Supplement: S1 Table — The PDB ID, chain ID, and ligand name (columns 1~3) are downloaded from PDB; the prediction performances shown in columns 4~13 are defined in Eqs 5–10 of S1 Text; column 14 shows the number of LBS predicted for the corresponding protein structure (see Methods in main text); column 15 shows the number of the top one predicted LBS (see Methods in main text) for which the geometry center is within 4Å to the corresponding ligand. (DOCX) [file pone.0160315.s002.docx]

**S1 Table. ANN_BAGGING prediction accuracy benchmarks on the independent test set S48b. The PDB ID, chain ID, and ligand name (columns 1~3) are downloaded from PDB; the prediction performances shown in columns 4~13 are defined in Equations (5)~(10) of Supplementary Methods; column 14 shows the number of LBS predicted for the corresponding protein structure (see Methods in main text); column 15 shows the number of the top one predicted LBS (see Methods in main text) for which the geometry center is within 4Å to the corresponding ligand.**

| PDB | Cha | Lig | Acc | Pre | Rec | Spe | Mcc | Fsc | TP | TN | FP | FN | NP | Suc |
| --- | --- | --- | --- | --- | --- | --- | --- | --- | --- | --- | --- | --- | --- | --- |
| 1a6w | L,H | NIP | 0.97 | 0.62 | 0.89 | 0.97 | 0.73 | 0.73 | 8 | 189 | 5 | 1 | 2 | 1 |
| 1acj | A | THA | 0.96 | 0.41 | 1.00 | 0.96 | 0.63 | 0.58 | 11 | 403 | 16 | 0 | 1 | 1 |
| 1apu | E | MAN | 0.91 | 0.00 | 0.00 | 0.92 | 0.04 | 0.00 | 0 | 270 | 24 | 4 | 1 | 0 |
| 1bid | A | UMP\|CBX | 0.94 | 0.53 | 0.53 | 0.97 | 0.50 | 0.53 | 8 | 222 | 7 | 7 | 2 | 1 |
| 1blh | A | FOS | 0.95 | 0.46 | 0.50 | 0.97 | 0.45 | 0.48 | 5 | 201 | 6 | 5 | 2 | 1 |
| 1byb | A | GLC | 0.94 | 0.49 | 0.59 | 0.96 | 0.50 | 0.53 | 16 | 404 | 17 | 11 | 4 | 1 |
| 1cdo | A | NAD | 0.96 | 0.69 | 0.93 | 0.96 | 0.78 | 0.79 | 25 | 289 | 11 | 2 | 2 | 1 |
| 1dwd | H,I,L | MID | 0.96 | 0.71 | 0.75 | 0.98 | 0.71 | 0.73 | 15 | 235 | 6 | 5 | 2 | 1 |
| 1fbp | A | F6P\|AMP | 0.91 | 0.52 | 0.54 | 0.95 | 0.48 | 0.53 | 14 | 250 | 13 | 12 | 2 | 1 |
| 1gca | A | GAL | 0.98 | 1.00 | 0.62 | 1.00 | 0.78 | 0.76 | 8 | 258 | 0 | 5 | 1 | 1 |
| 1hew | A | NAG | 0.87 | 0.00 | 0.00 | 0.95 | 0.07 | 0.00 | 0 | 102 | 5 | 10 | 1 | 0 |
| 1hfc | A | HAP | 0.92 | 0.64 | 0.47 | 0.97 | 0.50 | 0.54 | 7 | 126 | 4 | 8 | 2 | 1 |
| 1hyt | A | BZS\|DMS | 0.97 | 0.81 | 0.72 | 0.99 | 0.75 | 0.77 | 13 | 269 | 3 | 5 | 1 | 1 |
| 1ida | A,I,B | QND\|PY2\|PPL\|HPB | 0.94 | 0.88 | 0.63 | 0.99 | 0.71 | 0.73 | 15 | 152 | 2 | 9 | 1 | 1 |
| 1igj | B | DGX | 1.00 | 0.83 | 1.00 | 1.00 | 0.91 | 0.91 | 10 | 388 | 2 | 0 | 1 | 1 |
| 1imb | A | LIP | 0.95 | 0.50 | 0.71 | 0.96 | 0.57 | 0.59 | 10 | 229 | 10 | 4 | 2 | 1 |
| 1inc | A | ICL | 0.97 | 0.89 | 0.62 | 1.00 | 0.73 | 0.73 | 8 | 208 | 1 | 5 | 1 | 1 |
| 1ivd | A | NAG\|ST1\|FUC\|MAN | 0.92 | 0.19 | 0.16 | 0.96 | 0.13 | 0.17 | 3 | 323 | 13 | 16 | 4 | 0 |
| 1mrg | A | ADN | 0.93 | 0.33 | 0.60 | 0.94 | 0.41 | 0.43 | 6 | 203 | 12 | 4 | 1 | 1 |
| 1mtw | A | DX9 | 0.96 | 0.67 | 0.86 | 0.97 | 0.74 | 0.75 | 12 | 185 | 6 | 2 | 1 | 1 |
| 1okm | A | SAB | 0.95 | 0.52 | 1.00 | 0.95 | 0.70 | 0.69 | 12 | 207 | 11 | 0 | 2 | 1 |
| 1pdz | A | PGA | 0.98 | 0.46 | 1.00 | 0.98 | 0.67 | 0.63 | 6 | 357 | 7 | 0 | 1 | 1 |
| 1phd | A | HEM\|PIM | 0.91 | 0.00 | 0.00 | 0.99 | 0.03 | 0.00 | 0 | 334 | 3 | 31 | 1 | 0 |
| 1pso | I,E | STA\|IVA | 0.96 | 0.71 | 0.63 | 0.98 | 0.65 | 0.67 | 12 | 276 | 5 | 7 | 1 | 1 |
| 1qpe | A | PP2\|PTR | 0.94 | 0.68 | 0.62 | 0.97 | 0.62 | 0.65 | 13 | 224 | 6 | 8 | 1 | 1 |
| 1rbp | A | RTL | 0.95 | 0.70 | 0.89 | 0.95 | 0.76 | 0.78 | 16 | 145 | 7 | 2 | 1 | 1 |
| 1rne | A | NAG\|C60 | 0.92 | 0.65 | 0.52 | 0.97 | 0.54 | 0.58 | 15 | 253 | 8 | 14 | 3 | 1 |
| 1rob | A | C2P | 0.98 | 1.00 | 0.75 | 1.00 | 0.86 | 0.86 | 6 | 107 | 0 | 2 | 1 | 1 |
| 1snc | A | PTP | 0.91 | 0.40 | 0.40 | 0.95 | 0.35 | 0.40 | 4 | 110 | 6 | 6 | 1 | 1 |
| 1srf | A,B | MTB | 0.92 | 0.94 | 0.52 | 0.99 | 0.66 | 0.67 | 17 | 179 | 1 | 16 | 4 | 1 |
| 1stp | A | BTN | 0.96 | 0.92 | 0.75 | 0.99 | 0.81 | 0.83 | 12 | 99 | 1 | 4 | 1 | 1 |
| 1ulb | A | GUN | 0.93 | 0.26 | 0.56 | 0.95 | 0.35 | 0.36 | 5 | 244 | 14 | 4 | 2 | 1 |
| 2ctc | A | LOF | 0.94 | 0.45 | 1.00 | 0.94 | 0.65 | 0.62 | 13 | 242 | 16 | 0 | 2 | 1 |
| 2h4n | A | AZM | 0.96 | 0.53 | 1.00 | 0.96 | 0.71 | 0.69 | 10 | 212 | 9 | 0 | 2 | 1 |
| 2ifb | A | PLM | 0.88 | 0.75 | 0.18 | 0.99 | 0.33 | 0.29 | 3 | 109 | 1 | 14 | 1 | 1 |
| 2pk4 | A | ACA | 0.97 | 1.00 | 0.71 | 1.00 | 0.83 | 0.83 | 5 | 69 | 0 | 2 | 1 | 1 |
| 2sim | A | DAN | 0.97 | 0.52 | 1.00 | 0.97 | 0.71 | 0.69 | 11 | 317 | 10 | 0 | 2 | 1 |
| 2tmn | E | PHO\|NH2 | 0.97 | 0.50 | 1.00 | 0.97 | 0.70 | 0.67 | 9 | 270 | 9 | 0 | 1 | 1 |
| 2ypi | A | PGA | 0.98 | 0.73 | 1.00 | 0.98 | 0.85 | 0.85 | 11 | 210 | 4 | 0 | 1 | 1 |
| 3gch | A | CIN | 0.94 | 0.48 | 0.92 | 0.94 | 0.64 | 0.63 | 11 | 199 | 12 | 1 | 2 | 1 |
| 3mth | A,B,C,D | MPB | 0.00 | 0.00 | 0.00 | 0.00 | 0.00 | 0.00 | 0 | 86 | 0 | 6 | 0 | 0 |
| 3ptb | A | BEN | 0.97 | 0.61 | 1.00 | 0.97 | 0.77 | 0.76 | 11 | 191 | 7 | 0 | 1 | 1 |
| 4dfr | A | MTX | 0.86 | 0.42 | 0.63 | 0.89 | 0.43 | 0.50 | 10 | 110 | 14 | 6 | 2 | 1 |
| 4phv | A,B | VAC | 0.97 | 1.00 | 0.83 | 1.00 | 0.90 | 0.91 | 25 | 150 | 0 | 5 | 2 | 1 |
| 5cna | A | MMA | 0.99 | 0.82 | 1.00 | 0.99 | 0.90 | 0.90 | 9 | 201 | 2 | 0 | 1 | 1 |
| 5p2p | A | DHG | 0.00 | 0.00 | 0.00 | 0.00 | 0.00 | 0.00 | 0 | 92 | 0 | 20 | 0 | 0 |
| 6rsa | A | UVC | 0.00 | 0.00 | 0.00 | 0.00 | 0.00 | 0.00 | 0 | 14 | 0 | 0 | 0 | 0 |
| 7cpa | A | FVF | 0.93 | 0.42 | 0.93 | 0.93 | 0.60 | 0.58 | 13 | 243 | 18 | 1 | 2 | 1 |
|  |  | Total | 0.95 | 0.58 | 0.63 | 0.97 | 0.58 | 0.60 | 453 | 10156 | | 334 | 264 |  |
