# Supplementary material for: Predicting Ligand Binding Sites on Protein Surfaces by 3-Dimensional Probability Density Distributions of Interacting Atoms
Source: PLoS One. 2016 Aug 11;11(8):e0160315. doi: 10.1371/journal.pone.0160315 (PMC4981321; doi:10.1371/journal.pone.0160315)
Supplement: S2 Table — The PDB ID, chain ID, and ligand name (columns 1~3) are downloaded from PDB; the prediction performances shown in columns 4~13 are defined in Eqs 5–10 of S1 Text; column 14 shows the number of LBS predicted for the corresponding protein structure (see Methods in main text); column 15 shows the number of the top one predicted LBS (see Methods in main text) for which the geometry center is within 4Å to the corresponding ligand. (DOCX) [file pone.0160315.s003.docx]

**S2 Table. ANN_BAGGING prediction accuracy benchmarks on the independent test set S48ub. The PDB ID, chain ID, and ligand name (columns 1~3) are downloaded from PDB; the prediction performances shown in columns 4~13 are defined in Equations (5)~(10) of Supplementary Methods; column 14 shows the number of LBS predicted for the corresponding protein structure (see Methods in main text); column 15 shows the number of the top one predicted LBS (see Methods in main text) for which the geometry center is within 4Å to the corresponding ligand.**

| PDB | Cha | Lig | | Acc | | Pre | | Rec | | Spe | | Mcc | | Fsc | | TP | | TN | | FP | | | FN | | NP | Suc | |  |
| --- | --- | --- | --- | --- | --- | --- | --- | --- | --- | --- | --- | --- | --- | --- | --- | --- | --- | --- | --- | --- | --- | --- | --- | --- | --- | --- | --- | --- |
| 1a4j | L | | DGX | | 0.96 | | 0.24 | | 0.44 | | 0.97 | | 0.30 | | 0.31 | | 4 | | 385 | | 13 | | | 5 | 4 | | 1 | |
| 1a6u | H,L | | NIP | | 0.97 | | 0.64 | | 0.78 | | 0.98 | | 0.69 | | 0.70 | | 7 | | 194 | | 4 | | | 2 | 2 | | 1 | |
| 1ahc | A | | ADN | | 0.95 | | 0.46 | | 1.00 | | 0.94 | | 0.66 | | 0.63 | | 10 | | 199 | | 12 | | | 0 | 2 | | 1 | |
| 1bbs | A | | NAG\|C60 | | 0.94 | | 0.70 | | 0.59 | | 0.97 | | 0.61 | | 0.64 | | 16 | | 259 | | 7 | | | 11 | 2 | | 1 | |
| 1brq | A | | RTL | | 0.92 | | 0.55 | | 0.80 | | 0.94 | | 0.62 | | 0.65 | | 12 | | 146 | | 10 | | | 3 | 1 | | 1 | |
| 1bya | A | | GLC | | 0.96 | | 0.59 | | 0.74 | | 0.97 | | 0.64 | | 0.65 | | 17 | | 403 | | 12 | | | 6 | 4 | | 1 | |
| 1cge | A | | HAP | | 0.93 | | 0.86 | | 0.40 | | 0.99 | | 0.56 | | 0.55 | | 6 | | 131 | | 1 | | | 9 | 1 | | 1 | |
| 1chg | A | | CIN | | 0.96 | | 0.50 | | 0.56 | | 0.98 | | 0.50 | | 0.53 | | 5 | | 194 | | 5 | | | 4 | 2 | | 1 | |
| 1djb | A | | FOS | | 0.96 | | 0.71 | | 0.46 | | 0.99 | | 0.55 | | 0.56 | | 5 | | 208 | | 2 | | | 6 | 1 | | 1 | |
| 1esa | A | | ICL | | 0.98 | | 0.85 | | 0.79 | | 0.99 | | 0.80 | | 0.82 | | 11 | | 207 | | 2 | | | 3 | 1 | | 1 | |
| 1gcg | A | | GAL | | 0.97 | | 0.69 | | 0.75 | | 0.98 | | 0.71 | | 0.72 | | 9 | | 251 | | 4 | | | 3 | 1 | | 1 | |
| 1hel | A | | NAG | | 0.00 | | 0.00 | | 0.00 | | 0.00 | | 0.00 | | 0.00 | | 0 | | 105 | | 0 | | | 10 | 0 | | 0 | |
| 1hsi | A,B | | QND\|PY2\|PPL\|HPB | | 0.92 | | 0.58 | | 0.44 | | 0.97 | | 0.46 | | 0.50 | | 7 | | 159 | | 5 | | | 9 | 2 | | 1 | |
| 1hxf | H,I,L | | MID | | 0.94 | | 0.56 | | 0.79 | | 0.95 | | 0.63 | | 0.65 | | 15 | | 227 | | 12 | | | 4 | 2 | | 1 | |
| 1ifb | A | | PLM | | 0.00 | | 0.00 | | 0.00 | | 0.00 | | 0.00 | | 0.00 | | 0 | | 112 | | 0 | | | 14 | 0 | | 0 | |
| 1ime | A | | LIP | | 0.95 | | 0.50 | | 0.92 | | 0.95 | | 0.66 | | 0.65 | | 12 | | 229 | | 12 | | | 1 | 2 | | 1 | |
| 1krn | A | | ACA | | 0.97 | | 1.00 | | 0.71 | | 1.00 | | 0.83 | | 0.83 | | 5 | | 69 | | 0 | | | 2 | 1 | | 1 | |
| 1l3f | E | | PHO\|NH2 | | 0.99 | | 0.70 | | 1.00 | | 0.99 | | 0.83 | | 0.82 | | 7 | | 276 | | 3 | | | 0 | 1 | | 1 | |
| 1nna | A | | NAG\|ST1\|FUC\|MAN | | 0.93 | | 0.13 | | 0.05 | | 0.98 | | 0.25 | | 0.07 | | 1 | | 335 | | 7 | | | 19 | 2 | | 1 | |
| 1npc | A | | BZS\|DMS | | 0.96 | | 0.57 | | 0.29 | | 0.99 | | 0.38 | | 0.38 | | 4 | | 281 | | 3 | | | 10 | 1 | | 1 | |
| 1pdy | A | | PGA | | 0.97 | | 0.15 | | 0.50 | | 0.97 | | 0.26 | | 0.24 | | 2 | | 353 | | 11 | | | 2 | 1 | | 1 | |
| 1phc | A | | HEM\|PIM | | 0.89 | | 0.00 | | 0.00 | | 0.98 | | 0.05 | | 0.00 | | 0 | | 330 | | 8 | | | 31 | 2 | | 0 | |
| 1psn | A | | STA\|IVA | | 0.95 | | 0.55 | | 0.69 | | 0.97 | | 0.59 | | 0.61 | | 11 | | 274 | | 9 | | | 5 | 2 | | 1 | |
| 1pts | A,B | | MTB | | 0.96 | | 0.95 | | 0.69 | | 1.00 | | 0.79 | | 0.80 | | 18 | | 188 | | 1 | | | 8 | 2 | | 1 | |
| 1qif | A | | THA | | 0.97 | | 0.30 | | 1.00 | | 0.97 | | 0.54 | | 0.47 | | 7 | | 436 | | 16 | | | 0 | 2 | | 1 | |
| 1stn | A | | PTP | | 0.00 | | 0.00 | | 0.00 | | 0.00 | | 0.00 | | 0.00 | | 0 | | 116 | | 0 | | | 9 | 0 | | 0 | |
| 1swb | A | | BTN | | 0.96 | | 0.92 | | 0.73 | | 0.99 | | 0.80 | | 0.82 | | 11 | | 101 | | 1 | | | 4 | 1 | | 1 | |
| 1ula | A | | GUN | | 0.93 | | 0.37 | | 0.91 | | 0.93 | | 0.56 | | 0.53 | | 10 | | 233 | | 17 | | | 1 | 1 | | 1 | |
| 1ypi | A | | PGA | | 0.97 | | 0.58 | | 0.88 | | 0.98 | | 0.70 | | 0.70 | | 7 | | 212 | | 5 | | | 1 | 1 | | 1 | |
| 2cba | A | | AZM | | 0.94 | | 0.41 | | 1.00 | | 0.94 | | 0.62 | | 0.58 | | 9 | | 206 | | 13 | | | 0 | 2 | | 1 | |
| 2ctb | A | | LOF | | 0.93 | | 0.31 | | 1.00 | | 0.93 | | 0.54 | | 0.47 | | 8 | | 247 | | 18 | | | 0 | 2 | | 1 | |
| 2ctv | A | | MMA | | 0.99 | | 0.80 | | 1.00 | | 0.99 | | 0.89 | | 0.89 | | 8 | | 203 | | 2 | | | 0 | 1 | | 1 | |
| 2fbp | A | | F6P\|AMP | | 0.93 | | 0.63 | | 0.60 | | 0.97 | | 0.58 | | 0.61 | | 15 | | 250 | | 9 | | | 10 | 3 | | 1 | |
| 2sil | A | | DAN | | 0.98 | | 0.63 | | 0.91 | | 0.98 | | 0.74 | | 0.74 | | 10 | | 321 | | 6 | | | 1 | 2 | | 1 | |
| 2tga | A | | DX9 | | 0.00 | | 0.00 | | 0.00 | | 0.00 | | 0.00 | | 0.00 | | 0 | | 191 | | 0 | | | 13 | 0 | | 0 | |
| 3app | A | | MAN | | 0.93 | | 0.00 | | 0.00 | | 0.94 | | 0.03 | | 0.00 | | 0 | | 278 | | 17 | | | 4 | 1 | | 0 | |
| 3lck | A | | PP2\|PTR | | 0.93 | | 0.46 | | 0.35 | | 0.97 | | 0.37 | | 0.40 | | 6 | | 230 | | 7 | | | 11 | 1 | | 1 | |
| 3p2p | A | | DHG | | 0.00 | | 0.00 | | 0.00 | | 0.00 | | 0.00 | | 0.00 | | 0 | | 93 | | 0 | | | 18 | 0 | | 0 | |
| 3phv | A,B | | VAC | | 0.92 | | 0.60 | | 0.38 | | 0.98 | | 0.44 | | 0.46 | | 6 | | 165 | | 4 | | | 10 | 1 | | 1 | |
| 3ptn | A | | BEN | | 0.96 | | 0.56 | | 1.00 | | 0.96 | | 0.73 | | 0.71 | | 10 | | 188 | | 8 | | | 0 | 1 | | 1 | |
| 3tms | A | | UMP\|CBX | | 0.94 | | 0.45 | | 0.64 | | 0.95 | | 0.51 | | 0.53 | | 9 | | 222 | | 11 | | | 5 | 2 | | 1 | |
| 4ca2 | A | | SAB | | 0.95 | | 0.52 | | 0.85 | | 0.95 | | 0.64 | | 0.65 | | 11 | | 208 | | 10 | | | 2 | 2 | | 1 | |
| 5cpa | A | | FVF | | 0.91 | | 0.30 | | 0.50 | | 0.94 | | 0.35 | | 0.38 | | 7 | | 238 | | 16 | | | 7 | 2 | | 1 | |
| 5dfr | A | | MTX | | 0.91 | | 0.47 | | 0.64 | | 0.94 | | 0.50 | | 0.54 | | 7 | | 116 | | 8 | | | 4 | 2 | | 1 | |
| 6ins | E | | MPB | | 0.00 | | 0.00 | | 0.00 | | 0.00 | | 0.00 | | 0.00 | | 0 | | 83 | | 0 | | | 7 | 0 | | 0 | |
| 7rat | A | | UVC | | 0.97 | | 0.75 | | 0.75 | | 0.98 | | 0.73 | | 0.75 | | 6 | | 104 | | 2 | | | 2 | 2 | | 1 | |
| 8adh | A | | NAD | | 0.94 | | 0.49 | | 0.80 | | 0.94 | | 0.59 | | 0.60 | | 16 | | 287 | | 17 | | | 4 | 1 | | 1 | |
| 8rat | A | | C2P | | 0.91 | | 0.14 | | 0.04 | | 0.94 | | 0.20 | | 0.21 | | 1 | | 106 | | 2 | | | 8 | 1 | | 1 | |
|  |  | | Total | | 0.95 | | 0.51 | | 0.55 | | 0.97 | | 0.50 | | 0.53 | | 347 | | 10349 | | | 330 288 | | | | |  | |
