# Supplementary material for: Predicting Ligand Binding Sites on Protein Surfaces by 3-Dimensional Probability Density Distributions of Interacting Atoms
Source: PLoS One. 2016 Aug 11;11(8):e0160315. doi: 10.1371/journal.pone.0160315 (PMC4981321; doi:10.1371/journal.pone.0160315)
Supplement: S3 Table — The PDB ID, chain ID, and ligand name (columns 1~3) are downloaded from PDB; the prediction performances shown in columns 4~13 are defined in Eqs 5–10 of S1 Text; column 14 shows the number of LBS predicted for the corresponding protein structure (see Methods in main text); column 15 shows the number of the top one predicted LBS (see Methods in main text) for which the geometry center is within 4Å to the corresponding ligand. (DOCX) [file pone.0160315.s004.docx]

**S3 Table. ANN_BAGGING prediction accuracy benchmarks on the independent test set S210. The PDB ID, chain ID, and ligand name (columns 1~3) are downloaded from PDB; the prediction performances shown in columns 4~13 are defined in Equations (5)~(10) of Supplementary Methods; column 14 shows the number of LBS predicted for the corresponding protein structure (see Methods in main text); column 15 shows the number of the top one predicted LBS (see Methods in main text) for which the geometry center is within 4Å to the corresponding ligand.**

| PDB | Cha | Lig | | | Acc | | Pre | | Rec | | Spe | | Mcc | | Fsc | | TP | | TN | | FP | | FN | | NP | | Suc | |
| --- | --- | --- | --- | --- | --- | --- | --- | --- | --- | --- | --- | --- | --- | --- | --- | --- | --- | --- | --- | --- | --- | --- | --- | --- | --- | --- | --- | --- |
| 1a0q | H,L | | HEP | 0.98 | | 0.56 | | 0.71 | | 0.99 | | 0.62 | | 0.63 | | 5 | | 368 | | 4 | | 2 | | 4 | | 1 | |  |
| 1a28 | A | | STR | 0.94 | | 0.00 | | 0.00 | | 0.99 | | 0.03 | | 0.00 | | 0 | | 222 | | 3 | | 12 | | 1 | | 0 | |  |
| 1a42 | A | | BZO\|HG | 0.93 | | 0.48 | | 0.63 | | 0.95 | | 0.51 | | 0.54 | | 10 | | 200 | | 11 | | 6 | | 2 | | 1 | |  |
| 1a4g | A | | NAG\|ZMR | 0.94 | | 0.42 | | 0.25 | | 0.98 | | 0.29 | | 0.31 | | 5 | | 324 | | 7 | | 15 | | 6 | | 1 | |  |
| 1a6w | L,H | | NIP | 0.97 | | 0.64 | | 0.78 | | 0.98 | | 0.69 | | 0.70 | | 7 | | 190 | | 4 | | 2 | | 2 | | 1 | |  |
| 1a9u | A | | SB2 | 0.97 | | 0.67 | | 0.20 | | 1.00 | | 0.36 | | 0.31 | | 2 | | 313 | | 1 | | 8 | | 1 | | 1 | |  |
| 1aaq | A,B | | PSI | 0.92 | | 0.57 | | 0.47 | | 0.97 | | 0.48 | | 0.52 | | 8 | | 167 | | 6 | | 9 | | 1 | | 1 | |  |
| 1abe | A | | ARB\|ARA | 0.00 | | 0.00 | | 0.00 | | 0.00 | | 0.00 | | 0.00 | | 0 | | 246 | | 0 | | 12 | | 0 | | 0 | |  |
| 1ac0 | A | | GLC | 0.00 | | 0.00 | | 0.00 | | 0.00 | | 0.00 | | 0.00 | | 0 | | 85 | | 0 | | 16 | | 0 | | 0 | |  |
| 1acj | A | | THA | 0.97 | | 0.39 | | 0.90 | | 0.97 | | 0.58 | | 0.55 | | 9 | | 406 | | 14 | | 1 | | 1 | | 1 | |  |
| 1aco | A | | TRA\|FS4 | 0.96 | | 0.00 | | 0.00 | | 0.99 | | 0.02 | | 0.00 | | 0 | | 648 | | 9 | | 20 | | 3 | | 0 | |  |
| 1adb | A | | EOH\|CND | 0.96 | | 0.69 | | 0.88 | | 0.97 | | 0.76 | | 0.77 | | 22 | | 291 | | 10 | | 3 | | 2 | | 1 | |  |
| 1add | A | | 1DA | 0.96 | | 0.54 | | 1.00 | | 0.96 | | 0.72 | | 0.70 | | 13 | | 271 | | 11 | | 0 | | 2 | | 1 | |  |
| 1adf | A | | TAD | 0.93 | | 0.42 | | 0.50 | | 0.95 | | 0.42 | | 0.46 | | 10 | | 289 | | 14 | | 10 | | 2 | | 1 | |  |
| 1aec | A | | E64 | 0.91 | | 0.00 | | 0.00 | | 0.98 | | 0.04 | | 0.00 | | 0 | | 178 | | 4 | | 13 | | 2 | | 0 | |  |
| 1aha | A | | ADE | 0.96 | | 0.55 | | 0.60 | | 0.98 | | 0.55 | | 0.57 | | 6 | | 208 | | 5 | | 4 | | 1 | | 1 | |  |
| 1ai5 | A,B | | MNP | 0.98 | | 0.29 | | 0.50 | | 0.99 | | 0.37 | | 0.36 | | 4 | | 690 | | 10 | | 4 | | 6 | | 1 | |  |
| 1aj7 | H,L | | NPE | 0.98 | | 0.64 | | 0.58 | | 0.99 | | 0.60 | | 0.61 | | 7 | | 389 | | 4 | | 5 | | 3 | | 1 | |  |
| 1ake | A | | AP5 | 0.90 | | 0.65 | | 0.59 | | 0.95 | | 0.56 | | 0.62 | | 17 | | 166 | | 9 | | 12 | | 1 | | 1 | |  |
| 1anf | A | | GLC | 0.97 | | 0.56 | | 0.82 | | 0.98 | | 0.67 | | 0.67 | | 9 | | 326 | | 7 | | 2 | | 1 | | 1 | |  |
| 1aoe | A | | NDP\|GW3 | 0.92 | | 0.89 | | 0.59 | | 0.99 | | 0.69 | | 0.71 | | 16 | | 141 | | 2 | | 11 | | 1 | | 1 | |  |
| 1apt | E | | IVA\|MAN\|LTA | 0.97 | | 0.73 | | 0.65 | | 0.99 | | 0.67 | | 0.69 | | 11 | | 274 | | 4 | | 6 | | 2 | | 1 | |  |
| 1ase | A | | MAE\|NOP | 0.98 | | 0.67 | | 1.00 | | 0.98 | | 0.81 | | 0.80 | | 16 | | 343 | | 8 | | 0 | | 1 | | 1 | |  |
| 1azm | A | | AZM | 0.97 | | 0.53 | | 1.00 | | 0.97 | | 0.72 | | 0.70 | | 8 | | 225 | | 7 | | 0 | | 1 | | 1 | |  |
| 1b59 | A | | OVA | 0.94 | | 0.33 | | 0.64 | | 0.95 | | 0.43 | | 0.44 | | 7 | | 290 | | 14 | | 4 | | 2 | | 1 | |  |
| 1b6n | A,B | | PI3\|ABA | 0.83 | | 0.53 | | 0.25 | | 0.95 | | 0.28 | | 0.34 | | 8 | | 143 | | 7 | | 24 | | 1 | | 1 | |  |
| 1b9v | A | | RA2\|NAG | 0.94 | | 0.44 | | 0.19 | | 0.99 | | 0.26 | | 0.27 | | 4 | | 333 | | 5 | | 17 | | 2 | | 1 | |  |
| 1baf | H,L | | NPP | 0.97 | | 0.29 | | 0.71 | | 0.97 | | 0.44 | | 0.42 | | 5 | | 378 | | 12 | | 2 | | 2 | | 1 | |  |
| 1bap | A | | ARB\|ARA | 0.00 | | 0.00 | | 0.00 | | 0.00 | | 0.00 | | 0.00 | | 0 | | 248 | | 0 | | 12 | | 0 | | 0 | |  |
| 1bcd | A | | FMS | 0.96 | | 0.50 | | 1.00 | | 0.96 | | 0.69 | | 0.67 | | 9 | | 214 | | 9 | | 0 | | 2 | | 1 | |  |
| 1bgo | A | | I10 | 0.90 | | 0.00 | | 0.00 | | 0.99 | | 0.03 | | 0.00 | | 0 | | 174 | | 2 | | 17 | | 1 | | 0 | |  |
| 1bhf | A | | ACE\|1PA | 0.91 | | 0.00 | | 0.00 | | 0.98 | | 0.04 | | 0.00 | | 0 | | 86 | | 2 | | 7 | | 1 | | 0 | |  |
| 1bl7 | A | | SB4 | 0.97 | | 0.50 | | 0.44 | | 0.99 | | 0.46 | | 0.47 | | 4 | | 313 | | 4 | | 5 | | 1 | | 1 | |  |
| 1blh | A | | FOS | 0.96 | | 0.56 | | 0.56 | | 0.98 | | 0.54 | | 0.56 | | 5 | | 204 | | 4 | | 4 | | 2 | | 1 | |  |
| 1bma | A | | MBH\|ISO\|TFA | 0.96 | | 0.70 | | 0.54 | | 0.99 | | 0.59 | | 0.61 | | 7 | | 204 | | 3 | | 6 | | 1 | | 1 | |  |
| 1bmc | A,B | | MNO | 0.00 | | 0.00 | | 0.00 | | 0.00 | | 0.00 | | 0.00 | | 0 | | 223 | | 0 | | 10 | | 0 | | 0 | |  |
| 1bra | A | | BEN | 0.94 | | 0.47 | | 0.64 | | 0.96 | | 0.52 | | 0.54 | | 7 | | 189 | | 8 | | 4 | | 1 | | 1 | |  |
| 1byb | A | | GLC | 0.95 | | 0.54 | | 0.61 | | 0.97 | | 0.55 | | 0.57 | | 14 | | 413 | | 12 | | 9 | | 4 | | 1 | |  |
| 1byg | A | | STU | 0.97 | | 0.82 | | 0.69 | | 0.99 | | 0.74 | | 0.75 | | 9 | | 211 | | 2 | | 4 | | 1 | | 1 | |  |
| 1c2t | A | | GAR\|NHS | 0.91 | | 0.71 | | 0.44 | | 0.98 | | 0.51 | | 0.54 | | 10 | | 161 | | 4 | | 13 | | 1 | | 1 | |  |
| 1c5c | L | | GOL\|TK4 | 0.98 | | 0.62 | | 0.73 | | 0.99 | | 0.66 | | 0.67 | | 8 | | 378 | | 5 | | 3 | | 3 | | 1 | |  |
| 1c5x | B | | FLC\|ESI | 0.96 | | 0.84 | | 0.73 | | 0.99 | | 0.76 | | 0.78 | | 16 | | 206 | | 3 | | 6 | | 1 | | 1 | |  |
| 1c83 | A | | OAI | 0.99 | | 0.92 | | 0.92 | | 1.00 | | 0.91 | | 0.92 | | 11 | | 248 | | 1 | | 1 | | 1 | | 1 | |  |
| 1cbs | A | | REA | 0.93 | | 0.29 | | 0.29 | | 0.96 | | 0.25 | | 0.29 | | 2 | | 122 | | 5 | | 5 | | 1 | | 1 | |  |
| 1cbx | A | | BZS | 0.93 | | 0.39 | | 0.92 | | 0.94 | | 0.58 | | 0.55 | | 11 | | 243 | | 17 | | 1 | | 2 | | 1 | |  |
| 1cdg | A | | MAL | 0.93 | | 0.00 | | 0.00 | | 0.97 | | 0.04 | | 0.00 | | 0 | | 572 | | 18 | | 24 | | 2 | | 0 | |  |
| 1ckp | A | | EDO\|PVB | 0.98 | | 0.90 | | 0.64 | | 1.00 | | 0.75 | | 0.75 | | 9 | | 234 | | 1 | | 5 | | 1 | | 1 | |  |
| 1cla | A | | CLM | 0.94 | | 0.00 | | 0.00 | | 0.97 | | 0.03 | | 0.00 | | 0 | | 170 | | 5 | | 5 | | 2 | | 0 | |  |
| 1cle | A | | NAG\|CLL\|PO4 | 0.94 | | 0.71 | | 0.42 | | 0.99 | | 0.52 | | 0.53 | | 15 | | 437 | | 6 | | 21 | | 1 | | 1 | |  |
| 1coy | A | | AND\|FAD | 0.96 | | 0.76 | | 0.88 | | 0.97 | | 0.79 | | 0.81 | | 37 | | 376 | | 12 | | 5 | | 2 | | 1 | |  |
| 1cps | A | | CPM | 0.93 | | 0.30 | | 0.70 | | 0.94 | | 0.43 | | 0.42 | | 7 | | 247 | | 16 | | 3 | | 2 | | 1 | |  |
| 1cqp | A | | MG\|803 | 0.94 | | 0.75 | | 0.25 | | 0.99 | | 0.41 | | 0.38 | | 3 | | 151 | | 1 | | 9 | | 1 | | 1 | |  |
| 1ctr | A | | TFP | 0.00 | | 0.00 | | 0.00 | | 0.00 | | 0.00 | | 0.00 | | 0 | | 119 | | 0 | | 21 | | 0 | | 0 | |  |
| 1ctt | A | | DHZ | 0.98 | | 0.67 | | 0.57 | | 0.99 | | 0.61 | | 0.62 | | 4 | | 259 | | 2 | | 3 | | 1 | | 1 | |  |
| 1d0l | A | | BLG | 0.92 | | 0.25 | | 0.27 | | 0.95 | | 0.21 | | 0.26 | | 4 | | 248 | | 12 | | 11 | | 3 | | 1 | |  |
| 1d3h | A | | ACT\|FMN\|A26\|ORO | 0.94 | | 0.79 | | 0.79 | | 0.97 | | 0.76 | | 0.79 | | 34 | | 265 | | 9 | | 9 | | 3 | | 1 | |  |
| 1dbb | H,L | | STR | 0.98 | | 0.46 | | 0.63 | | 0.99 | | 0.52 | | 0.53 | | 5 | | 397 | | 6 | | 3 | | 2 | | 1 | |  |
| 1dd7 | A | | HEM\|1PM | 0.95 | | 0.68 | | 0.65 | | 0.98 | | 0.64 | | 0.67 | | 13 | | 249 | | 6 | | 7 | | 2 | | 1 | |  |
| 1dg5 | A | | NDP\|GOL\|TOP | 0.94 | | 0.96 | | 0.74 | | 0.99 | | 0.81 | | 0.84 | | 23 | | 112 | | 1 | | 8 | | 2 | | 1 | |  |
| 1dhf | A | | FOL | 0.90 | | 0.42 | | 0.53 | | 0.93 | | 0.42 | | 0.47 | | 8 | | 145 | | 11 | | 7 | | 1 | | 1 | |  |
| 1did | A | | DIG\|MN | 0.98 | | 0.59 | | 0.91 | | 0.98 | | 0.72 | | 0.71 | | 10 | | 330 | | 7 | | 1 | | 2 | | 1 | |  |
| 1dih | A | | NDP | 0.89 | | 0.33 | | 0.39 | | 0.94 | | 0.30 | | 0.36 | | 7 | | 204 | | 14 | | 11 | | 2 | | 1 | |  |
| 1dmp | A,B | | 450\|CSO | 0.92 | | 0.90 | | 0.57 | | 0.99 | | 0.67 | | 0.69 | | 17 | | 154 | | 2 | | 13 | | 1 | | 1 | |  |
| 1dog | A | | AS2\|NOJ\|AS1\|GLC | 0.87 | | 0.90 | | 0.14 | | 1.00 | | 0.33 | | 0.24 | | 9 | | 361 | | 1 | | 55 | | 1 | | 1 | |  |
| 1dr1 | A | | CA\|BIO\|NAP | 0.93 | | 0.90 | | 0.63 | | 0.99 | | 0.71 | | 0.74 | | 17 | | 141 | | 2 | | 10 | | 2 | | 1 | |  |
| 1e96 | A,B | | GTP | 0.95 | | 0.58 | | 0.91 | | 0.95 | | 0.70 | | 0.70 | | 19 | | 290 | | 14 | | 2 | | 2 | | 1 | |  |
| 1eap | A,B | | HEP | 0.98 | | 0.73 | | 0.62 | | 0.99 | | 0.66 | | 0.67 | | 8 | | 387 | | 3 | | 5 | | 2 | | 1 | |  |
| 1ebg | A | | MG\|PAH | 0.97 | | 1.00 | | 0.21 | | 1.00 | | 0.46 | | 0.35 | | 3 | | 371 | | 0 | | 11 | | 1 | | 1 | |  |
| 1eed | P | | BOC\|CHS\|FOG | 0.96 | | 0.63 | | 0.67 | | 0.98 | | 0.63 | | 0.65 | | 10 | | 281 | | 6 | | 5 | | 1 | | 1 | |  |
| 1ei1 | A | | ANP\|GOL | 0.99 | | 0.87 | | 0.91 | | 0.99 | | 0.88 | | 0.89 | | 20 | | 329 | | 3 | | 2 | | 1 | | 1 | |  |
| 1ejn | A | | AGB | 0.97 | | 0.74 | | 0.88 | | 0.98 | | 0.79 | | 0.80 | | 14 | | 209 | | 5 | | 2 | | 2 | | 1 | |  |
| 1ela | A | | ISO\|TFA\|ACY | 0.97 | | 0.82 | | 0.69 | | 0.99 | | 0.74 | | 0.75 | | 9 | | 208 | | 2 | | 4 | | 1 | | 1 | |  |
| 1eoc | A,B | | 4NC | 0.98 | | 0.65 | | 0.92 | | 0.98 | | 0.76 | | 0.76 | | 11 | | 380 | | 6 | | 1 | | 3 | | 1 | |  |
| 1epb | A | | REA | 0.94 | | 0.68 | | 0.88 | | 0.95 | | 0.74 | | 0.77 | | 15 | | 123 | | 7 | | 2 | | 1 | | 1 | |  |
| 1eta | 1 | | T44 | 0.00 | | 0.00 | | 0.00 | | 0.00 | | 0.00 | | 0.00 | | 0 | | 114 | | 0 | | 6 | | 0 | | 0 | |  |
| 1exw | A | | HSF\|NAG | 0.90 | | 0.43 | | 0.27 | | 0.96 | | 0.29 | | 0.33 | | 6 | | 213 | | 8 | | 16 | | 2 | | 1 | |  |
| 1f0r | A,B | | 815 | 0.95 | | 0.50 | | 0.64 | | 0.96 | | 0.54 | | 0.56 | | 9 | | 242 | | 9 | | 5 | | 1 | | 1 | |  |
| 1fbl | A | | CA\|HTA | 0.96 | | 1.00 | | 0.21 | | 1.00 | | 0.45 | | 0.35 | | 4 | | 316 | | 0 | | 15 | | 2 | | 1 | |  |
| 1fen | A | | AZE | 0.92 | | 0.42 | | 0.80 | | 0.93 | | 0.55 | | 0.55 | | 8 | | 150 | | 11 | | 2 | | 1 | | 1 | |  |
| 1fgi | A | | SU1 | 0.97 | | 0.53 | | 1.00 | | 0.96 | | 0.71 | | 0.69 | | 10 | | 236 | | 9 | | 0 | | 2 | | 1 | |  |
| 1fkb | A | | RAP | 0.92 | | 1.00 | | 0.39 | | 1.00 | | 0.59 | | 0.56 | | 5 | | 83 | | 0 | | 8 | | 1 | | 1 | |  |
| 1fki | A | | SB1 | 0.95 | | 1.00 | | 0.38 | | 1.00 | | 0.60 | | 0.55 | | 3 | | 87 | | 0 | | 5 | | 1 | | 1 | |  |
| 1fmo | E | | ADN | 0.93 | | 0.30 | | 0.73 | | 0.94 | | 0.44 | | 0.42 | | 8 | | 288 | | 19 | | 3 | | 3 | | 1 | |  |
| 1frp | A | | AMP\|FDP | 0.93 | | 0.70 | | 0.53 | | 0.97 | | 0.57 | | 0.60 | | 16 | | 256 | | 7 | | 14 | | 3 | | 1 | |  |
| 1glp | A | | GTS | 0.93 | | 0.33 | | 0.27 | | 0.97 | | 0.26 | | 0.30 | | 3 | | 177 | | 6 | | 8 | | 1 | | 1 | |  |
| 1gpy | A | | G6P\|PLP | 0.94 | | 0.27 | | 0.59 | | 0.95 | | 0.37 | | 0.37 | | 13 | | 716 | | 36 | | 9 | | 6 | | 1 | |  |
| 1hak | A | | K21 | 0.98 | | 0.00 | | 0.00 | | 1.00 | | 0.01 | | 0.00 | | 0 | | 285 | | 1 | | 6 | | 1 | | 0 | |  |
| 1hbv | A,B | | GAN | 0.96 | | 0.83 | | 0.79 | | 0.98 | | 0.79 | | 0.81 | | 15 | | 161 | | 3 | | 4 | | 1 | | 1 | |  |
| 1hdy | A | | PYZ\|NAD | 0.98 | | 0.85 | | 0.89 | | 0.99 | | 0.86 | | 0.87 | | 23 | | 287 | | 4 | | 3 | | 2 | | 1 | |  |
| 1hew | A | | NAG | 0.89 | | 0.00 | | 0.00 | | 0.96 | | 0.05 | | 0.00 | | 0 | | 104 | | 4 | | 9 | | 1 | | 0 | |  |
| 1hfc | A | | HAP | 0.92 | | 0.70 | | 0.47 | | 0.98 | | 0.53 | | 0.56 | | 7 | | 127 | | 3 | | 8 | | 2 | | 1 | |  |
| 1hti | B | | PGA | 0.98 | | 0.71 | | 1.00 | | 0.98 | | 0.84 | | 0.83 | | 10 | | 209 | | 4 | | 0 | | 1 | | 1 | |  |
| 1hyt | A | | BZS\|DMS | 0.93 | | 0.67 | | 0.40 | | 0.98 | | 0.48 | | 0.50 | | 10 | | 260 | | 5 | | 15 | | 1 | | 1 | |  |
| 1ibg | L | | CU\|OBN | 0.98 | | 0.60 | | 0.75 | | 0.98 | | 0.66 | | 0.67 | | 9 | | 377 | | 6 | | 3 | | 2 | | 1 | |  |
| 1icn | A | | OLA | 0.90 | | 0.37 | | 0.88 | | 0.90 | | 0.53 | | 0.52 | | 7 | | 107 | | 12 | | 1 | | 1 | | 1 | |  |
| 1ida | A,B | | QND\|PY2\|PPL\|HPB | 0.92 | | 0.60 | | 0.50 | | 0.96 | | 0.50 | | 0.55 | | 9 | | 155 | | 6 | | 9 | | 1 | | 1 | |  |
| 1imb | A | | GD\|LIP | 0.96 | | 0.67 | | 0.71 | | 0.98 | | 0.67 | | 0.69 | | 10 | | 234 | | 5 | | 4 | | 2 | | 1 | |  |
| 1inc | A | | ICL | 0.96 | | 0.67 | | 0.33 | | 0.99 | | 0.45 | | 0.44 | | 4 | | 208 | | 2 | | 8 | | 1 | | 1 | |  |
| 1ivb | A | | NAG\|ST1 | 0.94 | | 0.36 | | 0.29 | | 0.97 | | 0.29 | | 0.32 | | 5 | | 329 | | 9 | | 12 | | 4 | | 1 | |  |
| 1ivc | A | | NAG\|FUC\|MAN\|ST2 | 0.93 | | 0.00 | | 0.00 | | 0.99 | | 0.02 | | 0.00 | | 0 | | 329 | | 3 | | 21 | | 1 | | 0 | |  |
| 1jao | A | | BTP\|GM1 | 0.91 | | 0.54 | | 0.50 | | 0.95 | | 0.47 | | 0.52 | | 7 | | 121 | | 6 | | 7 | | 1 | | 1 | |  |
| 1l82 | A | | SEO | 0.00 | | 0.00 | | 0.00 | | 0.00 | | 0.00 | | 0.00 | | 0 | | 150 | | 0 | | 2 | | 0 | | 0 | |  |
| 1lah | E | | ORN | 0.94 | | 0.00 | | 0.00 | | 0.99 | | 0.02 | | 0.00 | | 0 | | 195 | | 2 | | 11 | | 1 | | 0 | |  |
| 1lcp | A | | MPD\|PLU | 0.94 | | 0.40 | | 0.29 | | 0.98 | | 0.31 | | 0.33 | | 6 | | 380 | | 9 | | 15 | | 2 | | 1 | |  |
| 1ldm | A | | OXM\|NAD | 0.94 | | 0.82 | | 0.47 | | 0.99 | | 0.59 | | 0.60 | | 14 | | 271 | | 3 | | 16 | | 1 | | 1 | |  |
| 1lgr | A | | AMP | 0.96 | | 0.50 | | 0.25 | | 0.99 | | 0.34 | | 0.33 | | 4 | | 376 | | 4 | | 12 | | 3 | | 1 | |  |
| 1lic | A | | PPI\|HDS\|CYO | 0.94 | | 0.80 | | 0.57 | | 0.98 | | 0.64 | | 0.67 | | 8 | | 112 | | 2 | | 6 | | 1 | | 1 | |  |
| 1lmo | A | | NAG | 0.00 | | 0.00 | | 0.00 | | 0.00 | | 0.00 | | 0.00 | | 0 | | 110 | | 0 | | 7 | | 0 | | 0 | |  |
| 1lpm | A | | MPA\|NAG | 0.94 | | 0.42 | | 0.44 | | 0.97 | | 0.40 | | 0.43 | | 11 | | 438 | | 15 | | 14 | | 3 | | 1 | |  |
| 1mbi | A | | IMD\|HEM | 0.00 | | 0.00 | | 0.00 | | 0.00 | | 0.00 | | 0.00 | | 0 | | 136 | | 0 | | 13 | | 0 | | 0 | |  |
| 1mfc | H,L | | GAL\|RAM\|MAN\|ABE | 0.96 | | 0.38 | | 0.80 | | 0.97 | | 0.54 | | 0.52 | | 8 | | 366 | | 13 | | 2 | | 2 | | 1 | |  |
| 1mmp | A | | RSS | 0.92 | | 0.71 | | 0.33 | | 0.99 | | 0.45 | | 0.46 | | 5 | | 129 | | 2 | | 10 | | 1 | | 1 | |  |
| 1mmq | A | | RRS | 0.91 | | 0.86 | | 0.33 | | 0.99 | | 0.50 | | 0.48 | | 6 | | 126 | | 1 | | 12 | | 1 | | 1 | |  |
| 1mrg | A | | ADN | 0.95 | | 0.44 | | 0.70 | | 0.96 | | 0.53 | | 0.54 | | 7 | | 206 | | 9 | | 3 | | 1 | | 1 | |  |
| 1mrk | A | | FMC | 0.97 | | 1.00 | | 0.50 | | 1.00 | | 0.70 | | 0.67 | | 7 | | 213 | | 0 | | 7 | | 1 | | 1 | |  |
| 1mts | A | | BX3 | 0.95 | | 0.75 | | 0.63 | | 0.98 | | 0.66 | | 0.69 | | 12 | | 184 | | 4 | | 7 | | 1 | | 1 | |  |
| 1mup | A | | CD\|TZL | 0.00 | | 0.00 | | 0.00 | | 0.00 | | 0.00 | | 0.00 | | 0 | | 127 | | 0 | | 12 | | 0 | | 0 | |  |
| 1nco | A,B | | MPD\|CHR | 0.90 | | 1.00 | | 0.19 | | 1.00 | | 0.42 | | 0.32 | | 5 | | 190 | | 0 | | 21 | | 2 | | 1 | |  |
| 1nsc | A | | NAG\|SIA | 0.94 | | 0.59 | | 0.39 | | 0.98 | | 0.44 | | 0.47 | | 10 | | 324 | | 7 | | 16 | | 5 | | 1 | |  |
| 1okl | A | | HG\|MNS | 0.97 | | 0.73 | | 0.73 | | 0.98 | | 0.71 | | 0.73 | | 11 | | 208 | | 4 | | 4 | | 2 | | 1 | |  |
| 1pbd | A | | PAB\|FAD | 0.95 | | 0.73 | | 0.77 | | 0.97 | | 0.72 | | 0.75 | | 27 | | 319 | | 10 | | 8 | | 3 | | 1 | |  |
| 1pdz | A | | PGA\|ACE | 0.98 | | 0.75 | | 0.64 | | 0.99 | | 0.68 | | 0.69 | | 9 | | 353 | | 3 | | 5 | | 1 | | 1 | |  |
| 1pgp | A | | 6PG | 0.97 | | 0.50 | | 0.77 | | 0.98 | | 0.61 | | 0.61 | | 10 | | 401 | | 10 | | 3 | | 1 | | 1 | |  |
| 1pha | A | | HEM\|PFZ | 0.00 | | 0.00 | | 0.00 | | 0.00 | | 0.00 | | 0.00 | | 0 | | 342 | | 0 | | 28 | | 0 | | 0 | |  |
| 1poc | A | | CA\|GEL | 0.92 | | 0.67 | | 0.33 | | 0.98 | | 0.44 | | 0.44 | | 4 | | 115 | | 2 | | 8 | | 1 | | 1 | |  |
| 1ppi | A | | DAF\|GLC | 0.96 | | 0.75 | | 0.48 | | 0.99 | | 0.58 | | 0.59 | | 12 | | 426 | | 4 | | 13 | | 3 | | 1 | |  |
| 1ppk | E | | DMF\|OET\|IVAN\|XYS\|PTA | 0.95 | | 0.77 | | 0.48 | | 0.99 | | 0.58 | | 0.59 | | 10 | | 272 | | 3 | | 11 | | 2 | | 1 | |  |
| 1pso | E | | STA\|IVA | 0.97 | | 0.59 | | 0.91 | | 0.98 | | 0.72 | | 0.71 | | 10 | | 280 | | 7 | | 1 | | 1 | | 1 | |  |
| 1qbr | A,B | | 638 | 0.94 | | 0.89 | | 0.62 | | 0.99 | | 0.71 | | 0.73 | | 16 | | 159 | | 2 | | 10 | | 1 | | 1 | |  |
| 1qcf | A | | PTR\|PP1 | 0.95 | | 0.60 | | 0.71 | | 0.97 | | 0.62 | | 0.65 | | 12 | | 234 | | 8 | | 5 | | 2 | | 1 | |  |
| 1qh7 | A | | PCA\|XYP | 0.93 | | 0.40 | | 0.80 | | 0.93 | | 0.53 | | 0.53 | | 8 | | 168 | | 12 | | 2 | | 1 | | 1 | |  |
| 1qpe | A | | PP2\|PTR | 0.95 | | 0.69 | | 0.61 | | 0.98 | | 0.62 | | 0.65 | | 11 | | 228 | | 5 | | 7 | | 1 | | 1 | |  |
| 1rbp | A | | RTL | 0.94 | | 0.52 | | 0.92 | | 0.94 | | 0.66 | | 0.67 | | 11 | | 148 | | 10 | | 1 | | 1 | | 1 | |  |
| 1rds | A | | GPC | 0.85 | | 0.67 | | 0.24 | | 0.98 | | 0.33 | | 0.35 | | 4 | | 80 | | 2 | | 13 | | 2 | | 1 | |  |
| 1rgk | A | | 2AM | 0.92 | | 1.00 | | 0.27 | | 1.00 | | 0.50 | | 0.43 | | 3 | | 84 | | 0 | | 8 | | 2 | | 1 | |  |
| 1rne | A | | NAG\|C60 | 0.94 | | 0.56 | | 0.53 | | 0.97 | | 0.51 | | 0.54 | | 10 | | 263 | | 8 | | 9 | | 3 | | 1 | |  |
| 1rob | A | | C2P | 0.97 | | 1.00 | | 0.63 | | 1.00 | | 0.78 | | 0.77 | | 5 | | 107 | | 0 | | 3 | | 1 | | 1 | |  |
| 1rpa | A | | NAG\|MAN\|TAR | 0.96 | | 0.56 | | 0.39 | | 0.99 | | 0.44 | | 0.46 | | 5 | | 297 | | 4 | | 8 | | 2 | | 1 | |  |
| 1rt2 | A | | TNK\|CSD | 0.97 | | 0.54 | | 0.39 | | 0.99 | | 0.44 | | 0.45 | | 7 | | 494 | | 6 | | 11 | | 3 | | 1 | |  |
| 1sln | A | | INH | 0.90 | | 0.60 | | 0.33 | | 0.97 | | 0.40 | | 0.43 | | 6 | | 131 | | 4 | | 12 | | 1 | | 1 | |  |
| 1slt | A | | GAL\|NAG\|CYO | 0.87 | | 0.43 | | 0.21 | | 0.96 | | 0.24 | | 0.29 | | 3 | | 99 | | 4 | | 11 | | 1 | | 1 | |  |
| 1snc | A | | PTP | 0.93 | | 0.57 | | 0.40 | | 0.97 | | 0.44 | | 0.47 | | 4 | | 113 | | 3 | | 6 | | 1 | | 1 | |  |
| 1sre | A | | HAB | 0.93 | | 0.69 | | 0.69 | | 0.96 | | 0.65 | | 0.69 | | 9 | | 95 | | 4 | | 4 | | 2 | | 1 | |  |
| 1stp | A | | BTN | 0.96 | | 0.92 | | 0.73 | | 0.99 | | 0.80 | | 0.82 | | 11 | | 100 | | 1 | | 4 | | 1 | | 1 | |  |
| 1tdb | A | | UFP | 0.96 | | 0.41 | | 0.78 | | 0.97 | | 0.55 | | 0.54 | | 7 | | 276 | | 10 | | 2 | | 1 | | 1 | |  |
| 1thl | A | | CCM\|CLT | 0.95 | | 0.75 | | 0.45 | | 0.99 | | 0.56 | | 0.56 | | 9 | | 263 | | 3 | | 11 | | 1 | | 1 | |  |
| 1tlc | A | | F89\|DGP\|CBX | 0.93 | | 0.79 | | 0.44 | | 0.99 | | 0.56 | | 0.56 | | 11 | | 213 | | 3 | | 14 | | 2 | | 1 | |  |
| 1tng | A | | AMC | 0.94 | | 0.50 | | 0.69 | | 0.95 | | 0.56 | | 0.58 | | 9 | | 185 | | 9 | | 4 | | 1 | | 1 | |  |
| 1tph | 1 | | PGH | 0.99 | | 0.86 | | 1.00 | | 0.99 | | 0.92 | | 0.92 | | 12 | | 214 | | 2 | | 0 | | 1 | | 1 | |  |
| 1ukz | A | | ADP\|AMP | 0.90 | | 0.65 | | 0.60 | | 0.95 | | 0.57 | | 0.63 | | 15 | | 155 | | 8 | | 10 | | 1 | | 1 | |  |
| 1ulb | A | | GUN | 0.94 | | 0.22 | | 0.67 | | 0.95 | | 0.36 | | 0.33 | | 4 | | 247 | | 14 | | 2 | | 2 | | 1 | |  |
| 1uvs | H,L | | I11 | 0.96 | | 0.62 | | 0.93 | | 0.96 | | 0.74 | | 0.74 | | 13 | | 206 | | 8 | | 1 | | 2 | | 1 | |  |
| 1vgc | A | | V36 | 0.94 | | 0.46 | | 1.00 | | 0.94 | | 0.66 | | 0.63 | | 11 | | 196 | | 13 | | 0 | | 2 | | 1 | |  |
| 1xid | A | | ASC | 0.96 | | 0.44 | | 0.92 | | 0.96 | | 0.62 | | 0.60 | | 12 | | 329 | | 15 | | 1 | | 2 | | 1 | |  |
| 1ydr | E | | TPO\|IQP\|SEP | 0.91 | | 0.44 | | 0.46 | | 0.95 | | 0.40 | | 0.45 | | 11 | | 277 | | 14 | | 13 | | 3 | | 1 | |  |
| 2aad | A | | 2GP | 0.86 | | 0.00 | | 0.00 | | 0.98 | | 0.05 | | 0.00 | | 0 | | 84 | | 2 | | 12 | | 1 | | 0 | |  |
| 2ack | A | | EDR | 0.97 | | 0.42 | | 1.00 | | 0.97 | | 0.64 | | 0.59 | | 8 | | 412 | | 11 | | 0 | | 1 | | 1 | |  |
| 2ada | A | | HPR | 0.98 | | 0.73 | | 1.00 | | 0.98 | | 0.84 | | 0.84 | | 16 | | 277 | | 6 | | 0 | | 2 | | 1 | |  |
| 2ak3 | A | | AMP | 0.91 | | 0.26 | | 0.46 | | 0.93 | | 0.30 | | 0.33 | | 5 | | 195 | | 14 | | 6 | | 2 | | 1 | |  |
| 2cmd | A | | CIT | 0.94 | | 0.38 | | 1.00 | | 0.93 | | 0.60 | | 0.55 | | 11 | | 255 | | 18 | | 0 | | 3 | | 1 | |  |
| 2cpp | A | | HEM\|CAM | 0.93 | | 0.00 | | 0.00 | | 1.00 | | 0.01 | | 0.00 | | 0 | | 343 | | 1 | | 25 | | 1 | | 0 | |  |
| 2csc | A | | MAL\|CMC | 0.96 | | 0.85 | | 0.46 | | 1.00 | | 0.61 | | 0.60 | | 11 | | 367 | | 2 | | 13 | | 2 | | 1 | |  |
| 2ctc | A | | LOF | 0.92 | | 0.26 | | 1.00 | | 0.91 | | 0.49 | | 0.41 | | 8 | | 240 | | 23 | | 0 | | 2 | | 1 | |  |
| 2er0 | I,E | | CHS\|IVA | 0.98 | | 0.75 | | 0.69 | | 0.99 | | 0.71 | | 0.72 | | 9 | | 291 | | 3 | | 4 | | 1 | | 1 | |  |
| 2fox | A | | FMN | 0.88 | | 1.00 | | 0.13 | | 1.00 | | 0.33 | | 0.22 | | 2 | | 103 | | 0 | | 14 | | 1 | | 1 | |  |
| 2gbp | A | | GLC | 0.00 | | 0.00 | | 0.00 | | 0.00 | | 0.00 | | 0.00 | | 0 | | 252 | | 0 | | 14 | | 0 | | 0 | |  |
| 2gpb | A | | GLC\|PLP | 0.95 | | 0.00 | | 0.00 | | 0.98 | | 0.03 | | 0.00 | | 0 | | 728 | | 16 | | 21 | | 4 | | 0 | |  |
| 2ifb | A | | PLM | 0.95 | | 0.50 | | 0.29 | | 0.98 | | 0.35 | | 0.36 | | 2 | | 118 | | 2 | | 5 | | 1 | | 1 | |  |
| 2msb | A,B | | NAG\|MAN | 0.00 | | 0.00 | | 0.00 | | 0.00 | | 0.00 | | 0.00 | | 0 | | 184 | | 0 | | 17 | | 0 | | 0 | |  |
| 2phh | A | | PHB\|APR | 0.95 | | 0.64 | | 0.69 | | 0.97 | | 0.64 | | 0.67 | | 18 | | 331 | | 10 | | 8 | | 2 | | 1 | |  |
| 2pk4 | A | | ACA | 0.96 | | 1.00 | | 0.57 | | 1.00 | | 0.74 | | 0.73 | | 4 | | 69 | | 0 | | 3 | | 1 | | 1 | |  |
| 2qwb | A | | NAG\|SIA\|MAN | 0.91 | | 0.25 | | 0.03 | | 0.99 | | 0.16 | | 0.17 | | 1 | | 324 | | 3 | | 30 | | 2 | | 1 | |  |
| 2sim | A | | DAN | 0.99 | | 0.79 | | 0.92 | | 0.99 | | 0.84 | | 0.85 | | 11 | | 323 | | 3 | | 1 | | 2 | | 1 | |  |
| 2sns | A | | PTP | 0.93 | | 0.75 | | 0.27 | | 0.99 | | 0.43 | | 0.40 | | 3 | | 122 | | 1 | | 8 | | 1 | | 1 | |  |
| 2tsc | A | | CB3\|UMP | 0.96 | | 0.92 | | 0.57 | | 1.00 | | 0.71 | | 0.71 | | 12 | | 220 | | 1 | | 9 | | 1 | | 1 | |  |
| 2xis | A | | XYL | 0.95 | | 0.35 | | 0.90 | | 0.95 | | 0.54 | | 0.50 | | 9 | | 325 | | 17 | | 1 | | 1 | | 1 | |  |
| 2yhx | A | | UNK\|OTG | 0.37 | | 0.50 | | 0.01 | | 0.98 | | 0.04 | | 0.03 | | 3 | | 135 | | 3 | | 234 | | 3 | | 0 | |  |
| 2ypi | A | | PGA | 0.98 | | 0.79 | | 0.92 | | 0.99 | | 0.84 | | 0.85 | | 11 | | 210 | | 3 | | 1 | | 1 | | 1 | |  |
| 3cla | A | | CLM | 0.96 | | 0.20 | | 0.20 | | 0.98 | | 0.20 | | 0.20 | | 1 | | 176 | | 4 | | 4 | | 1 | | 1 | |  |
| 3dfr | A | | NDP\|MTX | 0.90 | | 0.86 | | 0.59 | | 0.98 | | 0.66 | | 0.70 | | 19 | | 117 | | 3 | | 13 | | 1 | | 1 | |  |
| 3er3 | E | | BOC\|CAL | 0.98 | | 0.60 | | 0.90 | | 0.98 | | 0.72 | | 0.72 | | 9 | | 289 | | 6 | | 1 | | 1 | | 1 | |  |
| 3ert | A | | OHT | 0.96 | | 0.64 | | 0.58 | | 0.98 | | 0.59 | | 0.61 | | 7 | | 202 | | 4 | | 5 | | 1 | | 1 | |  |
| 3fx2 | A | | FMN | 0.93 | | 1.00 | | 0.44 | | 1.00 | | 0.64 | | 0.61 | | 7 | | 107 | | 0 | | 9 | | 1 | | 1 | |  |
| 3gch | A | | CIN | 0.95 | | 0.47 | | 0.82 | | 0.95 | | 0.60 | | 0.60 | | 9 | | 202 | | 10 | | 2 | | 2 | | 1 | |  |
| 3gpb | A | | G1P\|PLP | 0.95 | | 0.14 | | 0.08 | | 0.98 | | 0.18 | | 0.10 | | 2 | | 738 | | 12 | | 24 | | 2 | | 1 | |  |
| 3hvt | A | | NEV | 0.94 | | 0.00 | | 0.00 | | 0.96 | | 0.03 | | 0.00 | | 0 | | 436 | | 17 | | 10 | | 5 | | 0 | |  |
| 3nos | A | | HEM\|BH4\|HAR | 0.96 | | 0.81 | | 0.52 | | 0.99 | | 0.63 | | 0.63 | | 13 | | 340 | | 3 | | 12 | | 1 | | 1 | |  |
| 3ts1 | A | | TYA | 0.93 | | 0.52 | | 0.70 | | 0.95 | | 0.57 | | 0.60 | | 14 | | 231 | | 13 | | 6 | | 4 | | 1 | |  |
| 4cts | A | | OAA | 0.98 | | 0.50 | | 0.86 | | 0.99 | | 0.65 | | 0.63 | | 6 | | 395 | | 6 | | 1 | | 2 | | 1 | |  |
| 4dfr | A | | MTX | 0.91 | | 0.50 | | 0.77 | | 0.92 | | 0.57 | | 0.61 | | 10 | | 117 | | 10 | | 3 | | 2 | | 1 | |  |
| 4est | E | | ACEFPA | 0.96 | | 0.46 | | 0.56 | | 0.97 | | 0.48 | | 0.50 | | 5 | | 208 | | 6 | | 4 | | 1 | | 1 | |  |
| 4gr1 | A | | RGS\|FAD\|PO4 | 0.92 | | 0.52 | | 0.79 | | 0.93 | | 0.60 | | 0.63 | | 30 | | 362 | | 28 | | 8 | | 3 | | 1 | |  |
| 4hvp | A | | ABA\|NLE | 0.83 | | 0.18 | | 0.60 | | 0.84 | | 0.26 | | 0.27 | | 3 | | 75 | | 14 | | 2 | | 3 | | 1 | |  |
| 4lbd | A | | 961 | 0.95 | | 0.73 | | 0.65 | | 0.98 | | 0.66 | | 0.69 | | 11 | | 197 | | 4 | | 6 | | 2 | | 1 | |  |
| 4mbp | A | | GLC | 0.95 | | 0.48 | | 0.56 | | 0.97 | | 0.49 | | 0.51 | | 10 | | 320 | | 11 | | 8 | | 1 | | 1 | |  |
| 4tln | A | | LNO\|CA | 0.92 | | 0.44 | | 0.37 | | 0.97 | | 0.36 | | 0.40 | | 7 | | 250 | | 9 | | 12 | | 1 | | 1 | |  |
| 4xia | A | | MG\|SOR | 0.99 | | 0.71 | | 1.00 | | 0.99 | | 0.83 | | 0.83 | | 12 | | 339 | | 5 | | 0 | | 1 | | 1 | |  |
| 5abp | A | | GLB\|GLA | 0.00 | | 0.00 | | 0.00 | | 0.00 | | 0.00 | | 0.00 | | 0 | | 246 | | 0 | | 14 | | 0 | | 0 | |  |
| 5cpp | A | | HEM\|ADN | 0.00 | | 0.00 | | 0.00 | | 0.00 | | 0.00 | | 0.00 | | 0 | | 338 | | 0 | | 27 | | 0 | | 0 | |  |
| 5er1 | E | | CH2\|LOL\|OME | 0.96 | | 0.41 | | 0.88 | | 0.97 | | 0.59 | | 0.56 | | 7 | | 285 | | 10 | | 1 | | 1 | | 1 | |  |
| 5p21 | A | | GNP | 0.93 | | 0.73 | | 0.65 | | 0.97 | | 0.65 | | 0.69 | | 11 | | 130 | | 4 | | 6 | | 1 | | 1 | |  |
| 5p2p | A | | DHG | 0.00 | | 0.00 | | 0.00 | | 0.00 | | 0.00 | | 0.00 | | 0 | | 97 | | 0 | | 15 | | 0 | | 0 | |  |
| 6acn | A | | PCA\|TRC\|FS4 | 0.97 | | 0.20 | | 0.06 | | 0.99 | | 0.18 | | 0.09 | | 1 | | 651 | | 4 | | 16 | | 2 | | 1 | |  |
| 6cpa | A | | ZAF | 0.94 | | 0.38 | | 0.62 | | 0.95 | | 0.45 | | 0.47 | | 8 | | 251 | | 13 | | 5 | | 1 | | 1 | |  |
| 6rnt | A | | 2AM | 0.00 | | 0.00 | | 0.00 | | 0.00 | | 0.00 | | 0.00 | | 0 | | 86 | | 0 | | 12 | | 0 | | 0 | |  |
| 6rsa | A | | UVC | 0.00 | | 0.00 | | 0.00 | | 0.00 | | 0.00 | | 0.00 | | 0 | | 14 | | 0 | | 0 | | 0 | | 0 | |  |
| 7lpr | A | | BLE | 0.97 | | 0.64 | | 1.00 | | 0.97 | | 0.79 | | 0.78 | | 9 | | 161 | | 5 | | 0 | | 1 | | 1 | |  |
| 7tim | A | | PGH | 0.99 | | 0.80 | | 1.00 | | 0.99 | | 0.89 | | 0.89 | | 12 | | 206 | | 3 | | 0 | | 1 | | 1 | |  |
| 9aat | A | | PMP | 0.97 | | 0.59 | | 0.93 | | 0.98 | | 0.73 | | 0.72 | | 13 | | 348 | | 9 | | 1 | | 1 | | 1 | |  |
| 9icd | A | | NAP | 0.96 | | 0.00 | | 0.00 | | 0.99 | | 0.02 | | 0.00 | | 0 | | 339 | | 3 | | 11 | | 1 | | 0 | |  |
|  |  | | Total | 0.94 | | 0.57 | | 0.47 | | 0.98 | | 0.49 | | 0.51 | | 1669 52132 1270 1896 | | | | | | | | | | | |  |
