# Supplementary material for: Predicting Ligand Binding Sites on Protein Surfaces by 3-Dimensional Probability Density Distributions of Interacting Atoms
Source: PLoS One. 2016 Aug 11;11(8):e0160315. doi: 10.1371/journal.pone.0160315 (PMC4981321; doi:10.1371/journal.pone.0160315)
Supplement: S4 Table — The PDB ID, chain ID, and ligand name (columns 1~3) are downloaded from PDB; the prediction performances shown in columns 4~13 are defined in Eqs 5–10 of S1 Text; column 14 shows the number of LBS predicted for the corresponding protein structure (see Methods in main text); column 15 shows the number of the top one predicted LBS (see Methods in main text) for which the geometry center is within 4Å to the corresponding ligand. (DOCX) [file pone.0160315.s005.docx]

**S4 Table. ANN_BAGGING prediction accuracy benchmarks on the independent test set S198.** The PDB ID, chain ID, and ligand name (columns 1~3) are downloaded from PDB; the prediction performances shown in columns 4~13 are defined in Equations (5)~(10) of Supplementary Methods; column 14 shows the number of LBS predicted for the corresponding protein structure (see Methods in main text); column 15 shows the number of the top one predicted LBS (see Methods in main text) for which the geometry center is within 4Å to the corresponding ligand.

| PDB | Cha | Lig | Acc | Pre | Rec | Spe | Mcc | Fsc | TP | TN | FP | FN | NP | Suc |
| --- | --- | --- | --- | --- | --- | --- | --- | --- | --- | --- | --- | --- | --- | --- |
| 1azm | A | AZM | 0.975 | 0.6 | 1 | 0.974 | 0.764 | 0.75 | 9 | 225 | 6 | 0 | 1 | 1 |
| 1b3n | A | CER | 0.956 | 0.5 | 0.8 | 0.963 | 0.612 | 0.615 | 12 | 314 | 12 | 3 | 2 | 1 |
| 1bj4 | A | PLP | 0.97 | 0.5 | 0.923 | 0.971 | 0.667 | 0.649 | 12 | 407 | 12 | 1 | 1 | 1 |
| 1c1p | A | DMS | 0.908 | 0.111 | 0.4 | 0.921 | 0.175 | 0.174 | 2 | 186 | 16 | 3 | 1 | 0 |
| 1c3s | A | SHH | 0.95 | 0.417 | 0.833 | 0.955 | 0.568 | 0.556 | 10 | 294 | 14 | 2 | 1 | 1 |
| 1cb0 | A | ADE | 0.958 | 0.571 | 0.923 | 0.96 | 0.708 | 0.706 | 12 | 218 | 9 | 1 | 1 | 1 |
| 1cea | A | ACA | 0.961 | 1 | 0.571 | 1 | 0.74 | 0.727 | 4 | 70 | 0 | 3 | 1 | 1 |
| 1d2a | B | ADE | 0.948 | 0.5 | 0.857 | 0.953 | 0.631 | 0.632 | 6 | 121 | 6 | 1 | 1 | 1 |
| 1dp0 | A | DMS | 0.831 | 0.13 | 0.06 | 0.942 | 0.003 | 0.082 | 7 | 760 | 47 | 109 | 6 | 0 |
| 1dug | A | GSH | 0.931 | 0.308 | 0.4 | 0.957 | 0.315 | 0.348 | 4 | 198 | 9 | 6 | 1 | 1 |
| 1e7a | A | PFL | 0.891 | 0.125 | 0.273 | 0.918 | 0.133 | 0.171 | 6 | 470 | 42 | 16 | 6 | 0 |
| 1eem | A | GSH | 0 | 0 | 0 | 0 | 0 | 0 | 0 | 206 | 0 | 10 | 0 | 0 |
| 1f17 | A | NAI | 0.955 | 0.667 | 0.737 | 0.972 | 0.677 | 0.7 | 14 | 239 | 7 | 5 | 2 | 1 |
| 1f3a | A | GSH | 0.923 | 0.222 | 0.182 | 0.965 | 0.161 | 0.2 | 2 | 191 | 7 | 9 | 1 | 0 |
| 1f5l | A | AMR | 0.969 | 0.632 | 1 | 0.967 | 0.781 | 0.774 | 12 | 204 | 7 | 0 | 1 | 1 |
| 1fa9 | A | PLP | 0.912 | 0.132 | 0.769 | 0.914 | 0.295 | 0.225 | 10 | 703 | 66 | 3 | 4 | 1 |
| 1fcm | A | CXN | 0.977 | 0.75 | 0.692 | 0.99 | 0.709 | 0.72 | 9 | 293 | 3 | 4 | 1 | 1 |
| 1ffy | A | MRC | 0.966 | 0.39 | 0.762 | 0.971 | 0.531 | 0.516 | 16 | 829 | 25 | 5 | 6 | 1 |
| 1fj8 | A | CER | 0.96 | 0.5 | 0.786 | 0.967 | 0.608 | 0.611 | 11 | 321 | 11 | 3 | 2 | 1 |
| 1fw1 | A | GSH | 0.962 | 0.75 | 0.8 | 0.976 | 0.754 | 0.774 | 12 | 165 | 4 | 3 | 1 | 1 |
| 1fwe | C | HAE | 0.982 | 0.471 | 1 | 0.981 | 0.68 | 0.64 | 8 | 476 | 9 | 0 | 2 | 1 |
| 1gtb | A | PZQ | 0.931 | 0.077 | 0.333 | 0.94 | 0.135 | 0.125 | 1 | 187 | 12 | 2 | 1 | 0 |
| 1h0c | A | PLP | 0.974 | 0.55 | 1 | 0.973 | 0.732 | 0.71 | 11 | 329 | 9 | 0 | 1 | 1 |
| 1h7x | A | URF | 0.913 | 0.07 | 0.75 | 0.914 | 0.212 | 0.128 | 6 | 854 | 80 | 2 | 5 | 0 |
| 1hqc | A | ADE | 0.958 | 0.308 | 0.571 | 0.968 | 0.4 | 0.4 | 4 | 273 | 9 | 3 | 1 | 0 |
| 1hwk | A | 117 | 0.923 | 0 | 0 | 0.941 | -0.035 | 0 | 0 | 337 | 21 | 7 | 1 | 0 |
| 1hwl | A | FBI | 0.933 | 0 | 0 | 0.948 | -0.03 | 0 | 0 | 346 | 19 | 6 | 1 | 0 |
| 1i0z | A | NAI | 0.949 | 0.692 | 0.72 | 0.97 | 0.678 | 0.706 | 18 | 263 | 8 | 7 | 2 | 1 |
| 1ig0 | A | VIB | 0.933 | 0.059 | 0.25 | 0.943 | 0.096 | 0.095 | 1 | 265 | 16 | 3 | 2 | 0 |
| 1ig3 | A | VIB | 0 | 0 | 0 | 0 | 0 | 0 | 0 | 230 | 0 | 7 | 0 | 0 |
| 1ihi | A | IU5 | 0.915 | 0.241 | 0.778 | 0.92 | 0.404 | 0.368 | 7 | 253 | 22 | 2 | 1 | 1 |
| 1itu | A | CIL | 0.956 | 0.5 | 0.857 | 0.961 | 0.635 | 0.632 | 12 | 295 | 12 | 2 | 2 | 1 |
| 1j3j | A | CP6 | 0.904 | 0.379 | 0.846 | 0.908 | 0.527 | 0.524 | 11 | 177 | 18 | 2 | 2 | 1 |
| 1jd0 | A | AZM | 0.979 | 0.667 | 1 | 0.978 | 0.807 | 0.8 | 10 | 219 | 5 | 0 | 1 | 1 |
| 1jh8 | A | ADE | 0.926 | 0.276 | 1 | 0.924 | 0.505 | 0.432 | 8 | 255 | 21 | 0 | 1 | 1 |
| 1jr1 | A | MOA | 0.926 | 0.281 | 0.692 | 0.935 | 0.411 | 0.4 | 9 | 329 | 23 | 4 | 3 | 0 |
| 1ju6 | A | LYA | 0.947 | 0.412 | 0.636 | 0.96 | 0.486 | 0.5 | 7 | 243 | 10 | 4 | 1 | 1 |
| 1jxm | A | GAI | 0.977 | 0 | 0 | 0.986 | -0.011 | 0 | 0 | 214 | 3 | 2 | 1 | 0 |
| 1jys | A | ADE | 0.97 | 0.545 | 0.857 | 0.974 | 0.67 | 0.667 | 6 | 190 | 5 | 1 | 1 | 1 |
| 1kdk | A | DHT | 0.863 | 0 | 0 | 0.967 | -0.061 | 0 | 0 | 145 | 5 | 18 | 1 | 0 |
| 1ki2 | A | GA2 | 0.942 | 0.333 | 0.308 | 0.971 | 0.29 | 0.32 | 4 | 270 | 8 | 9 | 1 | 1 |
| 1kmv | A | DMS | 0.877 | 0.208 | 0.833 | 0.879 | 0.379 | 0.333 | 5 | 138 | 19 | 1 | 2 | 1 |
| 1kp3 | A | GAI | 0.916 | 0 | 0 | 0.925 | -0.028 | 0 | 0 | 372 | 30 | 4 | 2 | 0 |
| 1lj5 | A | DMS | 0.907 | 0 | 0 | 0.964 | -0.047 | 0 | 0 | 320 | 12 | 21 | 1 | 0 |
| 1lpd | A | ADE | 0.987 | 0.857 | 0.75 | 0.996 | 0.795 | 0.8 | 6 | 222 | 1 | 2 | 1 | 1 |
| 1ltq | A | DMS | 0.904 | 0 | 0 | 0.911 | -0.027 | 0 | 0 | 236 | 23 | 2 | 2 | 0 |
| 1lu1 | A | ADE | 0.948 | 0 | 0 | 0.973 | -0.027 | 0 | 0 | 217 | 6 | 6 | 1 | 0 |
| 1lxf | C | BEP | 0 | 0 | 0 | 0 | 0 | 0 | 0 | 76 | 0 | 12 | 0 | 0 |
| 1m17 | A | AQ4 | 0.973 | 0.733 | 0.733 | 0.986 | 0.719 | 0.733 | 11 | 278 | 4 | 4 | 1 | 1 |
| 1m2z | A | DEX | 0.921 | 0.615 | 0.364 | 0.977 | 0.435 | 0.457 | 8 | 214 | 5 | 14 | 2 | 1 |
| 1m54 | A | PLP | 0.926 | 0.389 | 0.875 | 0.928 | 0.554 | 0.538 | 14 | 285 | 22 | 2 | 2 | 1 |
| 1me7 | A | MOA | 0.918 | 0.192 | 0.5 | 0.931 | 0.275 | 0.278 | 5 | 285 | 21 | 5 | 2 | 0 |
| 1mrl | A | DOL | 0 | 0 | 0 | 0 | 0 | 0 | 0 | 183 | 0 | 8 | 0 | 0 |
| 1mud | A | ADE | 0.922 | 0.333 | 0.6 | 0.938 | 0.41 | 0.429 | 6 | 183 | 12 | 4 | 2 | 1 |
| 1n2z | A | CNC | 0.929 | 0.545 | 0.353 | 0.976 | 0.403 | 0.429 | 6 | 202 | 5 | 11 | 1 | 1 |
| 1nrg | A | PLP | 0.955 | 0 | 0 | 0.979 | -0.023 | 0 | 0 | 190 | 4 | 5 | 1 | 0 |
| 1nsi | A | ARG | 0.934 | 0.185 | 0.556 | 0.942 | 0.295 | 0.278 | 5 | 360 | 22 | 4 | 3 | 0 |
| 1oat | A | PLP | 0.978 | 0.6 | 0.818 | 0.983 | 0.69 | 0.692 | 9 | 351 | 6 | 2 | 2 | 1 |
| 1od2 | B | ADE | 0.978 | 0 | 0 | 0.988 | -0.011 | 0 | 0 | 634 | 8 | 6 | 3 | 0 |
| 1oxr | A | AIN | 0.904 | 0.429 | 0.3 | 0.962 | 0.309 | 0.353 | 3 | 101 | 4 | 7 | 2 | 1 |
| 1p0m | A | CHT | 0.955 | 0.25 | 1 | 0.954 | 0.488 | 0.4 | 7 | 438 | 21 | 0 | 2 | 1 |
| 1p5j | A | PLP | 0.95 | 0.552 | 0.941 | 0.951 | 0.699 | 0.696 | 16 | 252 | 13 | 1 | 2 | 1 |
| 1p7r | A | NCT | 0.952 | 0 | 0 | 0.983 | -0.023 | 0 | 0 | 354 | 6 | 12 | 2 | 0 |
| 1pbc | A | BHA | 0.907 | 0.211 | 0.667 | 0.915 | 0.34 | 0.32 | 8 | 324 | 30 | 4 | 2 | 0 |
| 1pj2 | A | NAI | 0.944 | 0.655 | 0.514 | 0.979 | 0.551 | 0.576 | 19 | 456 | 10 | 18 | 2 | 1 |
| 1pk2 | A | ACA | 0.847 | 0.25 | 0.222 | 0.921 | 0.151 | 0.235 | 2 | 70 | 6 | 7 | 2 | 0 |
| 1pn3 | A | DVV | 0.897 | 0.227 | 0.217 | 0.946 | 0.167 | 0.222 | 5 | 299 | 17 | 18 | 1 | 0 |
| 1px7 | A | GSH | 0.893 | 0.133 | 0.2 | 0.93 | 0.108 | 0.16 | 2 | 174 | 13 | 8 | 1 | 0 |
| 1q1c | A | DMS | 0.916 | 0.5 | 0.158 | 0.986 | 0.248 | 0.24 | 3 | 204 | 3 | 16 | 1 | 0 |
| 1q6i | A | FK5 | 0.969 | 0.875 | 0.583 | 0.995 | 0.7 | 0.7 | 7 | 182 | 1 | 5 | 1 | 1 |
| 1q8m | A | GSH | 0 | 0 | 0 | 0 | 0 | 0 | 0 | 111 | 0 | 4 | 0 | 0 |
| 1q8y | B | ADE | 0.973 | 0.5 | 0.778 | 0.978 | 0.611 | 0.609 | 7 | 312 | 7 | 2 | 2 | 1 |
| 1qb7 | A | ADE | 0.942 | 0.389 | 0.778 | 0.949 | 0.525 | 0.519 | 7 | 204 | 11 | 2 | 2 | 1 |
| 1qca | A | FUA | 0.929 | 0.25 | 0.222 | 0.966 | 0.199 | 0.235 | 2 | 169 | 6 | 7 | 1 | 0 |
| 1qci | A | ADE | 0.975 | 0.5 | 0.833 | 0.979 | 0.634 | 0.625 | 5 | 231 | 5 | 1 | 1 | 1 |
| 1qd2 | A | ADE | 0.978 | 0.643 | 1 | 0.978 | 0.793 | 0.783 | 9 | 218 | 5 | 0 | 2 | 1 |
| 1qgj | A | GSH | 0.891 | 0 | 0 | 0.911 | -0.046 | 0 | 0 | 246 | 24 | 6 | 2 | 0 |
| 1qhy | A | CLM | 0.868 | 0.409 | 0.5 | 0.913 | 0.378 | 0.45 | 9 | 136 | 13 | 9 | 1 | 1 |
| 1r4w | A | GSH | 0.914 | 0.407 | 0.917 | 0.914 | 0.577 | 0.564 | 11 | 169 | 16 | 1 | 2 | 1 |
| 1r6n | A | DMS | 0 | 0 | 0 | 0 | 0 | 0 | 0 | 181 | 0 | 2 | 0 | 0 |
| 1rbw | A | GAI | 0 | 0 | 0 | 0 | 0 | 0 | 0 | 106 | 0 | 7 | 0 | 0 |
| 1rv7 | B | AB1 | 0 | 0 | 0 | 0 | 0 | 0 | 0 | 92 | 0 | 5 | 0 | 0 |
| 1s19 | A | MC9 | 0.962 | 0.773 | 0.81 | 0.977 | 0.77 | 0.791 | 17 | 212 | 5 | 4 | 2 | 1 |
| 1s2a | A | DMS | 0.915 | 0.207 | 0.857 | 0.916 | 0.396 | 0.333 | 6 | 251 | 23 | 1 | 1 | 0 |
| 1s2d | A | ADE | 0 | 0 | 0 | 0 | 0 | 0 | 0 | 139 | 0 | 5 | 0 | 0 |
| 1s9p | A | DES | 0.956 | 0.733 | 0.688 | 0.979 | 0.686 | 0.71 | 11 | 183 | 4 | 5 | 1 | 1 |
| 1sbr | A | VIB | 0 | 0 | 0 | 0 | 0 | 0 | 0 | 149 | 0 | 21 | 0 | 0 |
| 1sqn | A | NDR | 0.905 | 0 | 0 | 0.981 | -0.039 | 0 | 0 | 209 | 4 | 18 | 1 | 0 |
| 1sxk | A | BHA | 0.94 | 0 | 0 | 0.956 | -0.028 | 0 | 0 | 109 | 5 | 2 | 1 | 0 |
| 1t69 | A | SHH | 0.953 | 0.435 | 0.909 | 0.955 | 0.61 | 0.588 | 10 | 275 | 13 | 1 | 2 | 1 |
| 1td7 | A | NFL | 0.956 | 0.818 | 0.75 | 0.98 | 0.759 | 0.783 | 9 | 99 | 2 | 3 | 2 | 1 |
| 1tgm | A | AIN | 0.948 | 0.333 | 0.2 | 0.982 | 0.233 | 0.25 | 1 | 109 | 2 | 4 | 1 | 0 |
| 1th6 | A | OIN | 0.872 | 0.333 | 0.5 | 0.907 | 0.34 | 0.4 | 5 | 97 | 10 | 5 | 1 | 1 |
| 1tpf | A | DMS | 0.924 | 0.25 | 0.444 | 0.944 | 0.297 | 0.32 | 4 | 204 | 12 | 5 | 2 | 1 |
| 1tt6 | A | DES | 0 | 0 | 0 | 0 | 0 | 0 | 0 | 90 | 0 | 5 | 0 | 0 |
| 1tuf | A | AZ1 | 0.92 | 0.162 | 0.857 | 0.921 | 0.352 | 0.273 | 6 | 363 | 31 | 1 | 3 | 0 |
| 1tz8 | A | DMS | 0 | 0 | 0 | 0 | 0 | 0 | 0 | 87 | 0 | 4 | 0 | 0 |
| 1uae | A | FCN | 0.938 | 0.267 | 0.889 | 0.939 | 0.467 | 0.41 | 8 | 338 | 22 | 1 | 1 | 1 |
| 1udu | A | CIA | 0.937 | 0.36 | 0.75 | 0.944 | 0.492 | 0.486 | 9 | 272 | 16 | 3 | 1 | 1 |
| 1umj | A | GAI | 0.925 | 0 | 0 | 0.956 | -0.039 | 0 | 0 | 86 | 4 | 3 | 1 | 0 |
| 1upf | D | URF | 0.919 | 0.25 | 0.5 | 0.937 | 0.316 | 0.333 | 4 | 178 | 12 | 4 | 1 | 0 |
| 1usq | A | CLM | 0.897 | 0 | 0 | 0.953 | -0.054 | 0 | 0 | 122 | 6 | 8 | 1 | 0 |
| 1uw6 | A | NCT | 0 | 0 | 0 | 0 | 0 | 0 | 0 | 196 | 0 | 6 | 0 | 0 |
| 1w0g | A | MYT | 0.933 | 0.176 | 0.857 | 0.934 | 0.371 | 0.293 | 6 | 399 | 28 | 1 | 3 | 0 |
| 1w6f | A | ISZ | 0.956 | 0.4 | 0.75 | 0.963 | 0.528 | 0.522 | 6 | 234 | 9 | 2 | 1 | 1 |
| 1w6r | A | GNT | 0.98 | 0.619 | 0.929 | 0.981 | 0.749 | 0.743 | 13 | 419 | 8 | 1 | 1 | 1 |
| 1wap | A | TRP | 0 | 0 | 0 | 0 | 0 | 0 | 0 | 55 | 0 | 11 | 0 | 0 |
| 1x8v | A | ESL | 0.922 | 0.276 | 0.444 | 0.945 | 0.311 | 0.34 | 8 | 359 | 21 | 10 | 3 | 0 |
| 1xcl | A | GAI | 0.875 | 0.188 | 1 | 0.871 | 0.404 | 0.316 | 6 | 176 | 26 | 0 | 1 | 0 |
| 1xe8 | A | ADE | 0.927 | 0.375 | 0.667 | 0.94 | 0.465 | 0.48 | 6 | 158 | 10 | 3 | 1 | 1 |
| 1xj7 | A | DHT | 0.9 | 0.091 | 0.062 | 0.957 | 0.023 | 0.074 | 1 | 223 | 10 | 15 | 2 | 0 |
| 1y4l | B | SVR | 0.885 | 0.455 | 0.417 | 0.941 | 0.371 | 0.435 | 5 | 95 | 6 | 7 | 2 | 0 |
| 1y8e | A | SVR | 0.959 | 0 | 0 | 0.987 | -0.019 | 0 | 0 | 233 | 3 | 7 | 1 | 0 |
| 1yhm | A | AHD | 0.961 | 0.409 | 1 | 0.96 | 0.627 | 0.581 | 9 | 310 | 13 | 0 | 1 | 1 |
| 1yki | B | DMS | 0.961 | 0 | 0 | 0.975 | -0.019 | 0 | 0 | 196 | 5 | 3 | 1 | 0 |
| 1yxm | B | ADE | 0.898 | 0.214 | 0.667 | 0.906 | 0.339 | 0.324 | 6 | 213 | 22 | 3 | 3 | 0 |
| 1z11 | A | 8MO | 0 | 0 | 0 | 0 | 0 | 0 | 0 | 416 | 0 | 11 | 0 | 0 |
| 1zmd | A | NAI | 0.894 | 0.25 | 0.65 | 0.905 | 0.359 | 0.361 | 13 | 373 | 39 | 7 | 2 | 0 |
| 1zn7 | A | ADE | 0.894 | 0.2 | 0.429 | 0.917 | 0.243 | 0.273 | 3 | 132 | 12 | 4 | 1 | 0 |
| 2ab2 | A | SNL | 0.901 | 0 | 0 | 0.981 | -0.039 | 0 | 0 | 210 | 4 | 19 | 1 | 0 |
| 2abj | A | PLP | 0.929 | 0.361 | 0.929 | 0.929 | 0.554 | 0.52 | 13 | 301 | 23 | 1 | 2 | 1 |
| 2agd | A | NAG | 0.929 | 0.375 | 0.818 | 0.934 | 0.525 | 0.514 | 9 | 214 | 15 | 2 | 1 | 1 |
| 2aou | A | CQA | 0.886 | 0.433 | 0.542 | 0.923 | 0.422 | 0.481 | 13 | 205 | 17 | 11 | 3 | 1 |
| 2azx | A | TRP | 0.945 | 0.533 | 0.727 | 0.959 | 0.595 | 0.615 | 16 | 327 | 14 | 6 | 2 | 1 |
| 2bdm | A | TMI | 0.901 | 0.364 | 0.343 | 0.949 | 0.299 | 0.353 | 12 | 387 | 21 | 23 | 2 | 1 |
| 2biu | X | DMS | 0.919 | 1 | 0.294 | 1 | 0.519 | 0.455 | 5 | 132 | 0 | 12 | 1 | 1 |
| 2bxg | A | IBP | 0.906 | 0.162 | 0.273 | 0.935 | 0.163 | 0.203 | 6 | 449 | 31 | 16 | 4 | 0 |
| 2bxn | A | IDB | 0.886 | 0.119 | 0.467 | 0.898 | 0.192 | 0.189 | 7 | 458 | 52 | 8 | 5 | 0 |
| 2c6g | A | GLU | 0.939 | 0.244 | 1 | 0.938 | 0.479 | 0.393 | 11 | 513 | 34 | 0 | 3 | 1 |
| 2c6n | A | LPR | 0.939 | 0.31 | 0.867 | 0.942 | 0.497 | 0.456 | 13 | 467 | 29 | 2 | 4 | 1 |
| 2cev | A | GAI | 0.902 | 0 | 0 | 0.932 | -0.049 | 0 | 0 | 220 | 16 | 8 | 1 | 0 |
| 2cft | A | PLP | 0.953 | 0.429 | 1 | 0.951 | 0.638 | 0.6 | 9 | 232 | 12 | 0 | 3 | 1 |
| 2ch5 | A | NAG | 0.927 | 0.371 | 1 | 0.924 | 0.586 | 0.542 | 13 | 266 | 22 | 0 | 1 | 1 |
| 2coj | A | GBN | 0.936 | 0.323 | 1 | 0.934 | 0.549 | 0.488 | 10 | 298 | 21 | 0 | 1 | 1 |
| 2f2q | A | GAI | 0.945 | 0 | 0 | 0.981 | -0.026 | 0 | 0 | 156 | 3 | 6 | 1 | 0 |
| 2f89 | F | 210 | 0.943 | 0.333 | 1 | 0.941 | 0.56 | 0.5 | 9 | 287 | 18 | 0 | 1 | 1 |
| 2f8z | F | ZOL | 0.938 | 0.355 | 1 | 0.935 | 0.576 | 0.524 | 11 | 289 | 20 | 0 | 2 | 1 |
| 2f92 | F | AHD | 0.943 | 0.357 | 1 | 0.941 | 0.58 | 0.526 | 10 | 286 | 18 | 0 | 1 | 1 |
| 2fy3 | A | CHT | 0.96 | 0.241 | 1 | 0.96 | 0.481 | 0.389 | 7 | 523 | 22 | 0 | 4 | 1 |
| 2gsk | A | CNC | 0.889 | 0.254 | 0.586 | 0.906 | 0.336 | 0.354 | 17 | 482 | 50 | 12 | 6 | 0 |
| 2h79 | A | T3 | 0.963 | 0.609 | 1 | 0.961 | 0.765 | 0.757 | 14 | 223 | 9 | 0 | 1 | 1 |
| 2h9t | H | SVR | 0.842 | 0 | 0 | 0.886 | -0.08 | 0 | 0 | 186 | 24 | 11 | 1 | 0 |
| 2hgs | A | GSH | 0.94 | 0.371 | 0.765 | 0.947 | 0.507 | 0.5 | 13 | 392 | 22 | 4 | 2 | 1 |
| 2ht9 | A | GSH | 0.958 | 0.75 | 0.75 | 0.977 | 0.727 | 0.75 | 6 | 85 | 2 | 2 | 1 | 1 |
| 2hzp | A | PLP | 0.975 | 0.5 | 1 | 0.975 | 0.698 | 0.667 | 10 | 384 | 10 | 0 | 1 | 1 |
| 2j6l | A | NAI | 0.957 | 0.605 | 0.852 | 0.964 | 0.697 | 0.708 | 23 | 397 | 15 | 4 | 3 | 1 |
| 2jai | A | CIR | 0.961 | 0.8 | 0.533 | 0.991 | 0.635 | 0.64 | 8 | 214 | 2 | 7 | 1 | 1 |
| 2jhf | A | DMS | 0.92 | 0.226 | 0.778 | 0.924 | 0.392 | 0.35 | 7 | 291 | 24 | 2 | 2 | 0 |
| 2jis | A | PLP | 0.977 | 0.6 | 1 | 0.977 | 0.765 | 0.75 | 15 | 416 | 10 | 0 | 2 | 1 |
| 2nvu | B | ATP | 0.969 | 0.4 | 0.706 | 0.976 | 0.518 | 0.511 | 12 | 719 | 18 | 5 | 4 | 1 |
| 2nyr | B | SVR | 0.924 | 0.533 | 0.4 | 0.969 | 0.422 | 0.457 | 8 | 222 | 7 | 12 | 1 | 1 |
| 2nz2 | A | ASP\|CIR | 0.929 | 0.368 | 0.875 | 0.931 | 0.54 | 0.519 | 14 | 324 | 24 | 2 | 3 | 1 |
| 2obv | A | SAM | 0.955 | 0.286 | 0.444 | 0.969 | 0.334 | 0.348 | 4 | 314 | 10 | 5 | 2 | 0 |
| 2of1 | A | DMS | 0.927 | 0 | 0 | 0.958 | -0.038 | 0 | 0 | 114 | 5 | 4 | 1 | 0 |
| 2oo0 | A | PLP | 0.946 | 0.424 | 0.933 | 0.947 | 0.609 | 0.583 | 14 | 339 | 19 | 1 | 3 | 1 |
| 2p8n | A | ADE | 0.967 | 0.533 | 0.889 | 0.97 | 0.674 | 0.667 | 8 | 230 | 7 | 1 | 1 | 1 |
| 2qr2 | B | VK3 | 0 | 0 | 0 | 0 | 0 | 0 | 0 | 213 | 0 | 5 | 0 | 0 |
| 2qxs | A | RAL | 0.929 | 1 | 0.348 | 1 | 0.568 | 0.516 | 8 | 188 | 0 | 15 | 1 | 1 |
| 2vcq | A | GSH | 0.93 | 0.333 | 0.3 | 0.966 | 0.279 | 0.316 | 3 | 169 | 6 | 7 | 1 | 0 |
| 2vdm | B | AGG | 0 | 0 | 0 | 0 | 0 | 0 | 0 | 416 | 0 | 9 | 0 | 0 |
| 2w8y | A | 486 | 0.898 | 0.375 | 0.136 | 0.977 | 0.181 | 0.2 | 3 | 208 | 5 | 19 | 2 | 0 |
| 2xat | A | CLM | 0 | 0 | 0 | 0 | 0 | 0 | 0 | 181 | 0 | 5 | 0 | 0 |
| 2xh1 | A | PLP | 0.972 | 0.667 | 0.429 | 0.992 | 0.521 | 0.522 | 6 | 378 | 3 | 8 | 1 | 1 |
| 2xhd | A | GLU | 0 | 0 | 0 | 0 | 0 | 0 | 0 | 233 | 0 | 9 | 0 | 0 |
| 2zt7 | A | GLY | 0.941 | 0.171 | 1 | 0.94 | 0.401 | 0.293 | 6 | 456 | 29 | 0 | 3 | 0 |
| 3b6r | B | CRN | 0.939 | 0.231 | 0.857 | 0.941 | 0.426 | 0.364 | 6 | 317 | 20 | 1 | 2 | 1 |
| 3b9m | A | SAL | 0.849 | 0.089 | 0.667 | 0.853 | 0.205 | 0.157 | 8 | 475 | 82 | 4 | 6 | 0 |
| 3ba0 | A | HAE | 0.95 | 0.111 | 0.5 | 0.955 | 0.219 | 0.182 | 2 | 337 | 16 | 2 | 3 | 0 |
| 3bg3 | A | PYR | 0.939 | 0.156 | 1 | 0.939 | 0.382 | 0.269 | 7 | 580 | 38 | 0 | 4 | 1 |
| 3bju | A | LYS | 0.932 | 0.235 | 0.571 | 0.943 | 0.338 | 0.333 | 8 | 433 | 26 | 6 | 4 | 1 |
| 3c6m | A | SPM | 0.941 | 0.409 | 0.6 | 0.958 | 0.466 | 0.486 | 9 | 296 | 13 | 6 | 2 | 1 |
| 3cfq | A | DIF | 0 | 0 | 0 | 0 | 0 | 0 | 0 | 101 | 0 | 8 | 0 | 0 |
| 3cla | A | CLM | 0.941 | 0.429 | 0.3 | 0.977 | 0.328 | 0.353 | 3 | 171 | 4 | 7 | 1 | 0 |
| 3cog | A | PLP | 0.976 | 0.632 | 0.923 | 0.978 | 0.752 | 0.75 | 12 | 307 | 7 | 1 | 1 | 1 |
| 3czd | A | GLU | 0.923 | 0.333 | 0.909 | 0.924 | 0.524 | 0.488 | 10 | 243 | 20 | 1 | 2 | 1 |
| 3d90 | A | NOG | 0.905 | 0 | 0 | 0.981 | -0.038 | 0 | 0 | 210 | 4 | 18 | 1 | 0 |
| 3dgq | A | EAA | 0.933 | 0.286 | 0.571 | 0.947 | 0.373 | 0.381 | 4 | 177 | 10 | 3 | 1 | 1 |
| 3dk8 | A | GSH | 0.89 | 0.164 | 0.9 | 0.889 | 0.356 | 0.277 | 9 | 370 | 46 | 1 | 4 | 0 |
| 3dyd | A | PLP | 0.923 | 0.297 | 0.846 | 0.926 | 0.474 | 0.44 | 11 | 326 | 26 | 2 | 2 | 1 |
| 3dzy | D | BRL | 0.968 | 0.733 | 0.611 | 0.988 | 0.653 | 0.667 | 11 | 318 | 4 | 7 | 1 | 1 |
| 3e77 | A | PLP | 0.958 | 0.481 | 1 | 0.956 | 0.678 | 0.65 | 13 | 303 | 14 | 0 | 1 | 1 |
| 3erd | A | DES | 0 | 0 | 0 | 0 | 0 | 0 | 0 | 198 | 0 | 15 | 0 | 0 |
| 3ert | A | OHT | 0.954 | 0.857 | 0.6 | 0.99 | 0.695 | 0.706 | 12 | 196 | 2 | 8 | 1 | 1 |
| 3fhx | A | PXL | 0.925 | 0.2 | 1 | 0.924 | 0.43 | 0.333 | 5 | 242 | 20 | 0 | 2 | 1 |
| 3gmz | A | ORN | 0.982 | 0.667 | 0.889 | 0.985 | 0.761 | 0.762 | 8 | 259 | 4 | 1 | 1 | 1 |
| 3gwx | A | EPA | 0.931 | 0.667 | 0.615 | 0.966 | 0.602 | 0.64 | 16 | 226 | 8 | 10 | 2 | 1 |
| 3h6t | A | CYZ | 0.94 | 0 | 0 | 0.975 | -0.03 | 0 | 0 | 234 | 6 | 9 | 1 | 0 |
| 3ii0 | A | PLP | 0.936 | 0.324 | 1 | 0.934 | 0.55 | 0.489 | 11 | 324 | 23 | 0 | 2 | 1 |
| 3inj | A | GAI | 0.895 | 0 | 0 | 0.904 | -0.031 | 0 | 0 | 394 | 42 | 4 | 2 | 0 |
| 3iyt | A | ATP | 0.962 | 0.417 | 0.667 | 0.971 | 0.509 | 0.513 | 10 | 465 | 14 | 5 | 3 | 1 |
| 3jdw | A | ORN | 0.988 | 0.857 | 0.667 | 0.997 | 0.75 | 0.75 | 6 | 310 | 1 | 3 | 1 | 1 |
| 3k4v | A | DMS | 0 | 0 | 0 | 0 | 0 | 0 | 0 | 87 | 0 | 7 | 0 | 0 |
| 3kvv | A | URF | 0.967 | 0.611 | 1 | 0.965 | 0.768 | 0.759 | 11 | 191 | 7 | 0 | 1 | 1 |
| 3l6b | A | PLP | 0.936 | 0.333 | 0.133 | 0.984 | 0.182 | 0.19 | 2 | 246 | 4 | 13 | 2 | 0 |
| 3pah | A | ALE | 0.965 | 0.4 | 0.857 | 0.967 | 0.572 | 0.545 | 6 | 267 | 9 | 1 | 2 | 1 |
| 4pah | A | LNR | 0.961 | 0.353 | 1 | 0.96 | 0.582 | 0.522 | 6 | 267 | 11 | 0 | 2 | 1 |
| 5jdw | A | GLY | 0.963 | 0.333 | 1 | 0.962 | 0.566 | 0.5 | 6 | 304 | 12 | 0 | 2 | 1 |
| 6pah | A | DAH | 0.944 | 0.304 | 1 | 0.943 | 0.536 | 0.467 | 7 | 264 | 16 | 0 | 3 | 1 |
|  |  | Total | 0.936 | 0.324 | 0.528 | 0.953 | 0.382 | 0.402 | 1254 | 53530 | 2614 | 1123 |  |  |
