# Supplementary material for: Predicting Ligand Binding Sites on Protein Surfaces by 3-Dimensional Probability Density Distributions of Interacting Atoms
Source: PLoS One. 2016 Aug 11;11(8):e0160315. doi: 10.1371/journal.pone.0160315 (PMC4981321; doi:10.1371/journal.pone.0160315)
Supplement: S5 Table — The PDB ID, chain ID, and ligand name (columns 1~3) are downloaded from PDB; the prediction performances shown in columns 4~13 are defined in Eqs 5–10 of S1 Text; column 14 shows the number of actual LBSs for the corresponding complex structure; column 15 shows the number of LBS predicted for the corresponding protein structure (see Methods in main text);. (DOCX) [file pone.0160315.s006.docx]

**S5 Table. ANN_BAGGING prediction accuracy benchmarks on the independent test set S523.** The PDB ID, chain ID, and ligand name (columns 1~3) are downloaded from PDB; the prediction performances shown in columns 4~13 are defined in Equations (5)~(10) of Supplementary Methods; column 14 shows the number of actual LBSs for the corresponding complex structure; column 15 shows the number of LBS predicted for the corresponding protein structure (see Methods in main text);.

| PDB | Cha | Lig | Acc | Pre | Rec | Spe | Mcc | Fsc | TP | TN | FP | FN | NRS | NPS |
| --- | --- | --- | --- | --- | --- | --- | --- | --- | --- | --- | --- | --- | --- | --- |
| 13gs | A | SAS | 0.938 | 0.417 | 0.5 | 0.962 | 0.424 | 0.455 | 5 | 178 | 7 | 5 | 1 | 1 |
| 148l | E | DAL | 0 | 0 | 0 | 0 | 0 | 0 | 0 | 154 | 0 | 0 | 1 | 0 |
| 1a26 | A | CNA | 0.944 | 0 | 0 | 0.968 | -0.029 | 0 | 0 | 304 | 10 | 8 | 1 | 1 |
| 1a2k | C | GDP | 0.971 | 0.857 | 0.8 | 0.987 | 0.812 | 0.828 | 12 | 156 | 2 | 3 | 1 | 1 |
| 1a42 | A | BZU | 0.952 | 0.636 | 0.824 | 0.962 | 0.699 | 0.718 | 14 | 202 | 8 | 3 | 1 | 2 |
| 1a4k | H | FRA | 0.967 | 0.667 | 0.444 | 0.99 | 0.528 | 0.533 | 4 | 198 | 2 | 5 | 1 | 1 |
| 1a7x | A | FKA | 0.903 | 0 | 0 | 0.966 | -0.048 | 0 | 0 | 84 | 3 | 6 | 1 | 1 |
| 1a8t | A | 61 | 0.947 | 0.643 | 0.6 | 0.974 | 0.593 | 0.621 | 9 | 188 | 5 | 6 | 1 | 2 |
| 1afk | A | PAP | 0.938 | 1 | 0.5 | 1 | 0.683 | 0.667 | 7 | 99 | 0 | 7 | 1 | 1 |
| 1arc | A | TCK | 0.94 | 0.429 | 0.5 | 0.964 | 0.431 | 0.462 | 6 | 213 | 8 | 6 | 1 | 2 |
| 1azm | A | AZM | 0.983 | 0.733 | 1 | 0.983 | 0.849 | 0.846 | 11 | 225 | 4 | 0 | 1 | 1 |
| 1b8u | A | OAA | 0.903 | 0.125 | 0.3 | 0.924 | 0.149 | 0.176 | 3 | 257 | 21 | 7 | 2 | 2 |
| 1b8u | A | NAD | 0.958 | 0.708 | 0.773 | 0.974 | 0.717 | 0.739 | 17 | 259 | 7 | 5 | 2 | 2 |
| 1blc | A | TEM | 0.969 | 0.714 | 0.5 | 0.991 | 0.582 | 0.588 | 5 | 213 | 2 | 5 | 2 | 1 |
| 1blc | A | CEM | 0.973 | 0.714 | 0.556 | 0.991 | 0.617 | 0.625 | 5 | 214 | 2 | 4 | 2 | 1 |
| 1bnw | A | TPD | 0.961 | 0.611 | 0.846 | 0.968 | 0.7 | 0.71 | 11 | 209 | 7 | 2 | 1 | 2 |
| 1bq3 | A | IHP | 0.926 | 0.533 | 0.471 | 0.965 | 0.461 | 0.5 | 8 | 193 | 7 | 9 | 1 | 2 |
| 1bqo | B | N25 | 0.938 | 0.875 | 0.438 | 0.993 | 0.593 | 0.583 | 7 | 144 | 1 | 9 | 1 | 1 |
| 1br6 | A | PT1 | 0.984 | 0.846 | 0.846 | 0.992 | 0.838 | 0.846 | 11 | 234 | 2 | 2 | 1 | 1 |
| 1bs1 | A | ADP | 0.929 | 0.562 | 0.562 | 0.961 | 0.524 | 0.562 | 9 | 173 | 7 | 7 | 2 | 2 |
| 1bs1 | A | DAA | 0.939 | 0.625 | 0.625 | 0.967 | 0.592 | 0.625 | 10 | 174 | 6 | 6 | 2 | 2 |
| 1bsv | A | NDP | 0.931 | 0.577 | 0.625 | 0.959 | 0.563 | 0.6 | 15 | 255 | 11 | 9 | 1 | 2 |
| 1bxq | A | PP8 | 0.959 | 0.882 | 0.6 | 0.993 | 0.708 | 0.714 | 15 | 269 | 2 | 10 | 1 | 2 |
| 1bzy | A | POP | 0.912 | 0.25 | 0.444 | 0.935 | 0.29 | 0.32 | 4 | 172 | 12 | 5 | 2 | 1 |
| 1bzy | A | IMU | 0.964 | 0.812 | 0.765 | 0.983 | 0.768 | 0.788 | 13 | 173 | 3 | 4 | 2 | 1 |
| 1c1h | A | MMP | 0.935 | 0.542 | 0.65 | 0.957 | 0.558 | 0.591 | 13 | 244 | 11 | 7 | 1 | 1 |
| 1c3j | A | UDP | 0.951 | 0.636 | 0.389 | 0.986 | 0.474 | 0.483 | 7 | 285 | 4 | 11 | 1 | 1 |
| 1cen | A | BGC | 0.962 | 0.538 | 0.583 | 0.978 | 0.54 | 0.56 | 7 | 269 | 6 | 5 | 1 | 2 |
| 1cet | A | CLQ | 0.948 | 0.368 | 0.778 | 0.954 | 0.514 | 0.5 | 7 | 249 | 12 | 2 | 1 | 2 |
| 1ch8 | A | GPX | 0.923 | 0.312 | 0.556 | 0.941 | 0.379 | 0.4 | 10 | 348 | 22 | 8 | 3 | 1 |
| 1ch8 | A | HDA | 0.938 | 0.281 | 0.9 | 0.939 | 0.483 | 0.429 | 9 | 355 | 23 | 1 | 3 | 1 |
| 1ch8 | A | IMP | 0.943 | 0.469 | 0.75 | 0.954 | 0.566 | 0.577 | 15 | 351 | 17 | 5 | 3 | 1 |
| 1cim | A | PTS | 0.953 | 0.571 | 0.857 | 0.959 | 0.677 | 0.686 | 12 | 209 | 9 | 2 | 1 | 2 |
| 1d3p | B | BT3 | 0.953 | 0.682 | 0.789 | 0.967 | 0.708 | 0.732 | 15 | 206 | 7 | 4 | 1 | 1 |
| 1d4p | B | BPP | 0.943 | 0.619 | 0.722 | 0.962 | 0.638 | 0.667 | 13 | 202 | 8 | 5 | 1 | 2 |
| 1dcp | A | HBI | 0 | 0 | 0 | 0 | 0 | 0 | 0 | 83 | 0 | 7 | 1 | 0 |
| 1ddt | A | APU | 0.963 | 0.571 | 0.4 | 0.987 | 0.46 | 0.471 | 8 | 461 | 6 | 12 | 1 | 2 |
| 1e3v | A | DXC | 0.95 | 0.769 | 0.769 | 0.972 | 0.741 | 0.769 | 10 | 103 | 3 | 3 | 1 | 1 |
| 1e4g | T | ATP | 0.948 | 0.538 | 0.7 | 0.963 | 0.587 | 0.609 | 14 | 313 | 12 | 6 | 1 | 1 |
| 1e5q | A | SHR | 0.934 | 0.273 | 0.75 | 0.94 | 0.427 | 0.4 | 9 | 375 | 24 | 3 | 2 | 3 |
| 1e5q | A | NDP | 0.961 | 0.697 | 0.793 | 0.974 | 0.723 | 0.742 | 23 | 372 | 10 | 6 | 2 | 3 |
| 1efy | A | BZC | 0.962 | 0.5 | 0.75 | 0.971 | 0.594 | 0.6 | 9 | 298 | 9 | 3 | 1 | 2 |
| 1ein | A | PLC | 0.938 | 0.5 | 0.2 | 0.987 | 0.29 | 0.286 | 3 | 224 | 3 | 12 | 1 | 1 |
| 1epb | B | REA | 0.94 | 0.762 | 0.8 | 0.962 | 0.746 | 0.78 | 16 | 126 | 5 | 4 | 1 | 1 |
| 1esw | A | ACR | 0.933 | 0.421 | 0.296 | 0.974 | 0.319 | 0.348 | 8 | 411 | 11 | 19 | 1 | 3 |
| 1eve | A | E20 | 0.971 | 0.591 | 0.765 | 0.979 | 0.658 | 0.667 | 13 | 427 | 9 | 4 | 1 | 2 |
| 1ex8 | A | A4P | 0.889 | 0.786 | 0.458 | 0.975 | 0.545 | 0.579 | 11 | 117 | 3 | 13 | 1 | 1 |
| 1exa | A | 394 | 0.986 | 1 | 0.857 | 1 | 0.919 | 0.923 | 18 | 197 | 0 | 3 | 1 | 1 |
| 1eyr | A | CDP | 0.916 | 0.5 | 0.059 | 0.995 | 0.15 | 0.105 | 1 | 184 | 1 | 16 | 1 | 1 |
| 1ezq | A | RPR | 0.935 | 0.636 | 0.7 | 0.959 | 0.631 | 0.667 | 14 | 186 | 8 | 6 | 1 | 2 |
| 1f0r | A | 815 | 0.949 | 0.6 | 0.8 | 0.96 | 0.667 | 0.686 | 12 | 192 | 8 | 3 | 2 | 2 |
| 1f0t | A | PR1 | 0.962 | 0.765 | 0.765 | 0.979 | 0.744 | 0.765 | 13 | 190 | 4 | 4 | 2 | 1 |
| 1f17 | A | NAI | 0.974 | 0.882 | 0.75 | 0.992 | 0.8 | 0.811 | 15 | 243 | 2 | 5 | 1 | 2 |
| 1f4e | A | TPR | 0.915 | 0.375 | 0.6 | 0.935 | 0.432 | 0.462 | 9 | 217 | 15 | 6 | 1 | 3 |
| 1f4f | A | TP3 | 0.93 | 0.368 | 0.583 | 0.948 | 0.429 | 0.452 | 7 | 219 | 12 | 5 | 1 | 4 |
| 1f4g | A | TP4 | 0.934 | 0.571 | 0.632 | 0.96 | 0.565 | 0.6 | 12 | 215 | 9 | 7 | 1 | 2 |
| 1f7b | A | NAU | 0.966 | 0.667 | 0.875 | 0.971 | 0.746 | 0.757 | 14 | 238 | 7 | 2 | 1 | 1 |
| 1f8g | A | NAD | 0.95 | 0.591 | 0.619 | 0.972 | 0.578 | 0.605 | 13 | 309 | 9 | 8 | 1 | 2 |
| 1fcv | A | GCU | 0.973 | 0.533 | 0.889 | 0.976 | 0.677 | 0.667 | 8 | 279 | 7 | 1 | 1 | 1 |
| 1fjs | A | Z34 | 0.963 | 0.778 | 0.778 | 0.98 | 0.757 | 0.778 | 14 | 193 | 4 | 4 | 1 | 1 |
| 1fk6 | A | LNL | 0 | 0 | 0 | 0 | 0 | 0 | 0 | 79 | 0 | 13 | 1 | 0 |
| 1fkg | A | SB3 | 0.958 | 1 | 0.6 | 1 | 0.757 | 0.75 | 6 | 85 | 0 | 4 | 1 | 1 |
| 1foa | A | UD1 | 0.925 | 0.471 | 0.762 | 0.937 | 0.562 | 0.582 | 16 | 267 | 18 | 5 | 1 | 2 |
| 1fth | A | A3P | 0.938 | 0 | 0 | 0.972 | -0.032 | 0 | 0 | 105 | 3 | 4 | 1 | 1 |
| 1g4o | A | BSB | 0.958 | 0.579 | 0.846 | 0.964 | 0.68 | 0.688 | 11 | 217 | 8 | 2 | 1 | 1 |
| 1g6c | A | POP | 0.898 | 0.238 | 0.556 | 0.915 | 0.318 | 0.333 | 5 | 172 | 16 | 4 | 3 | 2 |
| 1g6c | A | TZP | 0.934 | 0.429 | 0.9 | 0.936 | 0.595 | 0.581 | 9 | 175 | 12 | 1 | 3 | 2 |
| 1g6c | A | IFP | 0.944 | 0.524 | 0.917 | 0.946 | 0.669 | 0.667 | 11 | 175 | 10 | 1 | 3 | 2 |
| 1g97 | A | UD1 | 0.971 | 0.786 | 0.786 | 0.984 | 0.77 | 0.786 | 22 | 374 | 6 | 6 | 1 | 2 |
| 1goq | A | XYP | 0.958 | 0.923 | 0.545 | 0.996 | 0.692 | 0.686 | 12 | 242 | 1 | 10 | 1 | 1 |
| 1goy | A | 3GP | 0.909 | 1 | 0.25 | 1 | 0.476 | 0.4 | 3 | 87 | 0 | 9 | 1 | 1 |
| 1gpk | A | HUP | 0.972 | 0.542 | 0.929 | 0.974 | 0.698 | 0.684 | 13 | 409 | 11 | 1 | 1 | 2 |
| 1gsa | A | ADP | 0.959 | 0.679 | 0.864 | 0.967 | 0.744 | 0.76 | 19 | 261 | 9 | 3 | 1 | 2 |
| 1gwx | A | 433 | 0.927 | 0.647 | 0.458 | 0.975 | 0.507 | 0.537 | 11 | 230 | 6 | 13 | 1 | 1 |
| 1h0s | A | FA6 | 0.954 | 1 | 0.538 | 1 | 0.716 | 0.7 | 7 | 118 | 0 | 6 | 1 | 1 |
| 1h1p | A | CMG | 0.944 | 0.5 | 0.667 | 0.961 | 0.549 | 0.571 | 10 | 244 | 10 | 5 | 1 | 2 |
| 1h1s | A | 4SP | 0.956 | 0.619 | 0.765 | 0.969 | 0.665 | 0.684 | 13 | 247 | 8 | 4 | 1 | 2 |
| 1h72 | C | HSE | 0.906 | 0.286 | 0.667 | 0.917 | 0.396 | 0.4 | 8 | 222 | 20 | 4 | 2 | 1 |
| 1h72 | C | ANP | 0.957 | 0.679 | 0.905 | 0.961 | 0.762 | 0.776 | 19 | 224 | 9 | 2 | 2 | 1 |
| 1h9u | A | LG2 | 0.944 | 0.909 | 0.526 | 0.994 | 0.667 | 0.667 | 10 | 158 | 1 | 9 | 1 | 1 |
| 1hdq | A | INF | 0.928 | 0.385 | 0.714 | 0.94 | 0.491 | 0.5 | 10 | 249 | 16 | 4 | 1 | 2 |
| 1hfc | A | PLH | 0.925 | 0.636 | 0.5 | 0.97 | 0.524 | 0.56 | 7 | 128 | 4 | 7 | 1 | 2 |
| 1hnj | A | MLC | 0.887 | 0 | 0 | 0.894 | -0.029 | 0 | 0 | 244 | 29 | 2 | 2 | 2 |
| 1hnj | A | PO4 | 0.92 | 0.483 | 0.667 | 0.941 | 0.525 | 0.56 | 14 | 239 | 15 | 7 | 2 | 2 |
| 1hp0 | A | AD3 | 0.992 | 0.938 | 0.938 | 0.996 | 0.933 | 0.938 | 15 | 248 | 1 | 1 | 1 | 1 |
| 1hpv | A | 478 | 0.892 | 1 | 0.286 | 1 | 0.504 | 0.444 | 4 | 79 | 0 | 10 | 1 | 1 |
| 1htf | A | G26 | 0 | 0 | 0 | 0 | 0 | 0 | 0 | 79 | 0 | 15 | 1 | 0 |
| 1hww | A | SWA | 0.965 | 0.25 | 1 | 0.964 | 0.491 | 0.4 | 11 | 889 | 33 | 0 | 1 | 6 |
| 1i1h | A | COJ | 0.914 | 0.429 | 0.2 | 0.977 | 0.253 | 0.273 | 3 | 167 | 4 | 12 | 1 | 2 |
| 1i7l | A | ATP | 0.931 | 0.469 | 0.833 | 0.938 | 0.594 | 0.6 | 15 | 255 | 17 | 3 | 1 | 1 |
| 1i7z | A | COC | 0 | 0 | 0 | 0 | 0 | 0 | 0 | 203 | 0 | 9 | 1 | 0 |
| 1i8z | A | INL | 0.946 | 0.619 | 0.765 | 0.961 | 0.659 | 0.684 | 13 | 196 | 8 | 4 | 1 | 2 |
| 1if7 | A | SBR | 0.945 | 0.522 | 0.857 | 0.95 | 0.643 | 0.649 | 12 | 210 | 11 | 2 | 1 | 2 |
| 1ig3 | A | VIB | 0 | 0 | 0 | 0 | 0 | 0 | 0 | 226 | 0 | 11 | 1 | 0 |
| 1ixn | A | DXP | 0.942 | 0.526 | 0.769 | 0.954 | 0.607 | 0.625 | 10 | 185 | 9 | 3 | 2 | 1 |
| 1ixn | A | G3P | 0.952 | 0.526 | 0.909 | 0.954 | 0.671 | 0.667 | 10 | 187 | 9 | 1 | 2 | 1 |
| 1j9k | A | WO4 | 0.987 | 0.75 | 0.857 | 0.991 | 0.795 | 0.8 | 6 | 221 | 2 | 1 | 1 | 1 |
| 1jcg | A | ANP | 0.974 | 0.81 | 0.81 | 0.986 | 0.795 | 0.81 | 17 | 278 | 4 | 4 | 1 | 1 |
| 1jcl | B | HPD | 0.986 | 0.812 | 1 | 0.985 | 0.894 | 0.897 | 13 | 193 | 3 | 0 | 1 | 1 |
| 1jd0 | A | AZM | 0.987 | 0.769 | 1 | 0.987 | 0.871 | 0.87 | 10 | 221 | 3 | 0 | 1 | 1 |
| 1jie | A | BLM | 0 | 0 | 0 | 0 | 0 | 0 | 0 | 96 | 0 | 21 | 1 | 0 |
| 1jje | A | BYS | 0.975 | 0.846 | 0.786 | 0.989 | 0.802 | 0.815 | 11 | 187 | 2 | 3 | 1 | 1 |
| 1jkk | A | ANP | 0.946 | 0.591 | 0.722 | 0.963 | 0.625 | 0.65 | 13 | 233 | 9 | 5 | 1 | 2 |
| 1jla | A | TNK | 0.952 | 0.345 | 0.625 | 0.963 | 0.442 | 0.444 | 10 | 490 | 19 | 6 | 1 | 4 |
| 1jq3 | A | AAT | 0.966 | 0.889 | 0.8 | 0.987 | 0.824 | 0.842 | 24 | 229 | 3 | 6 | 1 | 1 |
| 1jsv | A | U55 | 0.955 | 0.5 | 0.833 | 0.961 | 0.625 | 0.625 | 10 | 244 | 10 | 2 | 1 | 1 |
| 1jxm | A | 5GP | 0.954 | 0 | 0 | 0.986 | -0.021 | 0 | 0 | 209 | 3 | 7 | 2 | 1 |
| 1jxm | A | GAI | 0.973 | 0 | 0 | 0.986 | -0.014 | 0 | 0 | 213 | 3 | 3 | 2 | 1 |
| 1jyl | A | CDC | 0.933 | 0.684 | 0.619 | 0.968 | 0.614 | 0.65 | 13 | 183 | 6 | 8 | 1 | 1 |
| 1k1j | A | FD2 | 0.976 | 0.789 | 0.938 | 0.979 | 0.848 | 0.857 | 15 | 187 | 4 | 1 | 1 | 1 |
| 1k22 | H | MEL | 0.961 | 0.727 | 0.842 | 0.971 | 0.761 | 0.78 | 16 | 204 | 6 | 3 | 1 | 1 |
| 1k3u | A | IAD | 0.955 | 0.643 | 0.947 | 0.956 | 0.759 | 0.766 | 18 | 216 | 10 | 1 | 1 | 1 |
| 1k3y | A | GTX | 0.923 | 0.4 | 0.286 | 0.969 | 0.298 | 0.333 | 4 | 188 | 6 | 10 | 1 | 1 |
| 1k54 | A | HOQ | 0 | 0 | 0 | 0 | 0 | 0 | 0 | 206 | 0 | 9 | 1 | 0 |
| 1k7e | A | IAG | 0.98 | 0.773 | 1 | 0.978 | 0.869 | 0.872 | 17 | 224 | 5 | 0 | 1 | 1 |
| 1k7f | A | IAV | 0.946 | 0.5 | 0.615 | 0.965 | 0.526 | 0.552 | 8 | 218 | 8 | 5 | 1 | 1 |
| 1kaq | A | DND | 0.942 | 0.815 | 0.815 | 0.966 | 0.781 | 0.815 | 22 | 141 | 5 | 5 | 1 | 3 |
| 1ke5 | A | LS1 | 0.954 | 0.556 | 0.714 | 0.968 | 0.606 | 0.625 | 10 | 239 | 8 | 4 | 1 | 2 |
| 1kgz | B | PRP | 0.954 | 0.542 | 0.812 | 0.962 | 0.641 | 0.65 | 13 | 277 | 11 | 3 | 1 | 2 |
| 1khr | D | COA | 0 | 0 | 0 | 0 | 0 | 0 | 0 | 180 | 0 | 18 | 2 | 0 |
| 1khr | D | VIR | 0 | 0 | 0 | 0 | 0 | 0 | 0 | 195 | 0 | 3 | 2 | 0 |
| 1kpe | A | ADW | 0.901 | 0 | 0 | 0.91 | -0.031 | 0 | 0 | 91 | 9 | 1 | 1 | 1 |
| 1kv1 | A | BMU | 0.926 | 0.37 | 0.625 | 0.942 | 0.445 | 0.465 | 10 | 278 | 17 | 6 | 1 | 3 |
| 1kv2 | A | B96 | 0.95 | 1 | 0.348 | 1 | 0.575 | 0.516 | 8 | 279 | 0 | 15 | 1 | 1 |
| 1kwc | B | BPY | 0.97 | 0.636 | 1 | 0.969 | 0.785 | 0.778 | 14 | 246 | 8 | 0 | 1 | 1 |
| 1l2s | A | STC | 0.958 | 0.571 | 0.533 | 0.979 | 0.53 | 0.552 | 8 | 285 | 6 | 7 | 1 | 1 |
| 1l7f | A | BCZ | 0.945 | 0 | 0 | 0.994 | -0.017 | 0 | 0 | 344 | 2 | 18 | 1 | 1 |
| 1l8g | A | DBD | 0.989 | 0.929 | 0.867 | 0.996 | 0.891 | 0.897 | 13 | 246 | 1 | 2 | 1 | 1 |
| 1lby | A | F6P | 0.948 | 0.533 | 0.667 | 0.965 | 0.569 | 0.593 | 8 | 192 | 7 | 4 | 2 | 1 |
| 1lby | A | PO4 | 0.962 | 0.467 | 1 | 0.961 | 0.67 | 0.636 | 7 | 196 | 8 | 0 | 2 | 1 |
| 1llo | A | AMI | 0.958 | 0.579 | 0.846 | 0.964 | 0.68 | 0.688 | 11 | 216 | 8 | 2 | 1 | 2 |
| 1lpz | B | CMB | 0.958 | 0.778 | 0.737 | 0.98 | 0.734 | 0.757 | 14 | 193 | 4 | 5 | 1 | 1 |
| 1lqd | B | CMI | 0.972 | 0.889 | 0.8 | 0.99 | 0.828 | 0.842 | 16 | 192 | 2 | 4 | 1 | 1 |
| 1lsp | A | BUL | 0.849 | 0 | 0 | 0.97 | -0.062 | 0 | 0 | 129 | 4 | 19 | 1 | 1 |
| 1ltz | A | HBI | 0.944 | 0.417 | 0.417 | 0.971 | 0.388 | 0.417 | 5 | 233 | 7 | 7 | 1 | 1 |
| 1lzs | A | NAG | 0 | 0 | 0 | 0 | 0 | 0 | 0 | 96 | 0 | 23 | 1 | 0 |
| 1m48 | A | FRG | 0 | 0 | 0 | 0 | 0 | 0 | 0 | 99 | 0 | 12 | 1 | 0 |
| 1mbz | A | IOT | 0.962 | 0.357 | 1 | 0.961 | 0.586 | 0.526 | 10 | 440 | 18 | 0 | 2 | 2 |
| 1mbz | A | POP | 0.97 | 0.714 | 0.769 | 0.982 | 0.725 | 0.741 | 20 | 434 | 8 | 6 | 2 | 2 |
| 1meh | A | IMP | 0.967 | 0.7 | 0.778 | 0.979 | 0.72 | 0.737 | 14 | 278 | 6 | 4 | 1 | 2 |
| 1mka | A | DAC | 0.889 | 0.75 | 0.15 | 0.993 | 0.303 | 0.25 | 3 | 141 | 1 | 17 | 1 | 1 |
| 1mmb | A | BAT | 0.951 | 0.833 | 0.667 | 0.984 | 0.72 | 0.741 | 10 | 127 | 2 | 5 | 1 | 1 |
| 1mnc | A | PLH | 0.929 | 0.667 | 0.571 | 0.968 | 0.578 | 0.615 | 8 | 122 | 4 | 6 | 1 | 1 |
| 1mq5 | A | XLC | 0.948 | 0.737 | 0.7 | 0.974 | 0.69 | 0.718 | 14 | 186 | 5 | 6 | 1 | 1 |
| 1mq6 | A | XLD | 0.958 | 0.789 | 0.75 | 0.979 | 0.746 | 0.769 | 15 | 189 | 4 | 5 | 1 | 1 |
| 1mu0 | A | PHK | 0.939 | 0.556 | 0.294 | 0.984 | 0.376 | 0.385 | 5 | 243 | 4 | 12 | 1 | 1 |
| 1mxi | A | SAH | 0.903 | 1 | 0.176 | 1 | 0.399 | 0.3 | 3 | 128 | 0 | 14 | 1 | 1 |
| 1n07 | A | ADP | 0.864 | 0.455 | 0.263 | 0.953 | 0.276 | 0.333 | 5 | 122 | 6 | 14 | 2 | 1 |
| 1n07 | A | FMN | 0.891 | 0.455 | 0.333 | 0.955 | 0.331 | 0.385 | 5 | 126 | 6 | 10 | 2 | 1 |
| 1n2v | A | BDI | 0.942 | 0.387 | 1 | 0.939 | 0.603 | 0.558 | 12 | 294 | 19 | 0 | 1 | 2 |
| 1n46 | A | PFA | 0 | 0 | 0 | 0 | 0 | 0 | 0 | 210 | 0 | 22 | 1 | 0 |
| 1nav | A | IH5 | 0.961 | 0.875 | 0.667 | 0.99 | 0.744 | 0.757 | 14 | 208 | 2 | 7 | 1 | 1 |
| 1nco | A | CHR | 0.972 | 0 | 0 | 0.981 | -0.013 | 0 | 0 | 104 | 2 | 1 | 1 | 1 |
| 1ndi | A | COA | 0.952 | 0.409 | 0.391 | 0.976 | 0.375 | 0.4 | 9 | 531 | 13 | 14 | 1 | 2 |
| 1nhu | A | 153 | 0.933 | 0.133 | 0.333 | 0.948 | 0.181 | 0.19 | 4 | 473 | 26 | 8 | 1 | 4 |
| 1nhv | A | 154 | 0.939 | 0.143 | 0.364 | 0.952 | 0.201 | 0.205 | 4 | 472 | 24 | 7 | 1 | 3 |
| 1nmk | A | SFM | 0.923 | 1 | 0.2 | 1 | 0.429 | 0.333 | 3 | 141 | 0 | 12 | 1 | 1 |
| 1nz7 | A | 901 | 0.968 | 1 | 0.529 | 1 | 0.716 | 0.692 | 9 | 235 | 0 | 8 | 1 | 1 |
| 1o26 | B | FAD | 0.899 | 0.333 | 0.053 | 0.989 | 0.1 | 0.091 | 1 | 178 | 2 | 18 | 2 | 1 |
| 1o26 | B | UMP | 0.965 | 1 | 0.3 | 1 | 0.538 | 0.462 | 3 | 189 | 0 | 7 | 2 | 1 |
| 1o86 | A | GLY | 0.937 | 0.158 | 1 | 0.937 | 0.385 | 0.273 | 6 | 472 | 32 | 0 | 1 | 4 |
| 1of1 | A | SCT | 0.948 | 0.455 | 0.357 | 0.978 | 0.376 | 0.4 | 5 | 266 | 6 | 9 | 1 | 1 |
| 1of8 | B | PEP | 0.926 | 0.13 | 0.6 | 0.932 | 0.256 | 0.214 | 3 | 273 | 20 | 2 | 2 | 3 |
| 1of8 | B | G3P | 0.96 | 0.478 | 1 | 0.958 | 0.677 | 0.647 | 11 | 275 | 12 | 0 | 2 | 3 |
| 1og1 | A | TAD | 0.932 | 0.727 | 0.421 | 0.984 | 0.521 | 0.533 | 8 | 184 | 3 | 11 | 1 | 1 |
| 1ogk | B | DUD | 0.963 | 0.765 | 0.765 | 0.98 | 0.744 | 0.765 | 13 | 193 | 4 | 4 | 1 | 2 |
| 1ohr | A | 1UN | 0.855 | 0.667 | 0.154 | 0.986 | 0.272 | 0.25 | 2 | 69 | 1 | 11 | 1 | 1 |
| 1ojz | A | NAD | 0 | 0 | 0 | 0 | 0 | 0 | 0 | 180 | 0 | 18 | 1 | 0 |
| 1onh | A | WY4 | 0.928 | 0.24 | 0.6 | 0.938 | 0.349 | 0.343 | 6 | 289 | 19 | 4 | 1 | 1 |
| 1oq5 | A | CEL | 0.96 | 0.684 | 0.812 | 0.971 | 0.724 | 0.743 | 13 | 202 | 6 | 3 | 1 | 1 |
| 1ow3 | B | GDP | 0.913 | 0.5 | 0.643 | 0.939 | 0.52 | 0.563 | 9 | 138 | 9 | 5 | 2 | 1 |
| 1ow3 | B | MGF | 0.938 | 0.5 | 0.9 | 0.94 | 0.644 | 0.643 | 9 | 142 | 9 | 1 | 2 | 1 |
| 1owe | A | 675 | 0.978 | 0.765 | 0.929 | 0.981 | 0.831 | 0.839 | 13 | 205 | 4 | 1 | 1 | 1 |
| 1oxm | A | TC4 | 0.922 | 0.714 | 0.312 | 0.987 | 0.439 | 0.435 | 5 | 148 | 2 | 11 | 1 | 2 |
| 1oxv | A | ANP | 0.99 | 1 | 0.75 | 1 | 0.862 | 0.857 | 9 | 292 | 0 | 3 | 1 | 1 |
| 1oyt | H | FSN | 0.934 | 0.565 | 0.722 | 0.952 | 0.604 | 0.634 | 13 | 200 | 10 | 5 | 1 | 2 |
| 1p2y | A | HEM | 0.931 | 0.514 | 0.704 | 0.948 | 0.565 | 0.594 | 19 | 330 | 18 | 8 | 1 | 2 |
| 1p5r | A | COA | 0.94 | 0.412 | 0.333 | 0.974 | 0.339 | 0.368 | 7 | 370 | 10 | 14 | 1 | 4 |
| 1p77 | A | ATR | 0.898 | 0.31 | 0.692 | 0.91 | 0.419 | 0.429 | 9 | 202 | 20 | 4 | 1 | 3 |
| 1pfk | A | ADP | 0.882 | 0.455 | 0.333 | 0.95 | 0.326 | 0.385 | 10 | 229 | 12 | 20 | 1 | 1 |
| 1pfk | A | FBP | 0.945 | 0.409 | 0.818 | 0.95 | 0.555 | 0.545 | 9 | 247 | 13 | 2 | 1 | 1 |
| 1pkk | A | DCP | 0.989 | 0 | 0 | 0.989 | 0 | 0 | 0 | 173 | 2 | 0 | 1 | 1 |
| 1pmn | A | 984 | 0.969 | 0.812 | 0.65 | 0.99 | 0.711 | 0.722 | 13 | 303 | 3 | 7 | 1 | 2 |
| 1pw1 | A | HEL | 0.97 | 0.857 | 0.632 | 0.993 | 0.721 | 0.727 | 12 | 280 | 2 | 7 | 1 | 1 |
| 1q41 | A | IXM | 0.962 | 0.556 | 0.714 | 0.973 | 0.611 | 0.625 | 10 | 292 | 8 | 4 | 1 | 1 |
| 1q51 | A | CAA | 0.927 | 0.5 | 0.278 | 0.978 | 0.337 | 0.357 | 5 | 222 | 5 | 13 | 1 | 1 |
| 1q8j | A | HCS | 0.919 | 0.125 | 0.267 | 0.94 | 0.144 | 0.17 | 4 | 437 | 28 | 11 | 2 | 2 |
| 1q8j | A | C2F | 0.96 | 0.406 | 1 | 0.959 | 0.624 | 0.578 | 13 | 448 | 19 | 0 | 2 | 2 |
| 1qbu | A | 846 | 0.891 | 1 | 0.286 | 1 | 0.503 | 0.444 | 4 | 78 | 0 | 10 | 1 | 1 |
| 1qhi | A | BPG | 0.957 | 0.579 | 0.733 | 0.97 | 0.629 | 0.647 | 11 | 255 | 8 | 4 | 1 | 1 |
| 1qji | A | PKF | 0.957 | 0.8 | 0.706 | 0.982 | 0.729 | 0.75 | 12 | 168 | 3 | 5 | 1 | 2 |
| 1ql9 | A | ZEN | 0.966 | 0.824 | 0.778 | 0.984 | 0.782 | 0.8 | 14 | 186 | 3 | 4 | 1 | 1 |
| 1qpe | A | PP2 | 0.976 | 0.706 | 0.923 | 0.979 | 0.796 | 0.8 | 12 | 233 | 5 | 1 | 1 | 1 |
| 1qti | A | GNT | 0.989 | 0.8 | 0.941 | 0.991 | 0.862 | 0.865 | 16 | 430 | 4 | 1 | 1 | 1 |
| 1r09 | 1 | JEN | 0.937 | 0.36 | 0.9 | 0.938 | 0.546 | 0.514 | 9 | 242 | 16 | 1 | 1 | 1 |
| 1r15 | A | NCA | 0.94 | 0.25 | 0.2 | 0.973 | 0.193 | 0.222 | 2 | 216 | 6 | 8 | 2 | 1 |
| 1r15 | A | N | 0.974 | 0.875 | 0.583 | 0.995 | 0.703 | 0.7 | 7 | 219 | 1 | 5 | 2 | 1 |
| 1r1h | A | BIR | 0.948 | 0.333 | 0.941 | 0.949 | 0.543 | 0.492 | 16 | 590 | 32 | 1 | 1 | 5 |
| 1r55 | A | 97 | 0.91 | 0.462 | 0.4 | 0.957 | 0.381 | 0.429 | 6 | 155 | 7 | 9 | 1 | 2 |
| 1r58 | A | AO5 | 0.958 | 0.519 | 0.933 | 0.959 | 0.678 | 0.667 | 14 | 302 | 13 | 1 | 1 | 2 |
| 1r9o | A | HEM | 0.908 | 0.368 | 0.483 | 0.939 | 0.373 | 0.418 | 14 | 371 | 24 | 15 | 1 | 2 |
| 1ri1 | A | GTG | 0.889 | 0.143 | 0.3 | 0.917 | 0.153 | 0.194 | 3 | 198 | 18 | 7 | 2 | 1 |
| 1ri1 | A | SAH | 0.96 | 0.667 | 0.875 | 0.967 | 0.744 | 0.757 | 14 | 203 | 7 | 2 | 2 | 1 |
| 1rn8 | A | DUP | 0.941 | 0.571 | 0.444 | 0.976 | 0.473 | 0.5 | 4 | 123 | 3 | 5 | 1 | 2 |
| 1rsd | A | PSB | 0.938 | 1 | 0.222 | 1 | 0.456 | 0.364 | 2 | 103 | 0 | 7 | 1 | 1 |
| 1rya | A | GDP | 0.894 | 0.389 | 0.583 | 0.921 | 0.421 | 0.467 | 7 | 128 | 11 | 5 | 1 | 1 |
| 1rzu | A | ADP | 0.929 | 0.214 | 0.429 | 0.946 | 0.269 | 0.286 | 6 | 384 | 22 | 8 | 1 | 2 |
| 1s19 | A | MC9 | 0.971 | 0.9 | 0.783 | 0.991 | 0.824 | 0.837 | 18 | 213 | 2 | 5 | 1 | 2 |
| 1s3f | A | SNI | 0.933 | 1 | 0.25 | 1 | 0.483 | 0.4 | 3 | 122 | 0 | 9 | 1 | 1 |
| 1s3v | A | TQD | 0.835 | 0.2 | 0.235 | 0.899 | 0.125 | 0.216 | 4 | 143 | 16 | 13 | 1 | 2 |
| 1s7l | A | COA | 0.926 | 0.7 | 0.7 | 0.958 | 0.658 | 0.7 | 14 | 136 | 6 | 6 | 1 | 1 |
| 1sby | A | NAD | 0.904 | 0.192 | 0.833 | 0.905 | 0.372 | 0.312 | 5 | 201 | 21 | 1 | 2 | 1 |
| 1sby | A | ETF | 0.974 | 0.923 | 0.857 | 0.99 | 0.875 | 0.889 | 24 | 198 | 2 | 4 | 2 | 1 |
| 1sj0 | A | E4D | 0.915 | 0.556 | 0.25 | 0.98 | 0.335 | 0.345 | 5 | 200 | 4 | 15 | 1 | 2 |
| 1sqf | A | SAM | 0.956 | 0.529 | 0.529 | 0.977 | 0.506 | 0.529 | 9 | 338 | 8 | 8 | 1 | 2 |
| 1sqn | A | NDR | 0.913 | 0 | 0 | 0.986 | -0.032 | 0 | 0 | 211 | 3 | 17 | 1 | 1 |
| 1sth | A | THP | 0.936 | 0.7 | 0.583 | 0.973 | 0.605 | 0.636 | 7 | 110 | 3 | 5 | 1 | 1 |
| 1svw | A | GTP | 0.939 | 0.929 | 0.565 | 0.994 | 0.697 | 0.703 | 13 | 156 | 1 | 10 | 1 | 1 |
| 1swk | A | BTN | 0.938 | 1 | 0.588 | 1 | 0.74 | 0.741 | 10 | 96 | 0 | 7 | 1 | 1 |
| 1sz3 | A | GNP | 0.902 | 0.333 | 0.077 | 0.985 | 0.123 | 0.125 | 1 | 128 | 2 | 12 | 1 | 1 |
| 1szj | G | NAD | 0.92 | 0.484 | 0.652 | 0.942 | 0.52 | 0.556 | 15 | 262 | 16 | 8 | 1 | 3 |
| 1tei | A | NAG | 0.958 | 0.6 | 0.545 | 0.98 | 0.55 | 0.571 | 6 | 198 | 4 | 5 | 1 | 1 |
| 1tjw | A | AS1 | 0.981 | 1 | 0.333 | 1 | 0.572 | 0.5 | 4 | 405 | 0 | 8 | 1 | 2 |
| 1tky | A | A3S | 0.97 | 0.846 | 0.733 | 0.989 | 0.772 | 0.786 | 11 | 185 | 2 | 4 | 1 | 1 |
| 1tow | A | CRZ | 0.953 | 0.765 | 0.867 | 0.964 | 0.788 | 0.812 | 13 | 108 | 4 | 2 | 1 | 1 |
| 1tvp | A | CBI | 0.962 | 0 | 0 | 0.962 | 0 | 0 | 0 | 228 | 9 | 0 | 1 | 1 |
| 1txc | A | 2AN | 0.852 | 0.5 | 0.391 | 0.932 | 0.359 | 0.439 | 9 | 123 | 9 | 14 | 1 | 1 |
| 1u4d | A | DBQ | 0.975 | 0.733 | 0.846 | 0.982 | 0.775 | 0.786 | 11 | 224 | 4 | 2 | 1 | 1 |
| 1u72 | A | NDP | 0.881 | 0.429 | 0.5 | 0.924 | 0.396 | 0.462 | 9 | 146 | 12 | 9 | 2 | 1 |
| 1u72 | A | MTX | 0.926 | 0.81 | 0.654 | 0.973 | 0.687 | 0.723 | 17 | 146 | 4 | 9 | 2 | 1 |
| 1u7z | A | PMT | 0.9 | 0.773 | 0.531 | 0.97 | 0.588 | 0.63 | 17 | 163 | 5 | 15 | 1 | 2 |
| 1uam | A | SAH | 0.927 | 1 | 0.15 | 1 | 0.373 | 0.261 | 3 | 214 | 0 | 17 | 1 | 1 |
| 1ucd | A | URA | 0.924 | 0.267 | 0.667 | 0.934 | 0.39 | 0.381 | 4 | 155 | 11 | 2 | 2 | 2 |
| 1ucd | A | U5P | 0.942 | 0.533 | 0.727 | 0.957 | 0.593 | 0.615 | 8 | 154 | 7 | 3 | 2 | 2 |
| 1uf8 | A | ING | 0.965 | 1 | 0.4 | 1 | 0.621 | 0.571 | 6 | 245 | 0 | 9 | 1 | 1 |
| 1um0 | A | FMN | 0.942 | 0.533 | 0.381 | 0.978 | 0.421 | 0.444 | 8 | 314 | 7 | 13 | 1 | 1 |
| 1uml | A | FR4 | 0.967 | 0.667 | 0.889 | 0.972 | 0.754 | 0.762 | 16 | 279 | 8 | 2 | 1 | 3 |
| 1unl | A | RRC | 0.959 | 0.733 | 0.611 | 0.984 | 0.648 | 0.667 | 11 | 245 | 4 | 7 | 1 | 2 |
| 1uou | A | CMU | 0.93 | 0.333 | 0.867 | 0.932 | 0.512 | 0.481 | 13 | 359 | 26 | 2 | 1 | 2 |
| 1uvs | H | I11 | 0.952 | 0.7 | 0.778 | 0.968 | 0.712 | 0.737 | 14 | 183 | 6 | 4 | 1 | 2 |
| 1uvt | H | I48 | 0.945 | 0.652 | 0.789 | 0.96 | 0.688 | 0.714 | 15 | 192 | 8 | 4 | 1 | 1 |
| 1uyy | A | BGC | 0.862 | 0.417 | 0.333 | 0.935 | 0.296 | 0.37 | 5 | 101 | 7 | 10 | 1 | 2 |
| 1v0p | A | PVB | 0.969 | 0.857 | 0.667 | 0.992 | 0.74 | 0.75 | 12 | 238 | 2 | 6 | 1 | 1 |
| 1v0y | A | HI5 | 0.982 | 0.6 | 0.6 | 0.991 | 0.591 | 0.6 | 6 | 427 | 4 | 4 | 1 | 2 |
| 1v3s | A | ATP | 0.859 | 0.889 | 0.4 | 0.986 | 0.536 | 0.552 | 8 | 71 | 1 | 12 | 1 | 2 |
| 1v48 | A | HA1 | 0.961 | 0.7 | 0.824 | 0.972 | 0.739 | 0.757 | 14 | 207 | 6 | 3 | 1 | 2 |
| 1vcj | A | IBA | 0.945 | 0.467 | 0.389 | 0.976 | 0.398 | 0.424 | 7 | 321 | 8 | 11 | 1 | 3 |
| 1vcu | A | DAN | 0.988 | 0.867 | 0.867 | 0.994 | 0.86 | 0.867 | 13 | 318 | 2 | 2 | 1 | 1 |
| 1vh3 | A | CMK | 0 | 0 | 0 | 0 | 0 | 0 | 0 | 161 | 0 | 19 | 1 | 0 |
| 1vps | B | SIA | 0.909 | 0 | 0 | 0.954 | -0.047 | 0 | 0 | 251 | 12 | 13 | 1 | 4 |
| 1w1p | A | GIO | 0.963 | 0.346 | 1 | 0.962 | 0.577 | 0.514 | 9 | 433 | 17 | 0 | 1 | 1 |
| 1wnz | A | 2VA | 0.914 | 0.75 | 0.188 | 0.993 | 0.347 | 0.3 | 3 | 145 | 1 | 13 | 1 | 1 |
| 1wop | A | FFO | 0.91 | 0.52 | 0.433 | 0.959 | 0.426 | 0.473 | 13 | 280 | 12 | 17 | 1 | 1 |
| 1wq1 | G | GDP | 0 | 0 | 0 | 0 | 0 | 0 | 0 | 293 | 0 | 1 | 2 | 0 |
| 1wq1 | G | AF3 | 0 | 0 | 0 | 0 | 0 | 0 | 0 | 291 | 0 | 3 | 2 | 0 |
| 1wxi | A | DPO | 0.955 | 0.25 | 0.286 | 0.975 | 0.244 | 0.267 | 2 | 233 | 6 | 5 | 2 | 2 |
| 1wxi | A | AMP | 0.927 | 0.5 | 0.222 | 0.982 | 0.3 | 0.308 | 4 | 224 | 4 | 14 | 2 | 2 |
| 1x2b | A | STX | 0.965 | 0.6 | 1 | 0.963 | 0.76 | 0.75 | 15 | 262 | 10 | 0 | 1 | 1 |
| 1x55 | A | NSS | 0.912 | 0.079 | 1 | 0.912 | 0.268 | 0.146 | 3 | 361 | 35 | 0 | 2 | 3 |
| 1x55 | A | PO4 | 0.952 | 0.526 | 0.952 | 0.952 | 0.688 | 0.678 | 20 | 360 | 18 | 1 | 2 | 3 |
| 1x6u | A | DO8 | 0.963 | 0.667 | 0.8 | 0.974 | 0.711 | 0.727 | 12 | 224 | 6 | 3 | 1 | 1 |
| 1x7p | A | SAM | 0.939 | 1 | 0.167 | 1 | 0.395 | 0.286 | 3 | 228 | 0 | 15 | 1 | 1 |
| 1x8x | A | TYR | 0.946 | 0.44 | 0.846 | 0.951 | 0.587 | 0.579 | 11 | 271 | 14 | 2 | 1 | 2 |
| 1xm6 | A | 5RM | 0.938 | 0.448 | 0.812 | 0.945 | 0.575 | 0.578 | 13 | 273 | 16 | 3 | 1 | 1 |
| 1xny | A | 191 | 0.94 | 0.188 | 0.158 | 0.972 | 0.141 | 0.171 | 3 | 448 | 13 | 16 | 2 | 5 |
| 1xny | A | BTN | 0.95 | 0.188 | 0.214 | 0.972 | 0.175 | 0.2 | 3 | 453 | 13 | 11 | 2 | 5 |
| 1xoq | A | ROF | 0.932 | 0.458 | 0.611 | 0.953 | 0.494 | 0.524 | 11 | 262 | 13 | 7 | 1 | 1 |
| 1xoz | A | CIA | 0.906 | 0.303 | 0.667 | 0.918 | 0.408 | 0.417 | 10 | 259 | 23 | 5 | 1 | 1 |
| 1xpy | C | NLQ | 0.947 | 0.483 | 0.875 | 0.951 | 0.627 | 0.622 | 14 | 292 | 15 | 2 | 1 | 3 |
| 1xqp | A | 8HG | 0.956 | 0.917 | 0.55 | 0.995 | 0.691 | 0.687 | 11 | 207 | 1 | 9 | 1 | 1 |
| 1xtb | A | S6P | 0.965 | 0.462 | 0.75 | 0.972 | 0.572 | 0.571 | 12 | 483 | 14 | 4 | 1 | 3 |
| 1xvt | A | COA | 0.939 | 0.6 | 0.375 | 0.981 | 0.444 | 0.462 | 9 | 314 | 6 | 15 | 1 | 2 |
| 1xwq | A | XYP | 0.96 | 0.429 | 0.818 | 0.964 | 0.575 | 0.562 | 9 | 323 | 12 | 2 | 1 | 2 |
| 1xz8 | A | 5GP | 0.934 | 0.2 | 0.5 | 0.946 | 0.288 | 0.286 | 2 | 139 | 8 | 2 | 2 | 1 |
| 1xz8 | A | 3GP | 0.954 | 0.8 | 0.615 | 0.986 | 0.678 | 0.696 | 8 | 136 | 2 | 5 | 2 | 1 |
| 1y30 | A | FMN | 0 | 0 | 0 | 0 | 0 | 0 | 0 | 128 | 0 | 9 | 1 | 0 |
| 1y3i | A | NAD | 0 | 0 | 0 | 0 | 0 | 0 | 0 | 206 | 0 | 15 | 1 | 0 |
| 1y6b | A | AAX | 0.947 | 0.875 | 0.368 | 0.996 | 0.548 | 0.519 | 7 | 224 | 1 | 12 | 1 | 1 |
| 1y8e | A | SVR | 0.963 | 0 | 0 | 0.992 | -0.016 | 0 | 0 | 234 | 2 | 7 | 1 | 1 |
| 1ybu | A | APC | 0.974 | 0.833 | 0.625 | 0.993 | 0.709 | 0.714 | 5 | 145 | 1 | 3 | 1 | 1 |
| 1ydr | E | IQP | 0.962 | 0.52 | 1 | 0.96 | 0.707 | 0.684 | 13 | 290 | 12 | 0 | 1 | 3 |
| 1yds | E | IQS | 0.974 | 0.619 | 1 | 0.973 | 0.776 | 0.765 | 13 | 285 | 8 | 0 | 1 | 2 |
| 1ydt | E | IQB | 0.962 | 0.7 | 0.7 | 0.979 | 0.679 | 0.7 | 14 | 286 | 6 | 6 | 1 | 1 |
| 1yfr | A | ATP | 0.925 | 0.179 | 0.385 | 0.943 | 0.227 | 0.244 | 5 | 378 | 23 | 8 | 1 | 3 |
| 1ygc | H | 905 | 0.944 | 0.625 | 0.789 | 0.958 | 0.673 | 0.698 | 15 | 206 | 9 | 4 | 1 | 3 |
| 1yqy | A | 915 | 0.948 | 0.406 | 0.722 | 0.957 | 0.518 | 0.52 | 13 | 421 | 19 | 5 | 1 | 4 |
| 1ytm | A | ATP | 0.95 | 0.419 | 0.722 | 0.959 | 0.527 | 0.531 | 13 | 426 | 18 | 5 | 2 | 1 |
| 1ytm | A | OXD | 0.955 | 0.323 | 1 | 0.954 | 0.555 | 0.488 | 10 | 431 | 21 | 0 | 2 | 1 |
| 1yvf | A | PO4 | 0.962 | 0 | 0 | 0.969 | -0.016 | 0 | 0 | 506 | 16 | 4 | 1 | 2 |
| 1ywr | A | LI9 | 0.934 | 0.333 | 0.167 | 0.98 | 0.204 | 0.222 | 3 | 292 | 6 | 15 | 1 | 3 |
| 1z95 | A | 198 | 0.889 | 0 | 0 | 0.99 | -0.032 | 0 | 0 | 200 | 2 | 23 | 1 | 1 |
| 1zaj | A | M2P | 0.982 | 0.739 | 1 | 0.981 | 0.851 | 0.85 | 17 | 306 | 6 | 0 | 1 | 2 |
| 1zdf | A | UPG | 0.948 | 0.667 | 0.737 | 0.967 | 0.672 | 0.7 | 14 | 203 | 7 | 5 | 1 | 1 |
| 1znz | A | GDP | 0.93 | 0.429 | 0.6 | 0.95 | 0.471 | 0.5 | 6 | 153 | 8 | 4 | 1 | 2 |
| 1zt9 | A | TRP | 0.861 | 0 | 0 | 0.978 | -0.052 | 0 | 0 | 87 | 2 | 12 | 1 | 1 |
| 1zu0 | A | CBS | 0.931 | 0.333 | 0.778 | 0.938 | 0.481 | 0.467 | 14 | 421 | 28 | 4 | 1 | 1 |
| 2ahw | A | COA | 0.934 | 0.405 | 0.652 | 0.949 | 0.482 | 0.5 | 15 | 408 | 22 | 8 | 1 | 5 |
| 2aib | A | ERG | 0.812 | 1 | 0.053 | 1 | 0.207 | 0.1 | 1 | 77 | 0 | 18 | 1 | 1 |
| 2art | A | LPA | 0.903 | 0.464 | 0.65 | 0.927 | 0.498 | 0.542 | 13 | 191 | 15 | 7 | 2 | 2 |
| 2art | A | AMP | 0.934 | 0.536 | 0.882 | 0.938 | 0.657 | 0.667 | 15 | 196 | 13 | 2 | 2 | 2 |
| 2ate | A | NIA | 0.922 | 1 | 0.312 | 1 | 0.536 | 0.476 | 5 | 125 | 0 | 11 | 1 | 1 |
| 2b3d | A | FAD | 0.897 | 1 | 0.136 | 1 | 0.349 | 0.24 | 3 | 163 | 0 | 19 | 1 | 1 |
| 2b99 | A | RDL | 0.868 | 1 | 0.227 | 1 | 0.443 | 0.37 | 5 | 107 | 0 | 17 | 1 | 1 |
| 2baw | A | VCA | 0.936 | 0.538 | 0.438 | 0.972 | 0.452 | 0.483 | 7 | 211 | 6 | 9 | 1 | 2 |
| 2bbw | A | GP5 | 0.919 | 0.857 | 0.462 | 0.988 | 0.593 | 0.6 | 12 | 169 | 2 | 14 | 1 | 1 |
| 2br1 | A | PFP | 0.934 | 0.429 | 0.643 | 0.951 | 0.492 | 0.514 | 9 | 233 | 12 | 5 | 1 | 2 |
| 2bsm | A | BSM | 0.932 | 0.611 | 0.647 | 0.96 | 0.591 | 0.629 | 11 | 167 | 7 | 6 | 1 | 1 |
| 2c6z | A | CIR | 0.972 | 1 | 0.462 | 1 | 0.67 | 0.632 | 6 | 237 | 0 | 7 | 1 | 1 |
| 2c96 | A | ATP | 0.971 | 0.833 | 0.714 | 0.99 | 0.756 | 0.769 | 10 | 192 | 2 | 4 | 1 | 1 |
| 2cht | F | TSA | 0 | 0 | 0 | 0 | 0 | 0 | 0 | 97 | 0 | 11 | 1 | 0 |
| 2chz | A | 93 | 0.977 | 0.467 | 0.875 | 0.98 | 0.63 | 0.609 | 14 | 767 | 16 | 2 | 1 | 4 |
| 2csn | A | CKI | 0.949 | 0.5 | 0.5 | 0.973 | 0.473 | 0.5 | 7 | 256 | 7 | 7 | 1 | 3 |
| 2cwh | B | NDP | 0.913 | 0.5 | 0.296 | 0.972 | 0.342 | 0.372 | 8 | 276 | 8 | 19 | 2 | 1 |
| 2cwh | B | PYC | 0.981 | 0.625 | 1 | 0.98 | 0.783 | 0.769 | 10 | 295 | 6 | 0 | 2 | 1 |
| 2cx8 | A | SAH | 0.943 | 1 | 0.368 | 1 | 0.589 | 0.538 | 7 | 191 | 0 | 12 | 1 | 1 |
| 2d29 | A | FAD | 0.94 | 0.4 | 0.471 | 0.964 | 0.402 | 0.432 | 8 | 320 | 12 | 9 | 1 | 3 |
| 2dkc | A | PO4 | 0.945 | 0.194 | 0.857 | 0.947 | 0.392 | 0.316 | 6 | 444 | 25 | 1 | 2 | 1 |
| 2dkc | A | 16G | 0.956 | 0.323 | 1 | 0.955 | 0.555 | 0.488 | 10 | 445 | 21 | 0 | 2 | 1 |
| 2dpt | A | PUY | 0.948 | 0.818 | 0.5 | 0.99 | 0.615 | 0.621 | 9 | 192 | 2 | 9 | 1 | 1 |
| 2dtt | B | H4B | 0 | 0 | 0 | 0 | 0 | 0 | 0 | 90 | 0 | 12 | 1 | 0 |
| 2dve | A | BT5 | 0.953 | 0.9 | 0.692 | 0.989 | 0.765 | 0.783 | 18 | 187 | 2 | 8 | 1 | 1 |
| 2dya | B | ADP | 0.945 | 0.7 | 0.583 | 0.977 | 0.61 | 0.636 | 7 | 130 | 3 | 5 | 1 | 1 |
| 2dzb | A | HH2 | 0.95 | 0.588 | 0.714 | 0.966 | 0.622 | 0.645 | 10 | 198 | 7 | 4 | 1 | 1 |
| 2e0n | B | SAH | 0.946 | 0.857 | 0.353 | 0.995 | 0.53 | 0.5 | 6 | 206 | 1 | 11 | 1 | 1 |
| 2e1t | A | MLC | 0.93 | 0.345 | 0.5 | 0.952 | 0.38 | 0.408 | 10 | 376 | 19 | 10 | 1 | 4 |
| 2e3r | A | 18C | 0.928 | 0.929 | 0.464 | 0.995 | 0.627 | 0.619 | 13 | 192 | 1 | 15 | 1 | 1 |
| 2e5m | A | NAP | 0.95 | 0.5 | 0.222 | 0.988 | 0.311 | 0.308 | 4 | 335 | 4 | 14 | 1 | 2 |
| 2e6u | X | COA | 0 | 0 | 0 | 0 | 0 | 0 | 0 | 99 | 0 | 24 | 1 | 0 |
| 2e9z | A | UTP | 0.93 | 0 | 0 | 0.934 | -0.018 | 0 | 0 | 412 | 29 | 2 | 2 | 6 |
| 2e9z | A | PPV | 0.916 | 0.034 | 0.1 | 0.935 | 0.021 | 0.051 | 1 | 405 | 28 | 9 | 2 | 6 |
| 2ed4 | A | FAD | 0 | 0 | 0 | 0 | 0 | 0 | 0 | 119 | 0 | 24 | 2 | 0 |
| 2ed4 | A | NAD | 0 | 0 | 0 | 0 | 0 | 0 | 0 | 132 | 0 | 11 | 2 | 0 |
| 2f9w | A | PAU | 0.926 | 0.333 | 0.417 | 0.954 | 0.334 | 0.37 | 5 | 207 | 10 | 7 | 1 | 1 |
| 2fa0 | A | HMG | 0.937 | 0.588 | 0.357 | 0.981 | 0.428 | 0.444 | 10 | 361 | 7 | 18 | 1 | 3 |
| 2fhj | A | MFN | 0.883 | 0.286 | 0.286 | 0.936 | 0.222 | 0.286 | 6 | 220 | 15 | 15 | 1 | 1 |
| 2fk8 | A | SAM | 0.918 | 0.519 | 0.667 | 0.942 | 0.544 | 0.583 | 14 | 210 | 13 | 7 | 1 | 2 |
| 2fsg | A | ATP | 0.971 | 0.318 | 0.7 | 0.975 | 0.46 | 0.438 | 7 | 586 | 15 | 3 | 1 | 3 |
| 2fv0 | A | BGC | 0.933 | 0.2 | 0.444 | 0.947 | 0.268 | 0.276 | 4 | 288 | 16 | 5 | 1 | 2 |
| 2fzs | B | CMQ | 0.887 | 0 | 0 | 0.975 | -0.048 | 0 | 0 | 157 | 4 | 16 | 1 | 1 |
| 2g25 | A | TDK | 0.971 | 0.625 | 0.536 | 0.988 | 0.564 | 0.577 | 15 | 717 | 9 | 13 | 1 | 3 |
| 2g97 | A | DGB | 0.94 | 0.375 | 0.5 | 0.961 | 0.402 | 0.429 | 9 | 367 | 15 | 9 | 1 | 2 |
| 2gfx | A | PMN | 0.944 | 0.45 | 0.529 | 0.966 | 0.459 | 0.486 | 9 | 311 | 11 | 8 | 1 | 2 |
| 2gga | A | GPJ | 0.967 | 0.48 | 1 | 0.966 | 0.681 | 0.649 | 12 | 372 | 13 | 0 | 2 | 1 |
| 2gga | A | S3P | 0.97 | 0.52 | 1 | 0.969 | 0.71 | 0.684 | 13 | 372 | 12 | 0 | 2 | 1 |
| 2gj5 | A | VD3 | 0.885 | 0.545 | 0.316 | 0.964 | 0.357 | 0.4 | 6 | 132 | 5 | 13 | 1 | 1 |
| 2gte | A | VA | 0.901 | 1 | 0.478 | 1 | 0.653 | 0.647 | 11 | 98 | 0 | 12 | 1 | 1 |
| 2gwh | A | PCI | 0.909 | 0.333 | 0.917 | 0.909 | 0.521 | 0.489 | 11 | 220 | 22 | 1 | 2 | 1 |
| 2gwh | A | A3P | 0.917 | 0.455 | 0.833 | 0.924 | 0.578 | 0.588 | 15 | 218 | 18 | 3 | 2 | 1 |
| 2gz3 | A | NAP | 0.884 | 0.178 | 1 | 0.881 | 0.396 | 0.302 | 8 | 275 | 37 | 0 | 2 | 2 |
| 2gz3 | A | AS2 | 0.928 | 0.556 | 0.893 | 0.932 | 0.67 | 0.685 | 25 | 272 | 20 | 3 | 2 | 2 |
| 2hbl | A | AMP | 0.975 | 0.375 | 0.429 | 0.986 | 0.388 | 0.4 | 3 | 347 | 5 | 4 | 1 | 2 |
| 2hix | A | ATP | 0.975 | 0.538 | 0.5 | 0.988 | 0.506 | 0.519 | 7 | 503 | 6 | 7 | 1 | 3 |
| 2hl0 | A | A3S | 0.892 | 0.7 | 0.389 | 0.973 | 0.469 | 0.5 | 7 | 109 | 3 | 11 | 1 | 1 |
| 2hob | A | 3IH | 0.927 | 1 | 0.167 | 1 | 0.393 | 0.286 | 4 | 250 | 0 | 20 | 1 | 1 |
| 2hxm | A | 302 | 0.97 | 1 | 0.625 | 1 | 0.778 | 0.769 | 10 | 187 | 0 | 6 | 1 | 1 |
| 2hzq | A | STR | 0.956 | 0.667 | 0.6 | 0.98 | 0.609 | 0.632 | 6 | 147 | 3 | 4 | 1 | 1 |
| 2i4n | B | 5CA | 0.976 | 0.778 | 0.7 | 0.99 | 0.725 | 0.737 | 14 | 392 | 4 | 6 | 1 | 2 |
| 2i56 | A | RNS | 0.957 | 0.385 | 0.909 | 0.958 | 0.576 | 0.541 | 10 | 367 | 16 | 1 | 1 | 3 |
| 2ihz | A | CSF | 0.937 | 0.231 | 0.857 | 0.939 | 0.426 | 0.364 | 6 | 307 | 20 | 1 | 2 | 1 |
| 2ihz | A | LBT | 0.958 | 0.692 | 0.75 | 0.974 | 0.698 | 0.72 | 18 | 302 | 8 | 6 | 2 | 1 |
| 2ioa | A | ADP | 0.929 | 0.314 | 0.44 | 0.953 | 0.336 | 0.367 | 11 | 490 | 24 | 14 | 2 | 3 |
| 2ioa | A | GGA | 0.968 | 0.571 | 0.909 | 0.971 | 0.707 | 0.702 | 20 | 502 | 15 | 2 | 2 | 3 |
| 2irx | A | GTP | 0.939 | 0.5 | 0.688 | 0.955 | 0.555 | 0.579 | 11 | 235 | 11 | 5 | 1 | 2 |
| 2ixl | A | TRH | 0.935 | 0.857 | 0.353 | 0.994 | 0.525 | 0.5 | 6 | 167 | 1 | 11 | 1 | 1 |
| 2j4e | B | ITT | 0.983 | 0.944 | 0.895 | 0.994 | 0.91 | 0.919 | 17 | 159 | 1 | 2 | 1 | 1 |
| 2j72 | B | GLC | 0 | 0 | 0 | 0 | 0 | 0 | 0 | 83 | 0 | 10 | 1 | 0 |
| 2j8y | A | PNM | 0.962 | 0.714 | 0.417 | 0.991 | 0.528 | 0.526 | 5 | 220 | 2 | 7 | 1 | 1 |
| 2jbt | A | FMN | 0.942 | 0.321 | 0.818 | 0.946 | 0.491 | 0.462 | 9 | 335 | 19 | 2 | 2 | 1 |
| 2jbt | A | 4HP | 0.953 | 0.536 | 0.789 | 0.962 | 0.628 | 0.638 | 15 | 333 | 13 | 4 | 2 | 1 |
| 2jgv | D | ADP | 0.923 | 0.3 | 0.429 | 0.949 | 0.319 | 0.353 | 6 | 259 | 14 | 8 | 1 | 1 |
| 2nty | C | GDP | 0.929 | 0.533 | 0.667 | 0.951 | 0.558 | 0.593 | 8 | 135 | 7 | 4 | 1 | 1 |
| 2oal | B | FAD | 0.963 | 0.714 | 0.758 | 0.978 | 0.716 | 0.735 | 25 | 441 | 10 | 8 | 1 | 4 |
| 2oec | A | ANU | 0.921 | 0 | 0 | 0.938 | -0.035 | 0 | 0 | 198 | 13 | 4 | 2 | 1 |
| 2oec | A | K | 0.93 | 0 | 0 | 0.939 | -0.025 | 0 | 0 | 200 | 13 | 2 | 2 | 1 |
| 2ovd | A | DAO | 0.936 | 0.474 | 1 | 0.932 | 0.665 | 0.643 | 9 | 138 | 10 | 0 | 1 | 1 |
| 2pkn | A | ACP | 0.912 | 0.357 | 0.556 | 0.935 | 0.401 | 0.435 | 10 | 260 | 18 | 8 | 1 | 2 |
| 2q6v | A | UDP | 0.92 | 0.364 | 0.706 | 0.932 | 0.47 | 0.48 | 12 | 287 | 21 | 5 | 1 | 2 |
| 2q71 | A | CP3 | 0.959 | 0.667 | 0.762 | 0.973 | 0.691 | 0.711 | 16 | 287 | 8 | 5 | 1 | 3 |
| 2qeh | A | SRO | 0.917 | 1 | 0.083 | 1 | 0.276 | 0.154 | 1 | 121 | 0 | 11 | 1 | 1 |
| 2qo9 | A | ANP | 0.96 | 0.538 | 0.636 | 0.975 | 0.565 | 0.583 | 7 | 234 | 6 | 4 | 1 | 2 |
| 2qtt | A | ADE | 0.965 | 0.571 | 0.8 | 0.972 | 0.659 | 0.667 | 8 | 212 | 6 | 2 | 1 | 2 |
| 2qv7 | A | ADP | 0.963 | 0.688 | 0.688 | 0.981 | 0.668 | 0.688 | 11 | 252 | 5 | 5 | 1 | 2 |
| 2qwi | A | G20 | 0.947 | 0.2 | 0.062 | 0.988 | 0.09 | 0.095 | 1 | 339 | 4 | 15 | 1 | 2 |
| 2qyq | A | PTR | 0.975 | 0.714 | 1 | 0.974 | 0.834 | 0.833 | 10 | 147 | 4 | 0 | 1 | 1 |
| 2qzz | A | NAP | 0.917 | 0.394 | 0.812 | 0.924 | 0.53 | 0.531 | 13 | 242 | 20 | 3 | 2 | 2 |
| 2qzz | A | EMF | 0.95 | 0.697 | 0.852 | 0.96 | 0.743 | 0.767 | 23 | 241 | 10 | 4 | 2 | 2 |
| 2r68 | A | SUP | 0.955 | 0.25 | 0.25 | 0.977 | 0.227 | 0.25 | 3 | 380 | 9 | 9 | 1 | 2 |
| 2r7a | A | HEM | 0.941 | 0.714 | 0.5 | 0.981 | 0.568 | 0.588 | 10 | 212 | 4 | 10 | 1 | 1 |
| 2rfh | A | 23N | 0.936 | 0.414 | 1 | 0.933 | 0.621 | 0.585 | 12 | 238 | 17 | 0 | 1 | 2 |
| 2rjc | A | MES | 0.92 | 0.278 | 0.312 | 0.954 | 0.253 | 0.294 | 5 | 272 | 13 | 11 | 1 | 4 |
| 2rk2 | A | NAP | 0 | 0 | 0 | 0 | 0 | 0 | 0 | 47 | 0 | 6 | 1 | 0 |
| 2rkm | A | LYS | 0.941 | 0.324 | 0.75 | 0.947 | 0.469 | 0.453 | 12 | 448 | 25 | 4 | 1 | 3 |
| 2roy | A | P28 | 0 | 0 | 0 | 0 | 0 | 0 | 0 | 105 | 0 | 10 | 1 | 0 |
| 2sim | A | DAN | 0.979 | 0.722 | 0.867 | 0.985 | 0.781 | 0.788 | 13 | 318 | 5 | 2 | 1 | 2 |
| 2uyq | A | SAM | 0.922 | 0.333 | 0.5 | 0.945 | 0.368 | 0.4 | 6 | 206 | 12 | 6 | 1 | 2 |
| 2v8l | A | GLC | 0.907 | 0 | 0 | 0.978 | -0.04 | 0 | 0 | 88 | 2 | 7 | 1 | 1 |
| 2vaq | A | VAW | 0.944 | 0.5 | 0.6 | 0.964 | 0.518 | 0.545 | 9 | 243 | 9 | 6 | 1 | 1 |
| 2var | A | ANP | 0.922 | 0.278 | 0.385 | 0.949 | 0.286 | 0.323 | 5 | 242 | 13 | 8 | 4 | 1 |
| 2var | A | AMP | 0.922 | 0.278 | 0.385 | 0.949 | 0.286 | 0.323 | 5 | 242 | 13 | 8 | 4 | 1 |
| 2var | A | KDG | 0.963 | 0.556 | 0.833 | 0.969 | 0.663 | 0.667 | 10 | 248 | 8 | 2 | 4 | 1 |
| 2var | A | KDF | 0.978 | 0.833 | 0.833 | 0.988 | 0.821 | 0.833 | 15 | 247 | 3 | 3 | 4 | 1 |
| 2vcj | A | 2EQ | 0.938 | 0.632 | 0.706 | 0.96 | 0.633 | 0.667 | 12 | 168 | 7 | 5 | 1 | 1 |
| 2vfc | A | COA | 0.954 | 0.944 | 0.607 | 0.996 | 0.736 | 0.739 | 17 | 232 | 1 | 11 | 1 | 1 |
| 2vfl | A | C5P | 0.941 | 0.333 | 0.375 | 0.966 | 0.323 | 0.353 | 3 | 171 | 6 | 5 | 1 | 2 |
| 2vkm | A | BSD | 0.972 | 0.815 | 0.815 | 0.985 | 0.8 | 0.815 | 22 | 331 | 5 | 5 | 1 | 1 |
| 2wn7 | A | NAD | 0.952 | 0.4 | 0.125 | 0.991 | 0.205 | 0.19 | 2 | 338 | 3 | 14 | 1 | 1 |
| 2wva | A | TPU | 0.954 | 0.5 | 0.652 | 0.969 | 0.548 | 0.566 | 15 | 464 | 15 | 8 | 1 | 3 |
| 2wzm | A | NA7 | 0.924 | 0.5 | 0.684 | 0.944 | 0.545 | 0.578 | 13 | 218 | 13 | 6 | 1 | 1 |
| 2x60 | A | GTP | 0.96 | 0.645 | 0.952 | 0.961 | 0.765 | 0.769 | 20 | 270 | 11 | 1 | 1 | 2 |
| 2yw2 | A | PO4 | 0.906 | 0.146 | 1 | 0.904 | 0.364 | 0.255 | 6 | 331 | 35 | 0 | 2 | 2 |
| 2yw2 | A | ATP | 0.914 | 0.317 | 0.765 | 0.921 | 0.457 | 0.448 | 13 | 327 | 28 | 4 | 2 | 2 |
| 2yw9 | D | NAP | 0.901 | 1 | 0.2 | 1 | 0.424 | 0.333 | 5 | 177 | 0 | 20 | 1 | 1 |
| 2ywc | A | XMP | 0.924 | 0.074 | 0.182 | 0.942 | 0.081 | 0.105 | 2 | 409 | 25 | 9 | 1 | 3 |
| 2yyu | B | C5P | 0.946 | 0.556 | 0.769 | 0.958 | 0.627 | 0.645 | 10 | 184 | 8 | 3 | 1 | 1 |
| 2z09 | A | ACP | 0.935 | 1 | 0.682 | 1 | 0.794 | 0.811 | 15 | 86 | 0 | 7 | 1 | 1 |
| 2z0x | A | 5CA | 0.866 | 0.4 | 0.111 | 0.976 | 0.157 | 0.174 | 2 | 121 | 3 | 16 | 1 | 1 |
| 2z1s | A | CTT | 0.965 | 0.652 | 0.714 | 0.979 | 0.664 | 0.682 | 15 | 371 | 8 | 6 | 1 | 2 |
| 2za1 | A | OMP | 0.958 | 0.75 | 0.632 | 0.984 | 0.666 | 0.686 | 12 | 242 | 4 | 7 | 1 | 1 |
| 2zas | A | 1OH | 0.928 | 0 | 0 | 0.99 | -0.025 | 0 | 0 | 205 | 2 | 14 | 1 | 1 |
| 2zdq | A | ATP | 0.923 | 0.379 | 0.733 | 0.934 | 0.493 | 0.5 | 11 | 254 | 18 | 4 | 2 | 2 |
| 2zdq | A | DAL | 0.969 | 0.724 | 0.955 | 0.97 | 0.816 | 0.824 | 21 | 257 | 8 | 1 | 2 | 2 |
| 2zgm | B | LAT | 0.966 | 1 | 0.375 | 1 | 0.602 | 0.545 | 3 | 141 | 0 | 5 | 1 | 1 |
| 2zgz | A | GNP | 0.954 | 0.636 | 0.737 | 0.97 | 0.661 | 0.683 | 14 | 258 | 8 | 5 | 1 | 1 |
| 2zhz | B | ATP | 0.906 | 0 | 0 | 0.986 | -0.034 | 0 | 0 | 144 | 2 | 13 | 1 | 1 |
| 2zja | A | ACP | 0.948 | 0.278 | 0.667 | 0.956 | 0.409 | 0.392 | 10 | 559 | 26 | 5 | 1 | 4 |
| 2zu3 | A | ZU3 | 0.939 | 0.889 | 0.471 | 0.993 | 0.621 | 0.615 | 8 | 146 | 1 | 9 | 1 | 1 |
| 3a0t | A | ADP | 0.931 | 0.9 | 0.5 | 0.992 | 0.64 | 0.643 | 9 | 126 | 1 | 9 | 1 | 1 |
| 3a2s | X | SUC | 0.964 | 0.167 | 0.125 | 0.985 | 0.126 | 0.143 | 1 | 322 | 5 | 7 | 1 | 3 |
| 3a5r | A | HC4 | 0.961 | 0.5 | 0.692 | 0.972 | 0.569 | 0.581 | 9 | 308 | 9 | 4 | 1 | 1 |
| 3aaq | A | ARU | 0.946 | 0.583 | 0.7 | 0.964 | 0.61 | 0.636 | 14 | 265 | 10 | 6 | 1 | 1 |
| 3adp | A | NAI | 0.961 | 1 | 0.522 | 1 | 0.708 | 0.686 | 12 | 262 | 0 | 11 | 1 | 1 |
| 3b3f | A | SAH | 0.958 | 0.643 | 0.857 | 0.965 | 0.721 | 0.735 | 18 | 278 | 10 | 3 | 1 | 2 |
| 3b4y | A | FLC | 0.918 | 0.154 | 0.571 | 0.926 | 0.267 | 0.242 | 4 | 275 | 22 | 3 | 2 | 2 |
| 3b4y | A | F42 | 0.947 | 0.654 | 0.708 | 0.968 | 0.652 | 0.68 | 17 | 271 | 9 | 7 | 2 | 2 |
| 3b6a | A | ZCT | 0.908 | 0.571 | 0.2 | 0.984 | 0.301 | 0.296 | 4 | 184 | 3 | 16 | 1 | 1 |
| 3b6r | A | ADP | 0.956 | 0 | 0 | 0 | 0 | 0 | 0 | 325 | 15 | 0 | 3 | 3 |
| 3b6r | A | CRN | 0.956 | 0 | 0 | 0 | 0 | 0 | 0 | 325 | 15 | 0 | 3 | 3 |
| 3b6r | A | NO3 | 0.956 | 0 | 0 | 0 | 0 | 0 | 0 | 325 | 15 | 0 | 3 | 3 |
| 3baz | A | NAP | 0.96 | 0.759 | 0.846 | 0.972 | 0.779 | 0.8 | 22 | 244 | 7 | 4 | 1 | 2 |
| 3bts | B | NAD | 0.897 | 0.143 | 0.136 | 0.947 | 0.085 | 0.14 | 3 | 319 | 18 | 19 | 1 | 2 |
| 3byn | A | RAF | 0.985 | 0.875 | 0.583 | 0.997 | 0.707 | 0.7 | 7 | 377 | 1 | 5 | 1 | 1 |
| 3c2f | A | PRP | 0.939 | 0.333 | 0.875 | 0.941 | 0.518 | 0.483 | 7 | 224 | 14 | 1 | 1 | 1 |
| 3cag | A | ARG | 0 | 0 | 0 | 0 | 0 | 0 | 0 | 54 | 0 | 14 | 1 | 0 |
| 3cgy | A | RDC | 0 | 0 | 0 | 0 | 0 | 0 | 0 | 110 | 0 | 12 | 1 | 0 |
| 3cq3 | A | GOL | 0 | 0 | 0 | 0 | 0 | 0 | 0 | 82 | 0 | 11 | 1 | 0 |
| 3crr | A | DPO | 0.926 | 0.304 | 1 | 0.923 | 0.53 | 0.467 | 7 | 193 | 16 | 0 | 1 | 1 |
| 3ct5 | A | NAG | 0 | 0 | 0 | 0 | 0 | 0 | 0 | 112 | 0 | 13 | 1 | 0 |
| 3cwk | A | REA | 0.898 | 0.667 | 0.375 | 0.973 | 0.45 | 0.48 | 6 | 109 | 3 | 10 | 1 | 1 |
| 3cxi | A | VIT | 0.802 | 0.167 | 0.059 | 0.944 | 0.004 | 0.087 | 1 | 84 | 5 | 16 | 1 | 2 |
| 3d1g | A | 322 | 0.976 | 1 | 0.333 | 1 | 0.57 | 0.5 | 4 | 327 | 0 | 8 | 1 | 1 |
| 3d4p | A | PYR | 0.916 | 0.222 | 0.857 | 0.918 | 0.411 | 0.353 | 6 | 235 | 21 | 1 | 2 | 2 |
| 3d4p | A | NAD | 0.954 | 0.778 | 0.778 | 0.975 | 0.752 | 0.778 | 21 | 230 | 6 | 6 | 2 | 2 |
| 3dsr | A | ADP | 0.951 | 0 | 0 | 0.979 | -0.025 | 0 | 0 | 365 | 8 | 11 | 1 | 1 |
| 3du4 | A | PLP | 0.949 | 0.294 | 0.385 | 0.969 | 0.311 | 0.333 | 5 | 371 | 12 | 8 | 2 | 1 |
| 3du4 | A | KAP | 0.972 | 0.588 | 0.714 | 0.982 | 0.634 | 0.645 | 10 | 375 | 7 | 4 | 2 | 1 |
| 3duw | A | SAH | 0.94 | 0.722 | 0.65 | 0.972 | 0.652 | 0.684 | 13 | 175 | 5 | 7 | 1 | 2 |
| 3dwr | A | 0PA | 0.887 | 0.346 | 0.391 | 0.932 | 0.306 | 0.367 | 9 | 234 | 17 | 14 | 1 | 2 |
| 3dzl | B | 3OC | 0.947 | 0.737 | 0.824 | 0.963 | 0.749 | 0.778 | 14 | 129 | 5 | 3 | 1 | 1 |
| 3e3s | A | I3C | 0.925 | 0.5 | 0.231 | 0.981 | 0.306 | 0.316 | 3 | 158 | 3 | 10 | 1 | 1 |
| 3efv | A | NAD | 0.943 | 0.516 | 0.64 | 0.962 | 0.545 | 0.571 | 16 | 381 | 15 | 9 | 1 | 1 |
| 3egv | A | SAH | 0.964 | 0.68 | 1 | 0.961 | 0.809 | 0.81 | 17 | 199 | 8 | 0 | 1 | 1 |
| 3ej0 | A | 11X | 0.848 | 0 | 0 | 0.905 | -0.081 | 0 | 0 | 134 | 14 | 10 | 1 | 2 |
| 3ek5 | A | GTP | 0.824 | 0 | 0 | 0.875 | -0.091 | 0 | 0 | 182 | 26 | 13 | 1 | 1 |
| 3erg | A | GTS | 0.973 | 0.875 | 0.824 | 0.988 | 0.834 | 0.848 | 14 | 169 | 2 | 3 | 1 | 1 |
| 3erk | A | SB4 | 0.957 | 0.385 | 0.455 | 0.974 | 0.396 | 0.417 | 5 | 305 | 8 | 6 | 1 | 2 |
| 3ert | A | OHT | 0.959 | 1 | 0.571 | 1 | 0.739 | 0.727 | 12 | 197 | 0 | 9 | 1 | 1 |
| 3exs | A | 5RP | 0.918 | 0.444 | 1 | 0.912 | 0.637 | 0.615 | 12 | 156 | 15 | 0 | 1 | 2 |
| 3f1k | A | NAP | 0.932 | 0.688 | 0.786 | 0.952 | 0.697 | 0.733 | 22 | 197 | 10 | 6 | 1 | 3 |
| 3f47 | A | I2C | 0.936 | 0.25 | 0.75 | 0.941 | 0.41 | 0.375 | 6 | 285 | 18 | 2 | 2 | 2 |
| 3f47 | A | CMO | 0.945 | 0.542 | 0.684 | 0.962 | 0.58 | 0.605 | 13 | 281 | 11 | 6 | 2 | 2 |
| 3ftf | A | SAH | 0.923 | 0.647 | 0.478 | 0.972 | 0.516 | 0.55 | 11 | 205 | 6 | 12 | 1 | 2 |
| 3fwr | A | ADP | 0.904 | 0.6 | 0.4 | 0.967 | 0.44 | 0.48 | 6 | 117 | 4 | 9 | 1 | 1 |
| 3g1x | A | U | 0.944 | 0.68 | 0.85 | 0.954 | 0.73 | 0.756 | 17 | 167 | 8 | 3 | 1 | 1 |
| 3gdl | A | UP6 | 0.964 | 0.773 | 0.85 | 0.975 | 0.791 | 0.81 | 17 | 196 | 5 | 3 | 1 | 1 |
| 3gid | A | S1A | 0.926 | 0.15 | 0.176 | 0.958 | 0.124 | 0.162 | 3 | 384 | 17 | 14 | 1 | 3 |
| 3gpl | A | ANP | 0.957 | 0.438 | 0.875 | 0.96 | 0.601 | 0.583 | 14 | 434 | 18 | 2 | 1 | 2 |
| 3gpo | A | APR | 0.93 | 0.929 | 0.591 | 0.992 | 0.707 | 0.722 | 13 | 119 | 1 | 9 | 1 | 1 |
| 3gqk | A | ATP | 0 | 0 | 0 | 0 | 0 | 0 | 0 | 139 | 0 | 12 | 1 | 0 |
| 3h18 | A | PMS | 0.971 | 0.722 | 0.867 | 0.978 | 0.776 | 0.788 | 13 | 222 | 5 | 2 | 1 | 1 |
| 3h2k | A | BOG | 0.96 | 0.941 | 0.552 | 0.997 | 0.704 | 0.696 | 16 | 321 | 1 | 13 | 1 | 1 |
| 3h39 | A | ATP | 0.977 | 0.7 | 0.538 | 0.992 | 0.602 | 0.609 | 7 | 374 | 3 | 6 | 1 | 2 |
| 3h72 | A | SIA | 0.983 | 0.7 | 0.933 | 0.985 | 0.8 | 0.8 | 14 | 402 | 6 | 1 | 1 | 1 |
| 3hit | A | DYN | 0.941 | 1 | 0.364 | 1 | 0.584 | 0.533 | 8 | 215 | 0 | 14 | 1 | 2 |
| 3hiy | A | UTP | 0.972 | 0.615 | 1 | 0.97 | 0.773 | 0.762 | 16 | 326 | 10 | 0 | 1 | 1 |
| 3hp8 | A | SUC | 0 | 0 | 0 | 0 | 0 | 0 | 0 | 76 | 0 | 22 | 1 | 0 |
| 3hpi | A | SUC | 0.977 | 0.647 | 0.846 | 0.982 | 0.729 | 0.733 | 11 | 331 | 6 | 2 | 1 | 2 |
| 3hvl | A | SRL | 0.948 | 0.706 | 0.571 | 0.98 | 0.608 | 0.632 | 12 | 242 | 5 | 9 | 1 | 2 |
| 3hvo | A | VGI | 0.965 | 0.214 | 0.3 | 0.978 | 0.236 | 0.25 | 3 | 498 | 11 | 7 | 1 | 3 |
| 3i0d | A | UDP | 0.941 | 0.286 | 0.5 | 0.957 | 0.35 | 0.364 | 4 | 221 | 10 | 4 | 1 | 1 |
| 3i6c | A | GIA | 0.84 | 0.5 | 0.125 | 0.976 | 0.189 | 0.2 | 2 | 82 | 2 | 14 | 1 | 1 |
| 3i8x | A | GDP | 0.979 | 0.9 | 0.692 | 0.996 | 0.779 | 0.783 | 9 | 224 | 1 | 4 | 1 | 1 |
| 3ido | A | EPE | 0.971 | 0.8 | 0.8 | 0.984 | 0.784 | 0.8 | 8 | 125 | 2 | 2 | 1 | 1 |
| 3ies | A | M24 | 0.966 | 0.706 | 0.889 | 0.971 | 0.774 | 0.787 | 24 | 340 | 10 | 3 | 1 | 2 |
| 3iiq | A | JZA | 0.931 | 0.481 | 0.722 | 0.946 | 0.555 | 0.578 | 13 | 244 | 14 | 5 | 1 | 1 |
| 3iiq | A | ARY | 0.974 | 0.833 | 0.556 | 0.995 | 0.669 | 0.667 | 5 | 186 | 1 | 4 | 1 | 1 |
| 3jyn | A | NDP | 0.912 | 0.541 | 0.69 | 0.936 | 0.562 | 0.606 | 20 | 248 | 17 | 9 | 1 | 2 |
| 3kak | A | 3GC | 0.939 | 0 | 0 | 0.962 | -0.031 | 0 | 0 | 384 | 15 | 10 | 1 | 3 |
| 3kbn | A | GLO | 0.961 | 0.481 | 1 | 0.959 | 0.68 | 0.65 | 13 | 330 | 14 | 0 | 1 | 2 |
| 3kdi | A | A8S | 0.943 | 0.632 | 0.8 | 0.957 | 0.681 | 0.706 | 12 | 154 | 7 | 3 | 1 | 1 |
| 3kjg | A | ADP | 0.937 | 0.522 | 0.8 | 0.947 | 0.615 | 0.632 | 12 | 197 | 11 | 3 | 1 | 1 |
| 3kp5 | A | KAN | 0 | 0 | 0 | 0 | 0 | 0 | 0 | 122 | 0 | 11 | 1 | 0 |
| 3ku1 | A | SAM | 0.938 | 0.8 | 0.444 | 0.989 | 0.568 | 0.571 | 8 | 174 | 2 | 10 | 1 | 1 |
| 3kv8 | A | FAH | 0.887 | 0.75 | 0.333 | 0.981 | 0.451 | 0.462 | 6 | 104 | 2 | 12 | 1 | 1 |
| 3lbz | A | Z88 | 0 | 0 | 0 | 0 | 0 | 0 | 0 | 109 | 0 | 3 | 2 | 0 |
| 3lbz | A | Z89 | 0 | 0 | 0 | 0 | 0 | 0 | 0 | 103 | 0 | 9 | 2 | 0 |
| 3ldk | A | SUC | 0.962 | 0.371 | 0.929 | 0.963 | 0.574 | 0.531 | 13 | 566 | 22 | 1 | 1 | 5 |
| 3ll3 | A | DXP | 0.879 | 0.02 | 0.143 | 0.89 | 0.013 | 0.035 | 1 | 398 | 49 | 6 | 3 | 4 |
| 3ll3 | A | ATP | 0.892 | 0.12 | 0.545 | 0.901 | 0.219 | 0.197 | 6 | 399 | 44 | 5 | 3 | 4 |
| 3ll3 | A | XUL | 0.901 | 0.14 | 0.778 | 0.903 | 0.303 | 0.237 | 7 | 402 | 43 | 2 | 3 | 4 |
| 3lzz | A | GDP | 0.907 | 0.353 | 0.667 | 0.922 | 0.441 | 0.462 | 6 | 130 | 11 | 3 | 1 | 2 |
| 3m4e | A | BCD | 0.982 | 0 | 0 | 0.996 | -0.007 | 0 | 0 | 270 | 1 | 4 | 1 | 1 |
| 3maq | A | DGT | 0.955 | 0.275 | 0.786 | 0.958 | 0.449 | 0.407 | 11 | 668 | 29 | 3 | 1 | 5 |
| 3nk7 | A | SAM | 0 | 0 | 0 | 0 | 0 | 0 | 0 | 224 | 0 | 19 | 1 | 0 |
| 3std | A | MQ0 | 0.953 | 0.929 | 0.684 | 0.992 | 0.774 | 0.788 | 13 | 130 | 1 | 6 | 1 | 1 |
| 3ts1 | A | TYA | 0.936 | 0.607 | 0.739 | 0.954 | 0.635 | 0.667 | 17 | 230 | 11 | 6 | 1 | 4 |
| 3w7f | A | FPS | 0.928 | 0.679 | 0.655 | 0.962 | 0.627 | 0.667 | 19 | 227 | 9 | 10 | 1 | 2 |
| 4dfr | A | MTX | 0.871 | 0.476 | 0.588 | 0.911 | 0.456 | 0.526 | 10 | 112 | 11 | 7 | 1 | 2 |
| 4std | A | BFS | 0 | 0 | 0 | 0 | 0 | 0 | 0 | 134 | 0 | 18 | 1 | 0 |
| 5std | A | UNN | 0.967 | 1 | 0.762 | 1 | 0.857 | 0.865 | 16 | 130 | 0 | 5 | 1 | 1 |
| 5tln | A | BAN | 0.983 | 0.812 | 0.867 | 0.989 | 0.83 | 0.839 | 13 | 268 | 3 | 2 | 1 | 1 |
| 7dfr | A | NAP | 0.848 | 0.632 | 0.462 | 0.938 | 0.453 | 0.533 | 12 | 105 | 7 | 14 | 1 | 2 |
| 830c | A | RS1 | 0.917 | 1 | 0.368 | 1 | 0.58 | 0.538 | 7 | 125 | 0 | 12 | 1 | 1 |
| 966c | A | RS2 | 0.906 | 1 | 0.263 | 1 | 0.487 | 0.417 | 5 | 130 | 0 | 14 | 1 | 1 |
|  |  | Total | 0.943 | 0.513 | 0.562 | 0.967 | 0.507 | 0.536 | 4557 | 126018 | 4324 | 3557 |  |  |
