# Supplementary material for: Predicting Ligand Binding Sites on Protein Surfaces by 3-Dimensional Probability Density Distributions of Interacting Atoms
Source: PLoS One. 2016 Aug 11;11(8):e0160315. doi: 10.1371/journal.pone.0160315 (PMC4981321; doi:10.1371/journal.pone.0160315)
Supplement: S6 Table — (DOCX) [file pone.0160315.s007.docx]

**S6 Table. Comparison of the top 1 predicted success rates of ISMBLab-LIG with those of various ligand binding site predictors on the S48 bound/unbound dataset ^a^.**

| Methods | S48b | S48ub |
| --- | --- | --- |
|  | Top 1 (%) | Top 1 (%) |
| **ISMBLab-LIG** | **85** | **83** |
| LISE (Xie et al., 2012)^b^ | 92 | 81 |
| MPK2 (Zhang et al., 2011)^b^ | 85 | 80 |
| VICE (Tripathi and Kellogg, 2010)^c^ | 85 | 83 |
| MPK1 (Huang, 2009)^b^ | 83 | 75 |
| DoGSite (Volkamer et al., 2010)^b^ | 83 | 71 |
| Fpocket (Le Guilloux et al., 2009)^b^ | 83 | 69 |
| LIGSITE^cs^ (Huang and Schroeder, 2006)^b^ | 81 | 71 |
| LIGSITE^csc^ (Huang and Schroeder, 2006)^b^ | 79 | 71 |
| MSPocket (Zhu and Pisabarro, 2010)^b^ | 77 | 75 |
| POCASA (Yu et al., 2010)^b^ | 77 | 75 |
| Q-SiteFinder (Laurie and Jackson, 2005)^c^ | 75 | 52 |
| PocketPicker (Weisel et al., 2007)^b^ | 72 | 69 |
| CAST (Liang et al., 1998)^c^ | 67 | 58 |
| PASS (Brady and Stouten, 2000)^c^ | 63 | 60 |
| SURFNET (Laskowski, 1995)^c^ | 54 | 52 |

^a^The success rates were calculated for the 48 bound or unbound structures for which the top 1 predicted binding site satisfied the 4Å distance criterion (see methods section).

^b^Data were taken from [1]

^c^Data reported [2,3].

1. Xie ZR, Hwang MJ. Ligand-binding site prediction using ligand-interacting and binding site-enriched protein triangles. Bioinformatics. 2012;28(12):1579-85.

2. Huang B. MetaPocket: a meta approach to improve protein ligand binding site prediction. Omics : a journal of integrative biology. 2009;13(4):325-30. Epub 2009/08/04.

3. Huang B, Schroeder M. LIGSITEcsc: predicting ligand binding sites using the Connolly surface and degree of conservation. BMC structural biology. 2006;6:19.
