# Supplementary material for: Predicting Ligand Binding Sites on Protein Surfaces by 3-Dimensional Probability Density Distributions of Interacting Atoms
Source: PLoS One. 2016 Aug 11;11(8):e0160315. doi: 10.1371/journal.pone.0160315 (PMC4981321; doi:10.1371/journal.pone.0160315)
Supplement: S7 Table — (DOCX) [file pone.0160315.s008.docx]

**S7 Table*.* Comparisons of the top 1 prediction success rates of ISMBLab-LIG with those of various ligand binding site prediction methods on the S210 dataset^a^**

| Methods | Top 1 (%) |
| --- | --- |
| **ISMBLab-LIG** | **84** |
| LISE (Xie et al., 2012)^b^ | 83 |
| MPK2 (Zhang et al., 2011)^b^ | 81 |
| MPK1 (Huang, 2009)^b^ | 75 |
| Q-SiteFinder (Laurie and Jackson, 2005)^c^ | 70 |
| LIGSITEcsc (Huang and Schroeder, 2006)^b^ | 75 |
| LIGSITEcs (Huang and Schroeder, 2006)^b^ | 70 |
| PASS (Brady and Stouten, 2000)^c^ | 51 |
| SURFNET (Laskowski, 1995)^c^ | 42 |

^a^The success rates were calculated for the S210 data set for which the top 1 predicted binding site satisfied the 4Å distance criterion (see methods section).

^b^Data were taken from [1]

^c^Data reported [2, 3].

1. Xie ZR, Hwang MJ. Ligand-binding site prediction using ligand-interacting and binding site-enriched protein triangles. Bioinformatics. 2012;28(12):1579-85.

2. Huang B. MetaPocket: a meta approach to improve protein ligand binding site prediction. Omics : a journal of integrative biology. 2009;13(4):325-30. Epub 2009/08/04.

3. Huang B, Schroeder M. LIGSITEcsc: predicting ligand binding sites using the Connolly surface and degree of conservation. BMC structural biology. 2006;6:19.
