# Supplementary material for: Predicting Ligand Binding Sites on Protein Surfaces by 3-Dimensional Probability Density Distributions of Interacting Atoms
Source: PLoS One. 2016 Aug 11;11(8):e0160315. doi: 10.1371/journal.pone.0160315 (PMC4981321; doi:10.1371/journal.pone.0160315)
Supplement: S8 Table — (DOCX) [file pone.0160315.s009.docx]

**S8 Table. Comparisons of the top 1 prediction success rates of ISMBLab-LIG with those of various ligand binding site prediction methods on the S198 dataset.**

| Methods | Top 1 (%) |
| --- | --- |
| **ISMBLab-LIG** | **55** |
| **MPK2** (Zhang et al., 2011) ^b^ | **61** |
| MPK1 (Huang, 2009)^b^ | 55 |
| LIGSITE^CS^ (Huang and Schroeder, 2006)^b^ | 48 |
| ConCavity (Capra et al., 2009)^b^ | 47 |
| POCASA (Yu et al., 2010)^b^ | 43 |
| Q-SiteFinder (Laurie and Jackson, 2005)^b^ | 40 |
| GHECOM (Kawabata, 2007)^b^ | 39 |
| PASS (Brady and Stouten, 2000)^b^ | 35 |
| SURFNET (Laskowski, 1995)^b^ | 24 |
| Fpocket (Le Guilloux et al., 2009)^b^ | 31 |

^a^The success rates were calculated for the S198 data set for which the top 1 predicted binding site satisfied the 4Å distance criterion (see methods section).

^b^Data were taken from [1].

1. Zhang ZM, Li Y, Lin BY, Schroeder M, Huang BD. Identification of cavities on protein surface using multiple computational approaches for drug binding site prediction. Bioinformatics. 2011;27(15):2083-8.
