# Supplementary material for: Predicting Ligand Binding Sites on Protein Surfaces by 3-Dimensional Probability Density Distributions of Interacting Atoms
Source: PLoS One. 2016 Aug 11;11(8):e0160315. doi: 10.1371/journal.pone.0160315 (PMC4981321; doi:10.1371/journal.pone.0160315)
Supplement: S9 Table — Columns 1 and 2 show the PDB code name and chain name respectively for the query protein sequence to be comparatively modeled with MODELLER. Column 3 shows the PDB code name of the template structure used in the comparative modeling. Columns 4 and 5 show the sequence ID (%) and alignment coverage fraction between the query sequence and the template structure. Column 6 shows the root mean square deviation (RMSD) between the ligand-neighboring atoms in the actual structure (shown in column 1) and the corresponding atoms in the modeled structure. The ligand-neighboring atoms are the atoms within 10 Å to the ligand-binding atoms, which are within 4.5 Å to any of the heavy atom in the corresponding ligand. Column 7 shows the distance from the geometry center of the predicted top one ligand binding patch (see Methods in the man text) on the actual structure (column 1) to the geometry center of the predicted top one ligand binding patch on the comparatively modeled structure based on the template structure (column3). The actual structure and the modeled structure were superimposed to optimize the RMSD of corresponding backbone Cα before calculating the distance between the predicted binding center. Column 8 indicates the examples (A)~(E) shown in Fig 5 of the main text. (DOCX) [file pone.0160315.s010.docx]

**S9 Table. Details of model template and distances of predicted binding centers between ligand-bound structures and modeled structures. Columns 1 and 2 show the PDB code name and chain name respectively for the query protein sequence to be comparatively modeled with MODELLER. Column 3 shows the PDB code name of the template structure used in the comparative modeling.** **Columns 4 and 5 show the sequence ID (%) and alignment coverage fraction between the query sequence and the template structure. Column 6 shows the root mean square deviation (RMSD) between the ligand-neighboring atoms in the actual structure (shown in column 1) and the corresponding atoms in the modeled structure. The ligand-neighboring atoms are the atoms within 10 Å to the ligand-binding atoms, which are within 4.5 Å to any of the heavy atom in the corresponding ligand. Column 7 shows the distance from the geometry center of the predicted top one ligand binding patch (see Methods in the man text) on the actual structure (column 1) to the geometry center of the predicted top one ligand binding patch on the comparatively modeled structure based on the template structure (column3). The actual structure and the modeled structure were superimposed to optimize the RMSD of corresponding backbone Cα before calculating the distance between the predicted binding center. Column 8 indicates the examples (A)~(E) shown in Figure 5 of the main text.**

| PDB ID | Chain ID | PDB ID of template | Seq ID (%) | Seq Coverage | RMSD | Dist | Fig |
| --- | --- | --- | --- | --- | --- | --- | --- |
| 1okm | A | 1KEQ | 54.17 | 0.93 | 13.27 | 3.46 | A |
| 2sim | A | 1WCS | 28.75 | 0.95 | 12.01 | 1.21 | B |
| 1mtw | A | 4F4O | 28.29 | 1.00 | 10.29 | 1.53 | C |
| 3gch | A | 1ELV | 33.96 | 1.00 | 10.13 | 3.83 | D |
| 1qpe | A | 3BYS | 99.63 | 1.00 | 9.51 | 3.64 | E |
| 1okm | A | 3JXG | 29.7 | 0.99 | 2.89 | 0.50 |  |
| 3gch | A | 1PYT | 41.67 | 0.98 | 4.54 | 0.18 |  |
| 1okm | A | 3IAI | 34.48 | 0.99 | 5.29 | 0.70 |  |
| 3ptb | A | 1FIW | 32.81 | 1.00 | 2.08 | 0.87 |  |
| 1hfc | A | 4H2E | 54.84 | 0.97 | 2.12 | 0.23 |  |
| 3ptb | A | 2ZPS | 64.86 | 0.99 | 4.02 | 0.33 |  |
| 1hyt | A | 3NQX | 33.33 | 0.78 | 5.74 | 1.02 |  |
| 1hew | A | 2GV0 | 68.46 | 0.99 | 1.52 | 0.46 |  |
| 1ulb | A | 1V4N | 24.36 | 0.92 | 4.96 | 0.99 |  |
| 3gch | A | 1RJX | 37.6 | 1.00 | 5.77 | 1.03 |  |
| 1okm | A | 3DA2 | 60.55 | 0.99 | 1.36 | 0.64 |  |
| 1pso | I | 1UH7 | 38.72 | 0.98 | 3.48 | 1.04 |  |
| 1phd | A | 3LXH | 45.19 | 0.98 | 1.67 | 0.67 |  |
| 3ptb | A | 1SGF | 40 | 0.96 | 2.62 | 0.67 |  |
| 1mtw | A | 2ZPS | 64.86 | 0.99 | 3.64 | 0.71 |  |
| 2h4n | A | 1FLJ | 58.98 | 0.99 | 1.64 | 0.72 |  |
| 1rne | A | 1CZI | 37.16 | 0.98 | 7.67 | 1.10 |  |
| 3ptb | A | 1F7Z | 73.99 | 1.00 | 4.83 | 0.29 |  |
| 2tmn | E | 3NQX | 33.33 | 0.78 | 2.50 | 1.74 |  |
| 3ptb | A | 2PSX | 47.14 | 1.00 | 1.75 | 0.79 |  |
| 1fbp | A | 1XI6 | 35.59 | 0.18 | 12.66 | 1.04 |  |
| 3ptb | A | 4F4O | 28.29 | 1.00 | 11.10 | 1.15 |  |
| 1mtw | A | 1FIW | 32.81 | 1.00 | 2.09 | 1.17 |  |
| 1mtw | A | 1SGF | 40 | 0.96 | 2.72 | 0.84 |  |
| 1gca | A | 2RJO | 38.37 | 0.27 | 6.07 | 4.97 |  |
| 1srf | A | 2OFA | 34.15 | 0.99 | 4.92 | 1.19 |  |
| 1cdo | A | 3VPX | 43.75 | 0.12 | 20.82 | 5.89 |  |
| 1mtw | A | 2PSX | 47.14 | 1.00 | 1.71 | 0.85 |  |
| 1bid | A | 3INV | 46.83 | 0.99 | 4.21 | 0.89 |  |
| 2h4n | A | 3IAI | 34.1 | 0.99 | 6.30 | 1.44 |  |
| 1cdo | A | 3JYL | 36.49 | 0.19 | 12.55 | 5.13 |  |
| 1ivd | A | 1V0Z | 51.15 | 0.99 | 2.19 | 0.94 |  |
| 7cpa | A | 1CPB | 35.37 | 0.26 | 10.19 | 5.27 |  |
| 2ifb | A | 1BCP | 40.74 | 0.20 | 17.43 | 6.08 |  |
| 1hfc | A | 1Q3A | 61.39 | 0.98 | 2.48 | 1.02 |  |
| 1mtw | A | 1F7Z | 73.99 | 1.00 | 4.18 | 0.67 |  |
| 1bid | A | 3QJ7 | 66.29 | 1.00 | 1.92 | 1.04 |  |
| 1byb | A | 3OVS | 27.43 | 0.22 | 23.63 | 6.05 |  |
| 1rne | A | 1SMR | 71 | 0.99 | 7.40 | 0.72 |  |
| 2sim | A | 2YA4 | 35.92 | 0.25 | 17.61 | 6.50 |  |
| 1byb | A | 1VEP | 32.26 | 0.86 | 14.00 | 2.56 |  |
| 3ptb | A | 3MYW | 82.96 | 1.00 | 0.84 | 0.74 |  |
| 3ptb | A | 1H9H | 79.65 | 1.00 | 0.98 | 0.77 |  |
| 1hew | A | 1KEC | 27.91 | 0.33 | 14.83 | 6.53 |  |
| 2h4n | A | 1V9I | 79.92 | 0.98 | 1.53 | 0.81 |  |
| 2ctc | A | 1NSA | 48.84 | 0.98 | 1.53 | 1.09 |  |
| 1mtw | A | 3MYW | 82.96 | 1.00 | 0.82 | 0.99 |  |
| 1hfc | A | 1QIB | 55.7 | 0.99 | 2.28 | 1.25 |  |
| 3ptb | A | 1HJ8 | 66.22 | 0.99 | 4.19 | 1.26 |  |
| 2h4n | A | 4HU1 | 60.16 | 0.99 | 1.73 | 1.30 |  |
| 1ulb | A | 2P4S | 55.91 | 0.96 | 2.49 | 1.32 |  |
| 1okm | A | 2HFW | 59.77 | 0.99 | 1.56 | 1.35 |  |
| 1mrg | A | 2VLC | 34.78 | 0.99 | 3.91 | 1.69 |  |
| 1apu | E | 3APR | 40.72 | 1.00 | 4.91 | 1.42 |  |
| 2h4n | A | 1V9E | 80.31 | 0.98 | 1.63 | 1.07 |  |
| 1ulb | A | 1C3X | 35.91 | 0.89 | 3.95 | 2.71 |  |
| 2h4n | A | 1KEQ | 53.75 | 0.93 | 8.81 | 1.48 |  |
| 1bid | A | 1B5D | 24.52 | 0.73 | 20.02 | 3.43 |  |
| 1acj | A | 1B41 | 57.81 | 1.00 | 1.24 | 1.54 |  |
| 1mtw | A | 1HJ8 | 66.22 | 0.99 | 3.78 | 1.57 |  |
| 2tmn | E | 1BQB | 44.97 | 0.98 | 2.10 | 1.67 |  |
| 2ypi | A | 3S6D | 31.65 | 0.83 | 4.68 | 5.10 |  |
| 1qpe | A | 2DQ7 | 69.29 | 0.98 | 1.66 | 1.72 |  |
| 1imb | A | 2NQL | 35.82 | 0.23 | 13.50 | 7.06 |  |
| 7cpa | A | 2BOA | 59.93 | 0.99 | 1.38 | 1.81 |  |
| 1mtw | A | 1H9H | 79.65 | 1.00 | 0.82 | 1.09 |  |
| 1hyt | A | 1BQB | 44.97 | 0.98 | 2.89 | 1.81 |  |
| 2ctc | A | 1AYE | 66.67 | 0.99 | 1.86 | 1.88 |  |
| 2ctc | A | 1CPB | 36.59 | 0.26 | 10.29 | 7.62 |  |
| 1mrg | A | 1NIO | 69.51 | 1.00 | 1.23 | 1.88 |  |
| 1mrg | A | 1UQ4 | 35.95 | 0.92 | 2.87 | 1.73 |  |
| 2sim | A | 2VVZ | 42.86 | 0.18 | 17.40 | 6.27 |  |
| 2ctc | A | 2V77 | 81.43 | 1.00 | 1.08 | 1.44 |  |
| 1apu | E | 1IZD | 67.28 | 1.00 | 1.93 | 1.90 |  |
| 1stp | A | 2OFA | 32.8 | 0.98 | 4.37 | 2.24 |  |
| 1gca | A | 2H3H | 31.18 | 0.29 | 13.20 | 8.02 |  |
| 1blh | A | 3NI9 | 28.02 | 0.98 | 3.67 | 2.25 |  |
| 1fbp | A | 4GX6 | 94.85 | 1.00 | 1.56 | 1.46 |  |
| 1stp | A | 2ZSC | 50.83 | 0.98 | 1.83 | 1.91 |  |
| 1imb | A | 3LUZ | 34.72 | 0.95 | 9.06 | 2.41 |  |
| 1mtw | A | 1L2E | 44 | 1.00 | 2.67 | 1.93 |  |
| 4dfr | A | 2ZZA | 54.72 | 0.99 | 2.22 | 1.99 |  |
| 1acj | A | 1UKC | 28.36 | 0.98 | 11.98 | 2.57 |  |
| 7cpa | A | 3DGV | 44.52 | 0.99 | 2.03 | 2.04 |  |
| 1qpe | A | 2P2H | 38.72 | 0.99 | 12.86 | 2.72 |  |
| 1apu | E | 3PSG | 32.56 | 0.99 | 18.23 | 2.81 |  |
| 1fbp | A | 4IR8 | 28.44 | 0.97 | 7.08 | 2.88 |  |
| 1srf | A | 4JNJ | 59.66 | 0.99 | 4.67 | 2.08 |  |
| 2pk4 | A | 2PF1 | 43.75 | 0.98 | 2.44 | 2.13 |  |
| 1srf | A | 4DNE | 94.96 | 0.99 | 1.08 | 1.54 |  |
| 3ptb | A | 1L2E | 44 | 1.00 | 3.14 | 2.15 |  |
| 2pk4 | A | 1A0H | 39.24 | 0.96 | 3.79 | 2.89 |  |
| 1bid | A | 3UWL | 51.12 | 0.99 | 2.39 | 2.15 |  |
| 1mrg | A | 3BWH | 57.85 | 0.98 | 1.91 | 2.20 |  |
| 4dfr | A | 1ZDR | 40.49 | 0.99 | 2.44 | 2.20 |  |
| 1cdo | A | 1H2B | 27.06 | 0.98 | 5.34 | 3.06 |  |
| 5cna | A | 3RRD | 84.81 | 1.00 | 1.46 | 1.64 |  |
| 1stp | A | 2C1Q | 29.92 | 0.98 | 4.28 | 3.13 |  |
| 1cdo | A | 1N8K | 54.93 | 1.00 | 2.59 | 2.21 |  |
| 7cpa | A | 2V77 | 79.48 | 1.00 | 1.33 | 1.72 |  |
| 2ypi | A | 1BTM | 39.29 | 0.98 | 1.82 | 3.28 |  |
| 2ifb | A | 1SA8 | 79.39 | 0.99 | 13.40 | 1.75 |  |
| 4dfr | A | 2FZJ | 28.65 | 0.99 | 2.68 | 3.35 |  |
| 1bid | A | 1TIS | 44.67 | 1.00 | 4.36 | 2.24 |  |
| 1fbp | A | 1BK4 | 86.36 | 1.00 | 1.65 | 1.87 |  |
| 1bid | A | 4H0R | 64.75 | 0.98 | 5.63 | 2.39 |  |
| 1hfc | A | 1RM8 | 49.4 | 0.97 | 1.97 | 2.39 |  |
| 1cdo | A | 3QJ5 | 61.23 | 0.99 | 2.85 | 2.45 |  |
| 1hyt | A | 4GER | 55.81 | 0.97 | 1.50 | 2.46 |  |
| 1stp | A | 4JNJ | 58.87 | 0.99 | 4.94 | 2.62 |  |
| 2pk4 | A | 1KIV | 84.62 | 0.96 | 2.02 | 1.94 |  |
| 1okm | A | 4LU3 | 36.5 | 0.99 | 2.68 | 3.36 |  |
| 1imb | A | 2FVZ | 53.31 | 0.99 | 5.95 | 2.64 |  |
| 1qpe | A | 3A4O | 74.26 | 1.00 | 3.86 | 1.98 |  |
| 2ypi | A | 3PF3 | 48.79 | 0.98 | 1.67 | 2.64 |  |
| 1srf | A | 2ZSC | 51.28 | 0.98 | 3.38 | 2.77 |  |
| 1inc | A | 1BRU | 58.51 | 1.00 | 2.55 | 2.77 |  |
| 2ifb | A | 1G74 | 29.23 | 0.98 | 2.31 | 3.41 |  |
| 1inc | A | 2BM2 | 37.55 | 1.00 | 4.07 | 3.44 |  |
| 1bid | A | 3V8H | 34.98 | 1.00 | 7.83 | 3.53 |  |
| 1fbp | A | 1DBZ | 44.18 | 0.96 | 3.43 | 2.91 |  |
| 1ulb | A | 2YZI | 34.78 | 0.15 | 13.87 | 8.12 |  |
| 1snc | A | 2INR | 33.33 | 0.33 | 17.88 | 11.67 |  |
| 2pk4 | A | 1JFN | 76.25 | 0.99 | 2.35 | 2.24 |  |
| 1hfc | A | 1SMP | 31.97 | 0.73 | 12.95 | 7.11 |  |
| 1apu | E | 1ER8 | 53.94 | 0.99 | 2.43 | 2.97 |  |
| 7cpa | A | 1Z5R | 45.87 | 0.98 | 1.48 | 3.06 |  |
| 2ypi | A | 3KRS | 44.84 | 0.99 | 2.31 | 3.11 |  |
| 7cpa | A | 1AYE | 66.01 | 0.98 | 2.13 | 3.11 |  |
| 1rne | A | 3QRV | 34.52 | 1.00 | 7.88 | 3.61 |  |
| 2ctc | A | 3DGV | 44.19 | 0.99 | 2.07 | 3.13 |  |
| 1inc | A | 1ELT | 67.93 | 0.98 | 1.45 | 3.15 |  |
| 2tmn | E | 4GER | 55.81 | 0.97 | 1.10 | 3.17 |  |
| 1rne | A | 1TZS | 40.67 | 0.97 | 8.54 | 3.17 |  |
| 1apu | E | 2RMP | 28.25 | 1.00 | 3.41 | 3.66 |  |
| 2ctc | A | 1JQG | 31.53 | 0.96 | 2.33 | 3.68 |  |
| 1hfc | A | 1FBL | 91.08 | 0.99 | 1.50 | 2.41 |  |
| 1ida | A | 1FMB | 33.98 | 0.97 | 1.88 | 3.74 |  |
| 1hyt | A | 1NPC | 73.1 | 0.99 | 1.44 | 2.44 |  |
| 7cpa | A | 1DTD | 64.9 | 0.98 | 1.31 | 3.23 |  |
| 2ctc | A | 1PCA | 87.3 | 1.00 | 0.95 | 2.49 |  |
| 1hfc | A | 2XS4 | 44.94 | 0.97 | 3.24 | 3.23 |  |
| 4dfr | A | 4EIL | 30.54 | 0.99 | 5.16 | 3.76 |  |
| 1mrg | A | 3MRW | 93.09 | 1.00 | 1.05 | 2.50 |  |
| 7cpa | A | 1PCA | 88.27 | 1.00 | 1.39 | 2.73 |  |
| 7cpa | A | 1JQG | 30.57 | 0.96 | 2.69 | 3.88 |  |
| 1ivd | A | 1NMB | 48.59 | 0.99 | 2.08 | 3.25 |  |
| 1fbp | A | 3AL0 | 46.15 | 0.08 | 17.59 | 6.48 |  |
| 4dfr | A | 2D0K | 94.94 | 0.99 | 1.99 | 2.85 |  |
| 1fbp | A | 1E3D | 31.03 | 0.26 | 20.86 | 12.47 |  |
| 1qpe | A | 1QCF | 76.38 | 1.00 | 6.64 | 2.90 |  |
| 2h4n | A | 3JXG | 29.7 | 0.99 | 4.01 | 3.95 |  |
| 1inc | A | 2GD4 | 29.92 | 1.00 | 2.85 | 4.04 |  |
| 1pso | I | 1MPP | 31.01 | 1.00 | 7.58 | 4.07 |  |
| 1fbp | A | 1FRP | 96.06 | 1.00 | 1.49 | 2.91 |  |
| 1mrg | A | 1J4G | 63.82 | 1.00 | 1.41 | 3.39 |  |
| 1pso | I | 3FNS | 26.44 | 0.98 | 6.11 | 4.28 |  |
| 1rbp | A | 1S2P | 48.65 | 0.21 | 14.34 | 6.93 |  |
| 1snc | A | 4KJN | 89.63 | 0.99 | 3.59 | 3.03 |  |
| 2h4n | A | 4LU3 | 36.12 | 0.99 | 3.75 | 4.37 |  |
| 1srf | A | 2JGS | 44.12 | 0.28 | 11.43 | 8.72 |  |
| 1ulb | A | 3KHS | 50.36 | 0.95 | 3.82 | 3.40 |  |
| 1rbp | A | 2L9C | 17.9 | 0.92 | 15.58 | 4.50 |  |
| 1cdo | A | 1RJW | 31.12 | 0.97 | 8.93 | 4.52 |  |
| 1fbp | A | 3IFA | 73.25 | 0.99 | 1.69 | 3.07 |  |
| 1snc | A | 1OLA | 36.73 | 0.34 | 14.70 | 12.61 |  |
| 1rbp | A | 4MTP | 31.82 | 0.36 | 16.84 | 12.92 |  |
| 2ctc | A | 2BOA | 61.24 | 0.99 | 1.26 | 3.40 |  |
| 1hfc | A | 3DSL | 25.74 | 0.76 | 11.72 | 7.34 |  |
| 1rbp | A | 1AQB | 93.71 | 0.99 | 1.27 | 3.09 |  |
| 2sim | A | 4BBW | 30.45 | 0.88 | 5.37 | 7.70 |  |
| 1stp | A | 2JGS | 42.86 | 0.27 | 14.83 | 9.50 |  |
| 1mrg | A | 2OQA | 70.35 | 0.91 | 1.45 | 3.14 |  |
| 1rbp | A | 1IIU | 87.21 | 0.98 | 1.50 | 3.25 |  |
| 1acj | A | 1DX4 | 37.11 | 0.99 | 1.57 | 4.53 |  |
| 1qpe | A | 1K9A | 43.28 | 0.98 | 5.34 | 3.41 |  |
| 4dfr | A | 2KGK | 39.38 | 0.99 | 5.16 | 4.55 |  |
| 2pk4 | A | 3KIV | 85.9 | 0.96 | 1.97 | 3.32 |  |
| 5cna | A | 3UJO | 38.33 | 0.48 | 14.50 | 13.62 |  |
| 1okm | A | 1V9E | 80.71 | 0.98 | 1.22 | 3.33 |  |
| 1rob | A | 3HMN | 60.61 | 0.20 | 17.00 | 15.27 |  |
| 1bid | A | 1B02 | 35.64 | 0.99 | 2.72 | 4.62 |  |
| 1blh | A | 3W4O | 33.59 | 1.00 | 2.05 | 5.16 |  |
| 1acj | A | 3O9M | 53.48 | 1.00 | 1.83 | 3.48 |  |
| 1pso | I | 2PSG | 84.97 | 1.00 | 4.50 | 3.37 |  |
| 1stp | A | 4EKV | 92.56 | 0.99 | 8.06 | 3.52 |  |
| 2ifb | A | 1EII | 36.67 | 0.66 | 4.38 | 8.19 |  |
| 2h4n | A | 2W2J | 40.86 | 0.95 | 4.63 | 3.49 |  |
| 2ifb | A | 1A57 | 87.79 | 0.99 | 11.38 | 3.59 |  |
| 1ulb | A | 4NS1 | 43.84 | 0.95 | 2.61 | 3.58 |  |
| 1rne | A | 1IBQ | 24.7 | 0.98 | 8.28 | 5.48 |  |
| 2tmn | E | 4M46 | 22.48 | 0.41 | 16.14 | 14.42 |  |
| 3ptb | A | 1FXY | 55.02 | 1.00 | 1.23 | 3.58 |  |
| 1byb | A | 2XFF | 66.6 | 0.99 | 2.24 | 3.66 |  |
| 1mtw | A | 1FXY | 55.02 | 1.00 | 1.38 | 3.74 |  |
| 1stp | A | 1P6G | 36.67 | 0.24 | 9.33 | 14.55 |  |
| 2ypi | A | 3TH6 | 55.1 | 0.99 | 1.95 | 3.88 |  |
| 1qpe | A | 3C4C | 32.25 | 0.96 | 8.15 | 5.59 |  |
| 2ypi | A | 1WYI | 53.04 | 1.00 | 2.49 | 4.07 |  |
| 7cpa | A | 1QMU | 21 | 0.84 | 17.70 | 8.22 |  |
| 2tmn | E | 1NPC | 73.1 | 0.99 | 1.68 | 3.61 |  |
| 1pso | I | 1G0V | 41.3 | 0.98 | 2.99 | 4.16 |  |
| 1ulb | A | 1TCU | 49.28 | 0.95 | 2.41 | 4.17 |  |
| 1mrg | A | 1D8V | 53.69 | 0.99 | 2.33 | 4.25 |  |
| 1hfc | A | 1A86 | 66.24 | 0.98 | 1.60 | 4.51 |  |
| 1gca | A | 2HPH | 94.17 | 1.00 | 0.77 | 3.83 |  |
| 1bid | A | 4F2V | 93.54 | 0.99 | 2.56 | 3.86 |  |
| 1ida | A | 1BDR | 54.55 | 0.99 | 1.40 | 5.14 |  |
| 1rob | A | 3ZBV | 34.15 | 0.98 | 4.80 | 5.96 |  |
| 1acj | A | 1AKN | 31.64 | 0.99 | 10.57 | 6.06 |  |
| 1qpe | A | 3DK6 | 48.68 | 0.97 | 5.32 | 5.25 |  |
| 1ivd | A | 1INF | 32.11 | 0.93 | 4.28 | 6.08 |  |
| 4phv | A | 3S43 | 89.9 | 0.99 | 1.05 | 3.98 |  |
| 1rob | A | 1Z9M | 47.62 | 0.33 | 16.12 | 15.50 |  |
| 1gca | A | 2X7X | 26.98 | 0.88 | 11.72 | 8.68 |  |
| 1rob | A | 1QMT | 27.61 | 0.98 | 5.97 | 6.09 |  |
| 1pso | I | 3PSG | 85.28 | 1.00 | 4.43 | 4.17 |  |
| 1srf | A | 4BX5 | 86.15 | 0.99 | 3.40 | 4.32 |  |
| 1hew | A | 3CB7 | 38.66 | 0.91 | 3.42 | 6.09 |  |
| 1inc | A | 1FI8 | 32.92 | 1.00 | 4.45 | 7.18 |  |
| 1stp | A | 4BX5 | 88.64 | 0.99 | 3.24 | 4.44 |  |
| 1ulb | A | 1LV8 | 88.73 | 0.98 | 4.67 | 4.63 |  |
| 1okm | A | 2W2J | 40.86 | 0.95 | 4.74 | 5.26 |  |
| 1imb | A | 2BJI | 88.28 | 1.00 | 1.17 | 4.69 |  |
| 4phv | A | 3OGP | 30.19 | 0.94 | 2.28 | 7.34 |  |
| 1srf | A | 3EW1 | 35.09 | 0.94 | 12.63 | 7.49 |  |
| 5cna | A | 2FMD | 56.14 | 0.48 | 13.31 | 19.17 |  |
| 1hyt | A | 4M46 | 22.48 | 0.41 | 17.65 | 15.54 |  |
| 1srf | A | 2C4I | 33.82 | 0.53 | 12.10 | 9.088 |  |
| 4phv | A | 1SIP | 51.52 | 0.99 | 1.79 | 5.53 |  |
| 1hew | A | 1DZB | 94.57 | 0.99 | 1.67 | 5.03 |  |
| 2ctc | A | 1QMU | 21.71 | 0.84 | 15.98 | 11.01 |  |
| 1ida | A | 1BDQ | 57.58 | 0.99 | 1.56 | 5.70 |  |
| 1ida | A | 2OKV | 36.36 | 0.32 | 13.66 | 18.22 |  |
| 3gch | A | 4F4O | 28.52 | 1.00 | 9.75 | 8.11 |  |
| 1pso | I | 1HTR | 52.28 | 1.00 | 5.93 | 5.96 |  |
| 1snc | A | 2W8V | 25.49 | 0.74 | 14.46 | 14.32 |  |
| 1pso | I | 1AM5 | 59.82 | 1.00 | 2.44 | 6.17 |  |
| 1hew | A | 2RSC | 43.22 | 0.91 | 3.23 | 6.19 |  |
| 4phv | A | 1NSO | 28.72 | 0.87 | 8.58 | 15.19 |  |
| 1cdo | A | 4DL9 | 57.56 | 1.00 | 2.83 | 6.57 |  |
| 4phv | A | 2HAH | 36.79 | 0.94 | 2.08 | 8.19 |  |
| 1ivd | A | 3SAL | 43.22 | 0.99 | 2.74 | 6.77 |  |
| 1ida | A | 3OGP | 25.47 | 0.94 | 6.25 | 8.37 |  |
| 1ivd | A | 4K3Y | 26.02 | 0.94 | 5.74 | 8.42 |  |
| 1ida | A | 1SIP | 87.88 | 0.99 | 1.52 | 5.28 |  |
| 1qpe | A | 2WTV | 25.19 | 0.97 | 12.17 | 8.47 |  |
| 1inc | A | 1PYT | 54.2 | 0.98 | 4.07 | 6.86 |  |
| 1snc | A | 2QDB | 94.81 | 0.99 | 5.05 | 5.34 |  |
| 1ida | A | 3GGU | 48.48 | 0.99 | 1.51 | 6.93 |  |
| 1rob | A | 2HKY | 42.52 | 0.95 | 6.11 | 7.51 |  |
| 4phv | A | 2MIP | 48.48 | 0.99 | 1.60 | 8.68 |  |
| 1rob | A | 4KXH | 69.35 | 0.99 | 15.81 | 10.53 |  |
| 1pdz | A | 1ELS | 64.35 | 0.99 | 2.94 | 11.73 |  |
| 4phv | A | 1MRX | 93.94 | 0.99 | 0.97 | 5.60 |  |
| 1imb | A | 1JP4 | 24.35 | 0.93 | 15.34 | 9.00 |  |
| 1pdz | A | 2PA6 | 54.63 | 0.99 | 1.87 | 15.10 |  |
| 2ifb | A | 1KZX | 81.68 | 0.99 | 1.90 | 5.95 |  |
| 1mrg | A | 4NRW | 23.89 | 0.96 | 14.02 | 9.04 |  |
| 2ifb | A | 3RSW | 32.06 | 0.99 | 3.14 | 9.10 |  |
| 1hew | A | 2EQL | 49.23 | 0.99 | 2.83 | 16.41 |  |
| 4phv | A | 3GGU | 79.8 | 0.99 | 1.71 | 6.07 |  |
| 1blh | A | 3LEZ | 35.41 | 1.00 | 2.44 | 15.56 |  |
| 4phv | A | 1BAI | 43.33 | 0.76 | 2.20 | 5.40 |  |
| 4dfr | A | 3Q1H | 79.25 | 0.99 | 5.97 | 6.24 |  |
| 2ypi | A | 2H6R | 24.09 | 0.55 | 10.27 | 15.78 |  |
| 1hew | A | 1BB6 | 61.42 | 0.98 | 1.44 | 17.50 |  |
| 1phd | A | 4APY | 24.63 | 0.95 | 4.41 | 21.53 |  |
| 1phd | A | 4C9N | 44.94 | 0.98 | 1.70 | 20.55 |  |
| 4phv | A | 3U7S | 80.81 | 0.99 | 1.22 | 7.02 |  |
| 5p2p | A | 4KF3 | 39.67 | 0.99 | 2.93 | N/A |  |
| 1rob | A | 4N4C | 84.68 | 0.99 | 10.90 | 7.57 |  |
| 1ivd | A | 2AEP | 88.92 | 1.00 | 1.46 | 9.49 |  |
| 5cna | A | 1DHK | 37.08 | 0.36 | 13.13 | 22.24 |  |
| 1pdz | A | 3I44 | 24.68 | 0.36 | 5.90 | 22.98 |  |
| 5cna | A | 2CNA | 94.51 | 1.00 | 1.85 | 9.80 |  |
| 1ida | A | 3RRX | 40.74 | 0.26 | 12.50 | N/A |  |
| 1rob | A | 4K7M | 94.35 | 0.99 | 1.82 | 9.91 |  |
| 5cna | A | 1IOA | 28.57 | 0.47 | 12.01 | 25.44 |  |
| 5cna | A | 3ZVX | 40.62 | 0.53 | 12.88 | 22.37 |  |
| 1hew | A | 2Z2F | 54.62 | 0.99 | 1.88 | 21.63 |  |
| 1hew | A | 2BQM | 59.38 | 0.98 | 1.61 | 22.86 |  |
| 1rob | A | 1H8X | 73.39 | 0.99 | 17.62 | 13.32 |  |
| 5p2p | A | 1M8T | 61.02 | 0.98 | 1.82 | N/A |  |
| 5p2p | A | 1BUN | 46.61 | 0.98 | 2.37 | N/A |  |
| 5p2p | A | 1GP7 | 52.85 | 0.98 | 2.26 | N/A |  |
| 1pdz | A | 1TE6 | 73.21 | 1.00 | 3.15 | 19.19 |  |
| 5p2p | A | 4BP2 | 76.86 | 0.97 | 3.18 | N/A |  |
| 5p2p | A | 1HN4 | 91.94 | 0.99 | 2.66 | N/A |  |
| 1rob | A | 2ZPO | 37.1 | 0.98 | 3.50 | N/A |  |
| 5p2p | A | 1T37 | 59.32 | 0.98 | 2.41 | N/A |  |
| 1pdz | A | 2Z8X | 34.25 | 0.14 | 13.00 | N/A |  |
| 5p2p | A | 1TGM | 44.07 | 0.98 | 4.05 | N/A |  |
| 2pk4 | A | 4HZH | 33.33 | 0.99 | 3.91 | N/A |  |
| 5p2p | A | 1Y6O | 89.52 | 0.99 | 1.60 | N/A |  |
| 1phd | A | 2YOO | 30.11 | 0.87 | 4.86 | 22.77 |  |
| 1blh | A | 3QHY | 40.47 | 1.00 | 2.27 | N/A |  |
| 1pdz | A | 1W6T | 49.43 | 0.99 | 2.69 | N/A |  |
| 5p2p | A | 3ELO | 74.19 | 0.99 | 3.04 | N/A |  |
| 1pdz | A | 3OTR | 59.28 | 0.99 | 1.70 | N/A |  |

**N/A : No Seed atom for generating patch for comparison.**
