# Supplementary material for: Predicting Ligand Binding Sites on Protein Surfaces by 3-Dimensional Probability Density Distributions of Interacting Atoms
Source: PLoS One. 2016 Aug 11;11(8):e0160315. doi: 10.1371/journal.pone.0160315 (PMC4981321; doi:10.1371/journal.pone.0160315)
Supplement: S10 Table — Columns 1 and 2 from the left show the PDB code name and chain name respectively for the query protein sequence comparatively modeled with I-TASSAR package. Column 3 shows the residue numbers of actual LBS residues, each of which contains at least one heavy atom within the distance of the sum of Van der Waals radii plus the tolerance distance (0.5 Å) to any ligand heavy atom. Columns 4, 5, 6 and 7 show the residue numbers of predicted LBS residues by ISMBLab-LIG, COFACTOR, COACH and RaptorX respectively. (DOCX) [file pone.0160315.s011.docx]

**S10 Table. Prediction results of the CAMEO-LB cases by RaptorX, COACH, COFACTOR and ISMBLab-LIG. Columns 1 and 2 from the left show the PDB code name and chain name respectively for the query protein sequence comparatively modeled with I-TASSAR package. Column 3 shows the residue numbers of actual LBS residues, each of which** contains at least one heavy atom within the distance of the sum of Van der Waals radii plus the tolerance distance (0.5 Å) to any ligand heavy atom. Columns 4, 5, 6 and 7 show the residue numbers of predicted LBS residues by ISMBLab-LIG, COFACTOR, COACH and RaptorX respectively.

| PDB ID | Chain ID | Actual LBS | ISMBLab-LIG | COFACTOR | COACH | Raptorx |
| --- | --- | --- | --- | --- | --- | --- |
| 4yvn | A | 12,107,113,114,118,121,133,135,188,243,244,250,266,267,268,292,293,300,317,328,332,333,341,359,360,363,366,406,408,419,424,429,431,457,459,463,476,478,479,480,483,484,485,487,491,492,497,510 | 226,227,228,229,259,260,262,321,322,323,375,376,384,385,386,414,415,416,417,418,419,494,497 | 105,107,153,155,422,424,491,493 | 105,107,153,155,422,424,491,493 | X |
| 4zu5 | B | 13,20,22,35,37,50,72,96,98,116,121,123,125 | 37,39,49,51,53,56,58,97,99,110,112,116,121 | 35,37,48,51,58,97,110,116,120 | 35,37,48,49,51,116,121,123 | 35,37,48,49,51,97,99,116,121 |
| 4zv8 | A | 43,44,46,47,50,51,98,101,113,114,115,121,125,132,179,206,212,215,219,220,222,223,224,295,297,298,299,302,303,306,357,362,363,367,369,392,428,429,430,434,436,437,438,442,477 | 108,113,114,115,121,124,125,128,129,132,137,138,179,264,268,271,283,291,293,294,295,297,298,299,302,303,363,367,429,434,435,436,437,438,440,441,442,444,445 | 115,294,297,298 | 79,94,95,102,106,276,279,280,283,284,287,338,343,344,347,348,350,409,410,411,415,416,417,418,419,422,423,427 | 79,85,89,94,95,96,102,106,113,160,275,276,278,279,280,283,284,343,344,348,350,373,409,410,411,415,417,418,419,423 |
| 4zvm | B | 11,15,16,17,18,20,66,67,68,102,103,104,105,106,117,122,126,128,147,148,149,150,154,155,161,178,192,193,194,200,201,204 | 11,18,103,104,105,106,149,150,154,155,161,166,189,190 | 105,106,149,161,193,194 | 11,15,16,17,18,20,102,103,104,105,106,147,148,149,150,155,192,193,200 | 11,15,16,17,18,20,102,103,104,105,106,147,148,149,150,155,192,193,200 |
| 5a2c | A | 98,100,101,140,143,178,180,211,213,214,242,309,310,358,362 | 85,100,101,135,137,140,174,175,177,180,211,213,214,216,217,242,244,264,304,309,310,344 | 98,100,140,180,211,213,214,242,244,309,310 | 111,151,188,191,222,224,225,253,255,320,321 | 109,111,112,151,188,189,191,222,224,225,227,228,253,255,320,321,369,373 |
| 5aei | C | 23,25,277,280 | 75,114,117,156,159,201,240,243 | 123,124,125,130,159,162,163,198,201 | X | 141,142,143,144 |
| 5b39 | G | 42,45,71,91,93,108,120,122,158,183,252,254,256 | 122,124,125,156 | 116,165,167,199,200,201,227,228,230,276,278 | 233,244,283 | 42,71,91,93 |
| 5ciy | A | 18,19,20,21,22,23,24,39,40,41,42,60,61,62,78,80,81,85,86,100,114,121,137,141,146,147,163,165,172,196,197,198,199,219,220,221,281,286,287,288,290,303,304,305,306 | 16,17,18,19,20,21,22,23,24,41,59,60,76,77,78,79,80,81,82,101,119,121,163,253,303,304,305,306 | 79,81,82,85,86,87,88,97,119,120,121,162,163,165,226,228,237,240,242,249,250,252,253,254,255,256,303,304 | 18,19,20,21,22,23,24,39,40,41,42,59,60,61,78,80,97,100,304,305 | 86,87,162,226,228,237,240,242,249,250 |
| 5djz | A | 241,243,244,245,246,258,260,262,264,265,295,297,299,301 | 243,259,313,319,376 | 241,243,244,246,272,260,262,264,265,295,297,299,301,334 | 21,23,42,44,81 | 15,44,45,75,76,77,79,81 |
| 5e4t | A | 7,11,12,13,14,20,31,44,50,52,53,54,57,58,59,61,62,63,70,74,84,100,105,106,107,109,117,118,119,121,131,139,140,185,186,199,230,232,235,236,243,247,268,271,279,281,282,285,286,287,289,296,297,298,325,326,330,331,334,335,344,346,348,358,361,362,385,388,389,393,406,416,417,422,424,426,440,456,457,478,493,494,496,499,501,502,512,513,514,517 | 69,70,71,72,74,78,80,81,83,84,116,117,118,119,121,122,123,199,200,226,232,233,278,279,286,287,288,289,290,291,327,330,331,333,334,341,359,360,362,363,398,399,400,422,432,439,440,441,442,445,450,458,516,517,524,528,541 | 72,84,117,118,119,121,199,200,288,290,330,331,440 | 84,117,118,119,121,200,201,290,331,440 | 338,340,342,343 |
| 5e6f | B | 79,80 | 8,9,10,11,13,40,61,62,64,70,131 | 41,65,68,69,71,72,75,76,79,80,85 | 61,62,63 | 10,11,12,13,15,33,61,101,121,123,128,130,131 |
| 5elf | J | 3,11,12,16,17,18,44,45,46,47,48,76,77,79,80,81,92,93,94,103 | 88,96,97,98,99 | 14,51,56,57,61,88,90 | 12,13,51,56,57,61,88,90,91 | 12,51,56,57,61,88,90,91 |
| 5eq9 | D | 149,150,151,203,204,205,206,218,222,246,263,264,265,266,270,282,312,313 | 105,126,127,128,145,146,148,149,150,151,152,153,155,218,242,243,244,245,246,248,252,257,265,268,269,270 | 146,148,149,150,151 | 79,97,99,100,101,102,221 | X |
| 5esx | B | 76,105,106,107,109,110,111,112,113,137,160,161,166,167 | 48,49,83,84,85,105,106,107,108,109,111,112,113,114,117,159,160,161 | 107,109,110,111,137,159,160,161,166,167 | 55,56,57,80,113,115,116,117,118,119,143,165,166,167,172,173,179 | X |
| 5fhf | A | 4,5,10,30,31,32,33,34,35,36,187,188 | 4,10,25,27,28,29,30,31,32,33,34,35,36,38,54,55,56,57,58,61,66,68,69,105,106,109,110,114,140,141,142,143,145,146,147,168,180,181,182,188,265,267,268,270,286,295,296,297,298,299,327,329,331,361,364,365,367,368,369,390 | 10,30,31,32,33,34,35,36,145,187,188,390 | 4,5,10,30,31,32,33,34,35,36,145,187,188,365,390 | 54,55,56,57,66,68,148,151,237,267,268,359,361,362 |
| 5fii | D | 43,44,46,47,48,61,62,63,64,65,67,77,79 | 87,111 | 43,44,46,47,48,67,77,79 | 25,28,29,30,31,32,51,61,63 | 12,13,14,18,20,22,64,91,92,93,95,101 |
| 5fkp | B | 9,10,11,15,35,36,37,51,52,54,63,64,65,95,96,97,99 | 37,38,51 | 1,8,10,11,12,32,26,31,33,34,53,54,55,56,57,60,62,65,98 | 8,10,11,13,14 | 57,63 |
| 5fkp | A | 10,12,19,20,23,24,25,28,30,31,34,42,47,63,70,73,77,100,102,113,114,118,128,129,130,131,133,143,150,161,163,164,165,167,168,171,192,209,217,218,239,240,241,243,244,245,246,266,267,268 | 73,77,80,118,133,150,156,160 | 10,12,14,28,30,66,69,70,73,76,77,80,81,100,102,118,133,150,153,156,159,160,164,171 | 10,12,14,28,30,40,47,63,66,69,70,73,76,77,80,81,100,102,118,133,150,153,155,156,159,160,163,171 | 129,130,131,161,165 |
| 5hes | B | 22,27,30,43,44,45,57,63,66,68,80,82,83,84,85,88,140,144,146,148,150,151,152,153 | 9,22,23,24,25,30,43,44,45,51,52,54,66,71,82,83,84,85,88,89,92,135,136,137,140,150,151,152 | 23,24,25,43,83,84,85,89,137,140,151 | 20,28,41,42,43,48,54,55,58,63,64,78,80,81,82,83,84,86,127,129,138,148,149 | 20,21,25,28,41,43,64,78,80,81,82,83,84,86,87,138,148,149 |
| 5hq8 | B | 14,15,16,124,130,132,151,152,159,161,162,171,180,181,184,186,192,202,203,204,205,206,214,216,238,239,240,242,257,259,366,368 | 29,186,187,188,189,190,213,214,215,224,241,242,243,244,252,297,302,329,332,333,336,366,367,368,372,375,404,405,410 | 14,15,16,124,130,132,180,181,202,203,204,205,206,239,257,259 | 18,19,20,128,134,136,184,185,206,207,208,209,210,243,261,263 | 18,19,20,134,136,184,185,206,207,209,210,243,261,263 |
| 5ihp | B | 21,22,23,24,25,26,192,220,221,222,223 | 20,21,22,23,24,25,26,48,50,52,53,55,132,192 | 20,21,23,24,25,26,192,220,221,222,223,226,227 | 21,23,24,25,26,191,192,220,221,222,223,226,227,230 | 21,22,23,24,25,26,192,221,222,223,225,232,234,235 |
| 5in3 | B | 67,73,80,81,90,95,97,98,99,108,109,118,119,120,121,123,128,131,133,134,139,154,155,156,159,171,172,173,180,186,188,190,216,219,223,224,226,227,250,258,260,261,262,263,265,266,272,279,283,296,298,309,310,322,327,334,335,337,338,339,340,346,347,348,349,356,359,360 | 35,37,38,40,45,102,122,128,169,178,188,190,191,192,194,196,197,202,221,245,246,248,249,252,254,297,319,321,323,325,340,343,344,345,346 | 73,74,80,81,95,97,98,171,173,179,180,181,188,190 | 95,96,102,103,117,119,120,193,195,201,202,203,210,212 | 95,102,103,117,119,120,121,150,193,195,202,203,208,210,212 |
