# Supplementary material for: Predicting Ligand Binding Sites on Protein Surfaces by 3-Dimensional Probability Density Distributions of Interacting Atoms
Source: PLoS One. 2016 Aug 11;11(8):e0160315. doi: 10.1371/journal.pone.0160315 (PMC4981321; doi:10.1371/journal.pone.0160315)
Supplement: S1 Text — (DOCX) [file pone.0160315.s012.docx]

**S1 Text: Supplemental Methods**

***Amino acid conformation clustering***

Amino acids in proteins are limited in structural diversity. Protein structures are determined by main chain and side chain torsion angles of the constituent amino acids. The distributions of the torsion angles are clustered around prevalent conformational centers, instead of spreading continuously over the torsion angle space. The main chain torsion angles are clustered at the α- and the β-regions in the Ramachandran plot; the distributions of the side chain torsion angles are also concentrated on only a few allowable regions, depending on the chemical constituents of the side chain [1, 2]. Moreover, the distribution of each of the side chain torsion angles is dependent on the torsion angles of the backbone of the amino acids [3]. Thus, amino acid conformations in proteins can be organized into limited sets of clusters based on the main chain and side chain torsion angle set of each of the amino acid types, allowing interacting atom pair database retaining conformational information of the parent amino acids.

Database for non-covalent interacting atom pairs in proteins was organized according to parent amino acid conformational types. To cluster amino acid conformations into a limited set of clusters for each type of amino acid, we assigned torsion angles to each of the amino acids in known protein structures with the computer program DSSP [4] and MOLEMAN 2 [5]. For each type of amino acid from the protein structure entries in PDB, a set of vectors with torsion angle elements in degree ({*φ*, *ψ*, χ*_1_*, …, χ*_i_*}, where *φ*, *ψ* are backbone torsion angles and χ*_i_* are side chain torsion angles as defined conventionally) was established; amino acid residues with incomplete structure were excluded from the data sets. The vectors were used as input to the fuzzy c-means algorithm [6] for clustering. The number of the clusters was determined as the minimal integer satisfying the condition that increasing the number of clusters beyond this minimal integer made little change to the partition index and separation index – two fuzzy c-means algorithm indexes describing the relative mean distance within and between clusters [7]. To augment the optimal decision on cluster numbers, we calculated the distribution of the intra-cluster RMSD (root mean squared deviation) in Å for superimposed amino acid structures between cluster members and the centroid conformation within a cluster for each cluster sets. The convergence of this intra-cluster RMSD to a minimal RMSD provided a more structure-related reference in contrast to the torsion angle-based structural descriptors in determining the optimal cluster number. After the determination of the cluster numbers, the centroid conformation of each of the clusters was determined as the center of mass of the vectors in the cluster. The number of clusters, the torsion angles of the centroid conformations, and the distribution information of the members in the clusters are listed in Supplementary Table 6 of Yu et al [8].

***Protein atomistic non-covalent interacting database***

Atomistic contact interactions in proteins of known structures were organized into a database containing non-covalent atomistic interaction information for atom pairs in protein structures. The general methodology is outline in Supplementary Figure 1 of Yu et al [8]. For each of the atoms in residue X of a protein, the non-covalent interacting atoms were recorded as the following: Following the work of Laskowski et al. [9], for each atom (P) in residue X, the relative location of the atom P was defined with two consecutive atoms R and Q, where R is covalently linked to P, and Q is covalently linked to R. Atom R was set at the origin of the reference coordinate system; atom P was located on the z-axis; atom Q was on the z-x plane of the reference coordination system. In principle, all non-covalent interacting atoms to atom P were recorded in the database with the reference coordination system. In this work, only non-covalent atomistic interactions in protein interiors were organized into the atomistic interaction database: First, a protein structure was randomly separated into two parts by cleaving at a random peptide bond. Interface residues with solvent accessible surface area (SASA) change more than 40% of the total SASA due to the separation of the two protein halves were considered for non-covalent atomistic interactions. The solvent accessible surface area (SASA) for each of the amino acid residues was calculated with DSSP. Only the atoms from the other half of the proteins were recorded for interacting with atom P when the pairwise distance between the two atoms was less than 5 Å. Atoms within 9 consecutive residues from the N and C directions of the atom P were excluded as interacting atoms to the atom P. This was to record the atomistic contact interactions mimicking the interactions in protein-protein interfaces. After all the interface residues were surveyed, the protein structure was again randomly separated at a different cleavage site and the survey for the atomistic contact interactions of each of the interface residues was repeated. This process repeated 40 times for each of the protein structures in the 9468 non-redundant protein structures with less than 60% sequence identity [10]. After the survey on all the non-covalent interacting atom pairs, the database was organized into a large number of files; each file is specific to an amino acid type, a conformational type based on the torsion angle vector of the amino acid, an atom type in the parent amino acid, and the interacting atom type. The structure of the data files facilitates the speedy random access of the database in predicting distribution of probability density maps (PDM) of non-covalent interacting atoms as described in the following section. Atoms in the 20 natural amino acids are assigned to one of the 30 interacting atom types found in proteins plus the crystal water oxygen as the 31^st^ atom type (Supplementary Table 7 of Yu et al [8]).

Water oxygen distributions around the surface amino acids in 915 non-redundant protein structures solved to high resolution (resolution<1.5Å, sequence identity less than 30%, different graph topology and subunit structure) [11] were recorded with the same P-R-Q reference coordination system and were stored in the same file system as described above. Water oxygens within 3.2 Å radius (within hydrogen bonding distance) to the interacting amino acid atoms were recorded in the database. This database was used for evaluating the desolvation penalties and water-mediated interactions in protein-protein interaction interfaces.

***Predicting probability density maps (PDM) of non-covalent interacting atoms for protein surfaces***

A probability density map (PDM) of a non-covalent interacting atom type is a three-dimensional distribution of likelihood for the type of atom to appear around protein surface amino acids. In this work, the PDMs were reconstructed from the interacting atom pair databases described in the previous section for the 31 interacting atom types shown in Supplementary Table 7 of Yu et al [8]. The general methodology is outline in Supplementary Figure 1 of Yu et al [8].

To construct a PDM for an interacting atom type on a target protein surface, the computer algorithm first enclosed the target protein structure in a rectangular box clearing the structure by a margin of at least 7 Å from all sides of the protein’s edge. The three-dimensional rectangular box was then gridded with 0.5 Å per unit in three-dimensional space. This grid size was a balance between the resolution of the PDM and the computational resources needed for the PDM construction. The grid points enclosed within the Connolly surface [12] of the target protein were masked from assigning PDM.

The torsion angles of side chain and main chain of all the amino acids in the protein structure were calculated with MOLMAN2 and DSSP respectively. For each of the amino acid residues in the protein, the conformational type of the amino acid X was determined by the torsion angle vector, which had the least Euclidean distance to the centroid conformation of the assigned conformational cluster. With the assignment of the conformational type for each of the amino acids in the protein structure, the non-covalent interacting atoms around each atom P in the protein structure were allocated from the database according to the atom type of P, the assigned three-atom reference system P-R-Q as described in the previous section, the amino acid type of the parent residue containing atom P, and the conformational type of the parent amino acid. Interacting atoms outside the sphere with the radius equal to the sum of the van der Waals radii of the interacting atom and atom P plus a tolerance of 0.5 Å were not included as the interacting atoms with atom P. The coordinates of the allocated interacting atoms were transformed to the coordination system of the protein structure and mapped around the protein surface. An atom of non-covalent interaction was to be mapped only once for which the distance of the atom to P was the shortest. 31 PDMs were constructed from all the interacting atoms allocated for all the protein atoms (30 atom types) in the protein structure.

In order to keep PDMs high in information content and low in noise from irrelevant interactions, two strategies have been implemented. First, allocation of interacting atoms according to the amino acid conformational type (as described above) is crucial for retaining information content in PDMs. Alternative approach for PDM construction with interacting atoms allocated from mixed amino acid conformational types would lead to loss of fidelity in relative orientations of the interacting atoms, resulting in spreading PDMs around dihedral bonds. We found that mapping interacting atoms obtained from an atom in an amino acid conformational type onto the surroundings of the atom in another amino acid conformational type led to serious spatial distortion of the distribution of the interacting atoms. Second, only interacting atomic pairs in the database are used for PDM constructions. Atom pairs in the database were recorded by a threshold of distance in proximity. But frequently, many of such distributions of proximal atom pairs are results of covalent structures of non-interaction pairs in a nevertheless stable structure. In this work, non-interacting atomic pairs were eliminated with a filter Table as shown in Supplementary Table 8 [13] of Yu et al [8]. Only the atomic pairs with the value in the matrix of the Table less than -0.1 were considered as interacting pairs and only these interacting atoms were included in the PDM constructions.

PDMs were constructed by mapping the interacting atoms allocated from the database as described in the previous paragraphs to the 3D grid system. To construct the PDM, each of the interacting atoms was distributed to 8 nearest grid points; the portion of the distribution was normalized by the database redundancy and was inversely proportional to the square of the distance from the atom to the grid:

$v_{ji}=\frac{1}{p_{i}n}\frac{\frac{1}{d_{ji}^{2}}}{\sum_{k=1}^{8} \frac{1}{d_{ki}^{2}}}$ (1)

, where *v_ji_* is the value to be accumulated at a nearest grid point *j* for interacting atom *i*; *d_ji_* is the distance of grid point *j* to the center of the interacting atom *i*; grid points indexed *k*=1~8 are the nearest grids to the atom *i*; *n* is the number of residues collected in the database for the amino acid in the target protein with the conformational type defined by the torsion angle vector; *p_i_* is the background probability for atom type *i* to appear in all protein structures (when calculating water oxygen PDM, *p_i_* equals to 1). The factor 1/*n* in the Equation is to normalize the interacting atom density according to one conformation for each of the residues in the target protein and the background probability *p_i_* is to normalize the PDM based on the appearance frequency of the atom type *i* in proteins (except for water oxygen). The PDM for each of the interacting atom types was additively accumulated to completion as each of the atoms in the target protein surface finished contributing to the PDMs.

PDMs constructed for 31 interacting atomic types on the surface of 20 natural amino acids and their various conformations are displayed online: <http://ismblab.genomics.sinica.edu.tw/introduction/diaa/>.

***PDM-based attributes as inputs for machine learning algorithms***

Protein atoms were categorized into 30 atom types, and machine learning models were trained for each of the atom types. The input attributes for the machine learning models were calculated from the PDMs on the protein surface. In order to prevent self-information used in the predictions, all the interacting atom information involving the query protein were eliminated from the corresponding PDMs, mimicking a blind test for the protein structure that is not in the known protein database.

For each atom *i* on the surface of the query protein (solvent accessible surface area of atom *i* > 0), the PDM values associated with the grids within 5 Å radius centered at the atom were summed in Equation (2).

$S_{i,j}=\sum_{k}^{r_{i,k}\leq5Å} g_{k,j}$ (2)

, where *S_i,j_* is the PDM sum for interacting atom type *j* at atom *i*; *r_ik_* is the distance between atom *i* to a grid point *k*; *g_k,j_* is the PDM value of interacting atom type *j* at grid point *k*.

*A_i,j_* associated with each atom *i* was calculated with Equation (3).

$A_{i,j}=S_{i,j}+\frac{\sum_{k}^{d_{i,k}\leq10Å} S_{k,j}d_{i,k}^{-2}}{\sum_{k}^{d_{i,k}\leq10Å} d_{i,k}^{-2}}$ (3)

, where *S_i,j_* is defined in Equation (2); *d_ki_* is the distance between atom *i* and atom *k*.

The attribute set (*a_i,j_*) for the machine learning models on atom *i* were derived from *A_i,j_* with the following scaling scheme:

if *A_i,j_* > *M_max,j_* then *a_i,j_*=1; otherwise

if *A_i,j_* < *M_min,j_* then *a_i,j_*=0; otherwise

$a_{i,j}=\frac{A_{i,j}-M_{\min,j}}{M_{\max,j}-M_{\min,j}}$ (4)

, where *M_max,j_* is the median of the distribution of the maximal *A_i,j_* from each of the proteins in the protein dataset and *M_min,j_* is the median of the distribution of the minimal *A_i,j_* of the proteins in the protein dataset.

***Prediction of ligand binding sites with the ANN_BAGGING machine learning algorithm***

Only the atoms on protein surfaces with SASA (solvent accessible surface area) greater than zero were used as training or testing cases. For each of the 30 atom types on proteins, machine learning models were trained and validated with the negative and positive cases found in the S5010 dataset. A positive case was a protein atom within 4.5 Å to any atoms of the ligand in the protein-ligand complex. For unbound proteins in S48ub dataset, the positive cases were determined according the assignments in the corresponding protein-ligand complexes in the S48b dataset.

For each of the 30 atom types, one artificial neural network model with bootstrap aggregation in machine learning (ANN_BAGGING) was used to partially eliminate learning biases resulting from imbalanced training dataset, which is due to the fact that the negative atoms for ligand binding in the training set greatly outnumber the positive atoms. The methodology [14] included multiple predictors to produce an ensemble of prediction results [15], and the final prediction was calculated by averaging with equal weight the output values from the predictors [16]. Each predictor of the ensemble was trained with a different sampling (bag) of the training set. In each bag, all of the positive cases in S5010 were included, along with 1.5 times randomly sampled negative cases. The bag number was set to twenty, which balanced computational efficiency and prediction accuracy. Each of the bags was used to train an artificial neural network model. Here, a high speed resilient back-propagation (RPROP) training technique was used [17, 18]. Resilient propagation is capable of automatic adjustment for learning rate and momentum, with the advantage of faster convergence while requiring less manual determination of network parameters. Each of the ANN_BAGGING models was trained for 1000 iterations. During the training, the model was tested on validation set (see the following section on 10-fold cross validation) after every ten training iterations. The number of training iteration which yielded the best MCC (Matthews correlation coefficient) (see below for MCC definition) on the validation set was used to determine the parameters of the predictors.

***10-fold cross validation***

All the machine learning algorithms above were trained and tested with 10-fold cross validations – 10 cross validations, each with 80% of the data from the S5010 set as the training set; 10% of the data as the validation set; the remaining 10% of the data as the testing set.

For each of the predictors, a threshold for the output activity value was determined with the validation set; positive predictions have the output activity values greater than or equal to the threshold, while the negative predictions have the output activity values smaller than the threshold. All the thresholds were determined with the validation set to optimize the MCC for the predictions.

***Prediction accuracy benchmarks***

The machining learning performance of the trained ANN_BAGGING models were benchmarked by accuracy (Acc), precision (Pre), sensitivity (Sen), specificity (Spe), F-score (Fsc) and Matthews correlation coefficient (MCC).

*$\text{Acc }=\frac{TP+TN}{TP+TN+FP+FN}\times100$* (5)

*$\text{Pre}=\frac{TP}{TP+FP}\times100$* (6)

*$\text{Sen }=\frac{TP}{TP+FN}\times100$* (7)

*$\text{Spe }=\frac{TN}{TN+FP}\times100$* (8)

$\text{Fsc}=\frac{2\times\text{Pre}\times\text{Sen}}{\text{Pre}+\text{Sen}}$ (9)

$\text{MCC }=\frac{TP\times TN-FP\times FN}{\sqrt{(TP+FP)(TP+FN)(TN+FP)(TN+FN)}}$ (10)

where TP is the number of true positives; TN the number of true negatives; FP the number of false positives; and FN the number of false negatives. Sensitivity (also known as recall) can be viewed as a measurement of completeness, whereas precision is a measurement of exactness or fidelity. MCC, as a measurement of the quality of two class classifications (positive and negative), is generally regarded as a balanced measurement which can be used even if the classes are of very different sizes as in the protein-ligand interactions. Its value ranges between -1 and 1; random correlation gives MCC of 0 while perfect correlation yields 1 in MCC.

***Confidence level for predictions***

Prediction activity (output from ANN_BAGGING models) with value ranging from 0 to 1 was normalized to prediction confidence level so that the normalized prediction results based on confidence level from various machine learning models for different protein atom types can be compared and integrated on level ground to form tentative ligand binding patches, which invariably consist of various types of protein atoms. For each of the 30 protein atom types, the machine learning outputs from the validation sets were sorted into bins of interval 0.1. The confidence level of each of the bins was calculated as the fraction of true positive over the total number of predictions in the bin. In the end, lookup-tables for the output-confidence relationships were constructed; the machine learning outputs can be converted to prediction confidence levels with these lookup-tables.

**References:**

1. Lovell SC, Word JM, Richardson JS, Richardson DC. The penultimate rotamer library. Proteins. 2000;40(3):389-408.

2. Dunbrack RL, Jr. Rotamer libraries in the 21st century. Curr Opin Struct Biol. 2002;12(4):431-40.

3. Dunbrack RL, Jr., Karplus M. Conformational analysis of the backbone-dependent rotamer preferences of protein sidechains. Nat Struct Biol. 1994;1(5):334-40.

4. Kabsch W, Sander C. Dictionary of protein secondary structure: pattern recognition of hydrogen-bonded and geometrical features. Biopolymers. 1983;22(12):2577-637.

5. Kleywegt GJ. Quality control and validation. Methods Mol Biol. 2007;364:255-72. 6. Bezdek JC. Pattern Recognition with Fuzzy Objective Function Algorithms: Kluwer Academic Publishers; 1981. 256 p.

7. Bensaid AM, Hall LO, Bezdek JC, Clarke LP, Silbiger ML, Arrington JA, et al. Validity-guided (re)clustering with applications to image segmentation. Ieee Transactions on Fuzzy Systems. 1996;4(2):112-23.

8. Yu CM, Peng HP, Chen IC, Lee YC, Chen JB, Tsai KC, et al. Rationalization and design of the complementarity determining region sequences in an antibody-antigen recognition interface. PloS one. 2012;7(3):e33340.

9. Laskowski RA, Thornton JM, Humblet C, Singh J. X-SITE: use of empirically derived atomic packing preferences to identify favourable interaction regions in the binding sites of proteins. J Mol Biol. 1996;259(1):175-201.

10. Wang G, Dunbrack RL, Jr. PISCES: a protein sequence culling server. Bioinformatics. 2003;19(12):1589-91. Epub 2003/08/13.

11. Levy ED, Pereira-Leal JB, Chothia C, Teichmann SA. 3D complex: a structural classification of protein complexes. PLoS Comput Biol. 2006;2(11):e155.

12. Connolly M. Analytical molecular surface calculation. Journal of Applied Crystallography. 1983;16(5):548-58.

13. McConkey BJ, Sobolev V, Edelman M. Discrimination of native protein structures using atom-atom contact scoring. Proceedings of the National Academy of Sciences of the United States of America. 2003;100(6):3215-20.

14. Chen CT, Yang EW, Hsu HJ, Sun YK, Hsu WL, Yang AS. Protease substrate site predictors derived from machine learning on multilevel substrate phage display data. Bioinformatics. 2008;24(23):2691-7.

15. Breiman L. Bagging predictors. Machine Learning. 1996;24(2):123-40.

16. Manning CD, Raghavan P, Schutze H. An introduction to information retrieval. Cambridge, England: Cambridge University Press; 2007.

17. Reidmiller M, Braun H. A direct adaptive method for faster backpropagation learning: the RPROP algorithm. IEEE Int Conf Neural Networks1993. p. 586-91.

18. Riedmiller M. Rprop--Description and Implementation Details Technical Report. University of Karlsruhe, 1994.

19. Xie ZR, Hwang MJ. Ligand-binding site prediction using ligand-interacting and binding site-enriched protein triangles. Bioinformatics. 2012;28(12):1579-85.

20. Huang B. MetaPocket: a meta approach to improve protein ligand binding site prediction. Omics : a journal of integrative biology. 2009;13(4):325-30. Epub 2009/08/04. 21. Huang B, Schroeder M. LIGSITEcsc: predicting ligand binding sites using the Connolly surface and degree of conservation. BMC structural biology. 2006;6:19.

22. Zhang ZM, Li Y, Lin BY, Schroeder M, Huang BD. Identification of cavities on protein surface using multiple computational approaches for drug binding site prediction. Bioinformatics. 2011;27(15):2083-8.
